# OGDA: a comprehensive organelle genome database for algae

**DOI:** 10.1093/database/baaa097

**Published:** 2020-11-28

**Authors:** Tao Liu, Yutong Cui, Xuli Jia, Jing Zhang, Ruoran Li, Yahui Yu, Shangang Jia, Jiangyong Qu, Xumin Wang

**Affiliations:** College of Life Sciences, Yantai University, No.30 Qingquan Road, Laishan District, Yantai, 264005, Shandong, P.R. China; College of Marine Life Sciences, Ocean University of China, No.5 Yushan Road, Shinan District, Qingdao 266003, Shandong, P.R. China; Southern Marine Science and Engineering Guangdong Laboratory (Zhuhai), No.9 Jintang Road, Xiangzhou District, Zhuhai 519000, Guangdong, P.R. China; College of Life Sciences, Yantai University, No.30 Qingquan Road, Laishan District, Yantai, 264005, Shandong, P.R. China; College of Marine Life Sciences, Ocean University of China, No.5 Yushan Road, Shinan District, Qingdao 266003, Shandong, P.R. China; School of Bioengineering, Qilu University of Technology (Shandong Academy of Sciences), No.3501 Daxue Road, Changqing District, Jinan 250353, Shandong, P.R. China and; College of Life Sciences, Yantai University, No.30 Qingquan Road, Laishan District, Yantai, 264005, Shandong, P.R. China; College of Marine Life Sciences, Ocean University of China, No.5 Yushan Road, Shinan District, Qingdao 266003, Shandong, P.R. China; College of Grassland Science and Technology, China Agricultural University, No.2 Yuanmingyuan Xi Road, Haidian District, Beijing, 100193, P.R. China; College of Life Sciences, Yantai University, No.30 Qingquan Road, Laishan District, Yantai, 264005, Shandong, P.R. China; College of Life Sciences, Yantai University, No.30 Qingquan Road, Laishan District, Yantai, 264005, Shandong, P.R. China

## Abstract

Algae are the oldest taxa on Earth, with an evolutionary relationship that spans prokaryotes (Cyanobacteria) and eukaryotes. A long evolutionary history has led to high algal diversity. Their organelle DNAs are characterized by uniparental inheritance and a compact genome structure compared with nuclear genomes; thus, they are efficient molecular tools for the analysis of gene structure, genome structure, organelle function and evolution. However, an integrated organelle genome database for algae, which could enable users to both examine and use relevant data, has not previously been developed. Therefore, to provide an organelle genome platform for algae, we have developed a user-friendly database named Organelle Genome Database for Algae (OGDA, http://ogda.ytu.edu.cn/). OGDA contains organelle genome data either retrieved from several public databases or sequenced in our laboratory (Laboratory of Genetics and Breeding of Marine Organism [MOGBL]), which are continuously updated. The first release of OGDA contains 1055 plastid genomes and 755 mitochondrial genomes. Additionally, a variety of applications have been integrated into this platform to analyze the structural characteristics, collinearity and phylogeny of organellar genomes for algae. This database represents a useful tool for users, enabling the rapid retrieval and analysis of information related to organellar genomes for biological discovery.

## Introduction

Algae, as the oldest taxa on Earth, include not only prokaryotic Cyanobacteria (also known as Cyanophytes in algae) but also eukaryotic algae and Charophyta of Plantae. The complex and long evolutionary process of algae is highly significant for understanding the origin and evolution of life (1). As the most complex biological group, algae have exhibited great diversity during their long evolutionary history, which makes it difficult to impose a clear definition (2). Generally, algae are photosynthetic eukaryotes that live in marine or freshwater environments. Algae are widely distributed throughout the four kingdoms of Eukaryota, including Plantae, Protozoa, Acritarcha, Chromista and Fungi. Additionally, some alga such as *Symbiodinium* in giant-clam and coral are indispensable biological components of animals (3, 4). To date, a total of 46 177 species of eukaryotic algae have been recognized and classified (AlgaeBase: https://www.algaebase.org/), with more requiring further confirmation.

Due to the rapid development of high-throughput sequencing technology, genomic analysis has played a key role in revealing the evolution of algae (5). Mitochondria and plastids (chloroplasts) are essential for several important metabolic pathways involved in photosynthesis and the energy cycles of algal cells (6–9). These two unique organelles contain distinct organellar DNA (i.e. The chloroplast genome and mitochondria genome are hereinafter referred to as cpDNA and mtDNA), which are characterized by uniparental inheritance and a compact genome structure compared with nuclear genomes. Thus, they have become efficient molecular tools for analyzing gene structure, genome structure, organelle function and evolution (10–12). Algae originated from proteobacteria and cyanobacteria, respectively, through independent primary endosymbiosis over a billion years ago, i.e. at the early stage of eukaryote evolution. Autonomous organelles were formed first, followed by a number of structural, genetic and biochemical modifications (13). An ancient endosymbiotic event occurred approximately 1.6 billion years ago, in which protoeukaryote phagocytosis cyanobacteria produced primary plastid endosymbiotic events, leading to the evolution of red algae (Rhodophyta), gray algae (Glaucophyta) and green lineages (including vascular plants, Charophytes and green algae (14). Eukaryotes experienced secondary plastid endosymbiotic events by engulfing the primitive red algae and green algae, respectively, and then evolving into diverse groups such as Ochrophyta, Cryptophyta, Dinophyceae, Euglenophyceae and Haptophyta (15). In addition, some dinoflagellates such as *Karlodinium venefium* lost their plastids during this evolutionary process and then experienced a third plastid endosymbiosis event (16). The study of endosymbiont host types based on mitochondrial genomes and endosymbiont types based on plastid genomes can further reveal the evolutionary characteristics of different algae and provide more evidence for gene transfer on the endosymbiont level as well as the nuclear/cytoplasmic interaction between endosymbiont and host (17–20).

Organellar genomes of eukaryotic algae exhibit a high level of conservation, with the majority retaining the fundamental protein-encoding and tRNA genes required to stay alive (21, 22). However, genomic differences are still observed, especially at the phylum level (22). Different algae have relatively independent symbiotic evolutionary routes for mitochondria and plastids (23, 24). Indeed, several differences have been reported in mitochondrial and plastid genomes of algae, including gene horizontal transfer among orders or families. For example, gene *orf157* exists in the mitochondrial genomes of *Laminaria* species in Phaeophyta, but is absent in *Saccharina* species (25). Moreover, genes *leuC* and *leuD* exist in the plastid genomes of the family Gracilariaceae, but are absent in those of *Melanithalia intermedia* in the same family. Gene rearrangements in the same taxa of algae suggest that potential changes occur in the transcription or cleavage patterns and independent evolution following endosymbiosis (24). In addition, organellar genomes lack some of the tRNA genes required to maintain the independent functions of mitochondria and plastids, as they are produced by the nucleus. This undoubtedly enhances the connection between the nucleus and organellar genomes. Analysis of these tRNAs would be highly significant for further understanding the sources and specific functions of different ribosomes in cells. Solving all of these research problems would provide good insights into the organellar genomes of eukaryotic algae and their functional differences.

In addition, an increase in human populations and living standards in recent years has led to the continuous release of carbon from the Earth’s crust to the atmosphere, increasing concentrations of atmospheric CO_2_ and leading to global warming, ocean acidification, biological diversity changes (26), water circulation anomalies, rising sea levels and a series of significant environmental, ecological and climate issues. As a sink for atmospheric CO_2_, the ocean absorbs more than 30% (27) of CO_2_ emissions from human activity, which leads to ocean acidification and changes in the chemical characteristics of seawater and the chemical environment of the ocean, thereby dramatically affecting the diversity and classification patterns of algae and causing population expansion and contraction (28–30). Therefore, preliminary molecular intervention methods such as the analysis of organelle genome data in order to further explore algae populations is a sensitive aspect of current research, especially for organelle genome data of different strains of the same species. With a bioinformatics boom in various fields of molecular biology, organelle genomes have been extensively applied to studies of algal genetics and systematics (12, 21). The data of algal organelle genomes (cpDNA and mtDNA) are increasing rapidly; therefore, it is important that these data are stored, interlinked and displayed in an interface. However, an integrative platform of organellar genomes for various algae that enables users to jointly examine and use relevant data has not yet been developed. Thus, there is an urgent need to construct a dedicated and more comprehensive algal organelle database. Some databases were developed into specialized databases for organelle genomes, which are very helpful for obtaining and analyzing the complete sequence of organelle genomes, such as the Plant Organelles Database (PODB) (31), Organelle Genome Database (GOBASE) (32), Chloroplast Genome Database (ChloroplastDB) (33) and Chloroplast Genome Database (CpGDB) (34). However, these databases either stop maintenance or focus only on the higher plant group, with little coverage of eukaryotic algae. With the aim of providing an organelle genomics hub for algae, we collected, analyzed, stored and visualized large-scale data to develop a user-friendly platform named Organelle Genome Database for Algae (OGDA, http://ogda.ytu.edu.cn/). Specifically, all of the plastid genomes and mitochondrial genomes of algae currently available were contained in OGDA, with 1055 plastid genomes and 755 mitochondrial genomes in total, including Rhodophyta, Chlorophyta, Ochrophyta, Glaucophyta, Cryptophyta, Charophyta, Haptophyta, Bacillariophyta, Euglenozoa, Myzozoa and Cerzozoa. The database will also be updated periodically according to newly released data both by public databases and by our own laboratory (the Laboratory of Genetics and Breeding of Marine Organism [MOGBL]). OGDA is designed to provide a convenient academic platform for algal scientists to conduct relevant research.

## Materials and Methods

### Data sources

**Figure 1.**
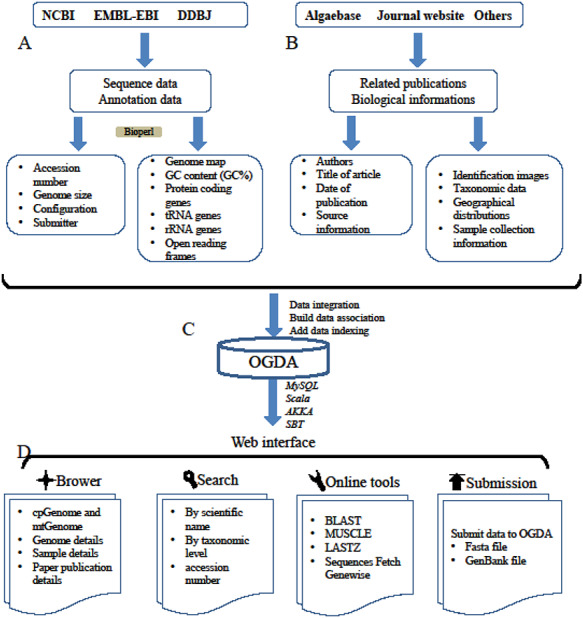
A schematic diagram of data processing for the OGDA database. (A) Data collection and data preprocessing. (B) Collection of biological information. (C) Building data association, adding data indexing and data storage in a MySQL database. (D) Overview of the web interface and usage of OGDA.

All the genome assemblies used in this study were collected from public databases and sequencing projects conducted in our laboratory (MOGBL). The complete genomes of mitochondria and plastid data were manually extracted from the public databases of National Coalition Building Institute (NCBI, https://www.ncbi.nlm.nih.gov/), the European Bioinformatics Institute (EMBL-EBI, https://www.ebi.ac.uk/) and DNA Data Bank of Japan (DDBJ, https://www.ddbj.nig.ac.jp/). The data source and the version for data download in the current study are shown in the Supplementary data (Table S1 and Table S2). This contains data measured in our laboratory (MOGBL), which acts as a representative laboratory for algae breeding research, including mitochondrial genome data of 15 different species of kelp, plastid genome data of the *Saccharina* cultivation variety ‘rongfu’ for three successive generations and organelle genome data of 35 other macroalgae (Table S3). The following process was employed for extracting and storing data (Figure 1A):

- A GenBank flat file containing a plastid or mitochondrial genome sequence was downloaded, and each genome data was manually proofread using Geneious Prime 2019 software (35) to eliminate sequences with incorrect annotation.
- The Bioperl package was used to extract basic genome information including accession number, configuration and submitter, and convert the data into CSV format for storage in the database.
- The coordinates from the annotation (coding-sequence, tRNA, rRNA and intron) were used to extract the corresponding nucleotide sequence from the genome and store the sequence in the database.

### Biological information collection

To store the biological information of algae in the OGDA database, identification images, taxonomic data, geographical distributions and sample collection information were collected from the AlgaeBase database and related publications (Figure 1B). This genomic data-related biological information was categorized and stored in OGDA MySQL (Figure 1C).

### Database implementation

The OGDA database was developed by web server Scala 2.12.2 (https://www.scala-lang.org/), SBT 0.13.17 (https://www.scala-sbt.org/), AKKA 2.12 (https://www.akka-technologies.com/) and database server MySQL 5.7.26 (https://www.mysql.com/). All data in OGDA were stored and managed using MySQL tables. The interface components of the website were designed and implemented using the Play Framework 2.6.25 (https://www.playframework.com/) and Bootstrap 3.3.0 (https://getbootstrap.com/). To visualize the genome in OGDA, we used the Jbrowser 1.12.3 (36) and Circles.js. Maps presenting the geographical distribution of phycophyta were drawn by Highmaps 6.1.0 (https://www.highcharts.com/). Multiple bioinformatics tools such as BLAST 2.6.0 (37), MUSCLE 3.8.31 (38), GeneWise 2.4.1 (39) and Lastz1.02.00 (40) were embedded in the website. The website was successfully tested in several popular web browsers, including Internet Explorer, Google Chrome and Firefox.

## Results

### Overview of OGDA

OGDA is a public hub for organellar genomes of algae, which can be obtained through the website http://ogda.ytu.edu.cn/. Currently, OGDA contains genomic data both from public databases and from our own laboratory (MOGBL). There are 755 mitochondrial genomes of 542 species belonging to 9 phyla and 1055 plastid genomes of 667 species belonging to 11 phyla in the OGDA (Table 1).

**Table 1.** Summary of OGDA data.

Data (phylum)		Mitochondrial genomes	Plastid genomes
Rhodophyta		225	321
Chlorophyta		225	401
Ochrophyta		200	113
Glaucophyta		8	9
Cryptophyta		21	13
Charophyta		14	34
Haptophyta		8	16
Bacillariophyta		45	97
Euglenozoa		7	44
Myzozoa		0	6
Cerzozoa		2	1

Multiple dynamic charts and hyperlinks can be generated in the OGDA in order to generate a user-friendly web interface (Figure 1D). Users can select randomly different taxa from the database in a number of ways. All data in the OGDA are freely available for download for academic purposes. A variety of applications were integrated into the OGDA to analyze the structural characteristics, collinearity and phylogeny of organelle genomes from algae. The database is updated simultaneously with data from major public databases such as NCBI, DDBJ and EMBL-EBI. Furthermore, a detailed user guide is provided for the efficient use of OGDA.

### Search and download functions

**Figure 2.**
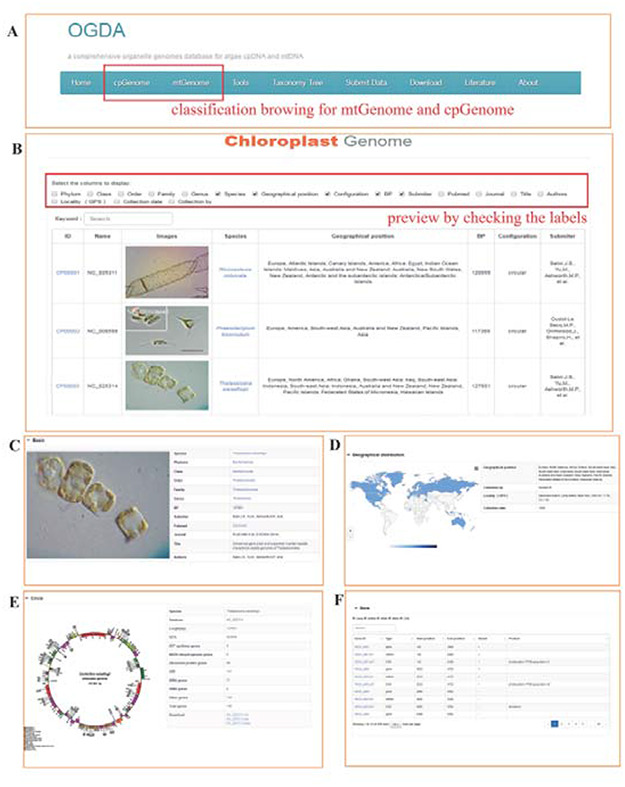
Screenshot of the database browser. (A) Navigation bar of the database, ‘cpGenome’ and ‘mtGenome’ can be browsed separately. (B) Example preview results, with the following detailed genome information: (C) basic information, including identification images, taxonomy, acceptance number, genome length (bp) and published papers; (D) geographical distribution and collection information; (E) circle map of the genome and (F) coding gene display.

To assist users in rapidly finding data of interest, a smart search system was designed. Users can search the mitochondrial or plastid genomes of the desired species using a variety of methods. First, the taxon of species can be input into the search box and searched, which shows all organelle genome information in the corresponding taxonomic level. Users can then select relevant data according to their own academic purposes. Second, users can perform a precise search by scientific name or accession number. Third, OGDA provides a classification browsing interface for mitochondrial or plastid genomes. For instance, users can obtain all mitochondrial genome information, including identification images, taxonomy, accession number, genome length (bp) and published papers by clicking the ‘mtGenome’ option. Users can preview the content of interest by checking the labels in the check boxes and clicking the Genome ID to view detailed information including the Genome Circle, geographical distribution, and all encoded genetic information (Figure 2). All of the above resources are freely available and downloadable.

### Submitting data to OGDA

More genomic algal organelle data will continue to be reported with the rapid development of sequencing technology. With the exception of data from major public databases, we also provide a task interface for researchers to upload new sequences. When uploading data, the data type (mitochondria or plastid) should be selected and the collection and classification information of the species should be completed. Published paper information about the sequence should also be provided before the sequence file is uploaded (.fasta and .gb). Finally, users can click ‘submit data’ to complete data submission.

### Genomics tools in OGDA

**Figure 3.**
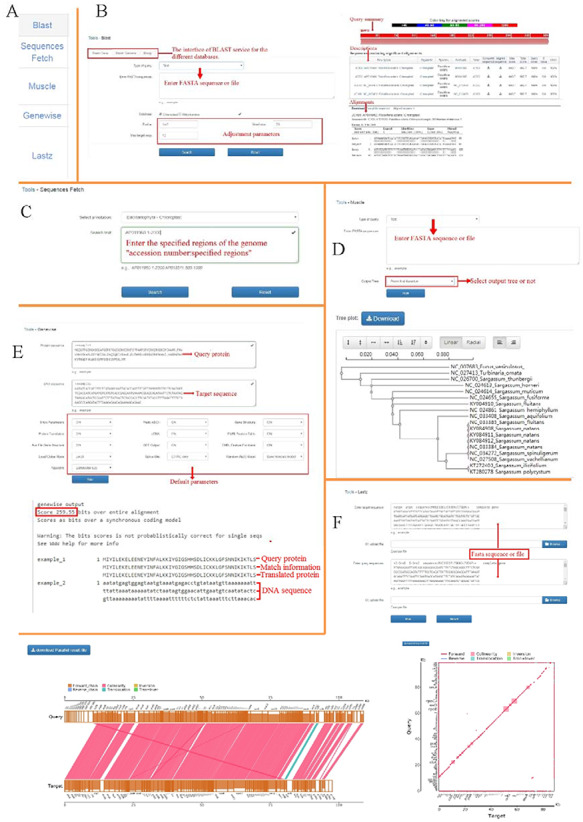
Functional genomics tools in OGDA. (A) Overview of genomics tools provided in OGDA. (B) Operation interface and results of BLAST tool. (C) An example of Sequences Fetch input interface. (D) Use MUSCLE to perform sequence alignment and output a physiological tree based on the maximum likelihood method. (E) Usage and result interpretation of GeneWise tool. (F) An example of genome synteny analysis by LASTZ, parallel and xoy plots are provided in the results.

There are several useful genomics tools in OGDA that can aid researchers in the exploration and analysis of data (Figure 3).

#### BLAST

Users can obtain annotation data in batches by performing different BLAST (37) operations. BLAST is an algorithm for comparing primary biological sequence information, such as amino-acid sequences or nucleotides sequences (DNA and/or RNA). Users can add text or files to the operation interface and click search to align the genome data in the database. BLAST results are linked to their respective gene-view pages and can be downloaded (Figure 3B).

#### Sequences Fetch

The Sequences Fetch is a suite of programs for interacting with high-throughput sequencing data that can support the retrieval of specified regions of a genome, the input format is ‘accession number: +specified regions’, such as NC_001677:1–2000bp (Figure 3C). Search results are displayed in fasta sequence.

#### MUSCLE

The MUSCLE can align hundreds of sequences instantly, with consistently better accuracy and speed than CLUSTALW (38). Users should enter the sequences (.fasta) in the text box and click ‘run’ to perform common alignment tasks. Then, the operating option of ‘Output Tree’ will generate a phylogenetic tree based on the maximum likelihood method in the result (Figure 3D).

#### GeneWise

Furthermore, GeneWise (39) is used to conduct comparisons of protein sequences or DNA sequences, in which information related to the cutting site is considered. The greatest advantage of GeneWise is that it can link multiple exons of the gene in order to obtain an overall comparison of the gene. In the case of default parameters, the protein sequence (Query) and DNA sequence (Target) are input. In the output result, 259.55 is the score of the GeneWise alignment; the higher the score is, the higher the quality will be (Figure 3E).

#### LASTZ

In order to align the genomic or nucleotide sequences more intuitively and efficiently, OGDA integrates the Large-Scale Genome Alignment Tool (LASTZ) (40), which is a fast and powerful tool for the pairwise alignment of genomic DNA sequences. LASTZ was designed with large-scale genomic analysis in mind for the efficient alignment of chromosomal or genomic sequences millions of nucleotides in length. It identifies orthologous regions between genomic sequences on a massive scale using a methodology that ignores the coding-region bias. When using this function, users input the genome sequences of the two species to be analyzed and perform the run operation to obtain the synteny analysis results of the two genomes (Figure 3F).

### Taxonomy tree

A large number of algal taxa are included in OGDA, and the classification system is still developing. Therefore, the ‘Taxonomy tree’ module in OGDA sorts all algae according to the classification and naming information of global alga in AlgaeBase. ‘Taxonomy tree’ is updated in real time according to the most recent research.

## Discussion

As one of the most complicated organisms in the biosphere, there are still a large number of algal species that have not been identified or classified. Therefore, an algae-related database was urgently required. In comparison with ChloroplastDB (33) and CpGDB (34), OGDA pays more attention to the group of algae, and this database is the first algal genome database, integrating a large amount of algal genome data from different sources to provide abundant algae genome resources. Users can directly select the species of interest and obtain relevant information such as species location, genomic sequences and genomic maps through online browsing and searching. Users can also analyze their own data online through the website, such as sequence search and comparison, format conversion and multiple alignments.

The organelle genomes of terrestrial plants and metazoans contain a large amount of genetic variation information and their characteristics are relatively stable. Thus, they can be used as a marker to reflect biological origins, evolution, classification, etc., and are better able to clarify the phylogenetic relationship of various groups of organisms (11, 14, 18, 25). Moreover, due to the special inheritance pattern of organelle genomes, studies on the structure and function of organelles have revealed important biological phenomena such as rice fertility (41) and human diseases (42). However, there is still a lack of research on this aspect for algae. OGDA provides a new tool for further studies on the genome structure and function of eukaryotic algae organelles. More importantly, OGDA can provide a theoretical basis and support for the breeding, germplasm resource identification and protection of macroalgae.

With the development of genomic sequencing technology, the annual growth rate of genomic databases such as NCBI has reached 30%. The application of genomic data has since penetrated almost all aspects of life science research. Since 2005, high-throughput sequencing technology has been widely used in algae genome sequencing, transcriptome sequencing and epigenetic research and has opened up a new avenue for algae genomics research (12). A typical model organism is *Chlamydomonas reinhardtii*, which uses genomic data to study the origin of photosynthesis and flagella (43, 44). Recently, with increasing research on algae as a buffering biological system for global warming and a supplementary source of energy and food, genomic data of more algae species have been determined. This includes algae that grow in extreme environments, *Cyanidioschyzon merolae* (45), and genomic sketches of the marine diatom *Thalassiosira pseudonana* (46). With the further development of high-throughput sequencing methods, reports of algae genomes have also increased rapidly. Compared with the nuclear genome, the organelle genome contains precious genetic information. For example, more conserved genes in the organelle can more clearly reflect the evolutionary history, and sequences with a higher evolution rate encoded by the organelle can be used to study the systemic relationship at the species level (47). The small organelle genome and closed loop feature mean that the de novo sequencing is relatively simple and easy to splice, and subsequent analysis can facilitate the operation, resulting in faster accumulation of organelle genome data, which leads to challenges related to genome ‘big data’ (48). Therefore, the development of a professional database will help more scientists skillfully use genomic data. However, it should be noted that the gene database in this area is still limited, and there are still some potential errors in the public database. OGDA provides a user-friendly tool for organellar genome research of eukaryotic algae, including data submission, gene annotation, gene synteny analysis, and phylogenetic analysis, which is expected to attract more scientists to algal genome research in future.

In conclusion, OGDA is a comprehensive platform for algae-related biological information that provides genomic data for a variety of algae and a series of genome analysis tools. At present, the algal data of OGDA predominantly originate from NCBI. In future, we will integrate algal genome data from more comprehensive databases and sequence more algae species in our laboratory (MOGBL). All genomic information in OGDA will be shared online. Subsequently, we will also integrate more biological information analysis tools, making OGDA a more complete platform for algae information sharing.

## Supplementary Material

baaa097_Supp

## Availability

The OGDA database can be accessed through the web server at http://ogda.ytu.edu.cn/.
