# Supplementary material for: OGDA: a comprehensive organelle genome database for algae
Source: Database (Oxford). 2020 Nov 28;2020:baaa097. doi: 10.1093/database/baaa097 (PMC7698662; doi:10.1093/database/baaa097)
Supplement: baaa097_Supp [file baaa097_supp.zip › supplementary_data_v2.docx]

# Table S 1. The chloroplast genome data from the public database

| Species | accession  number | Raw data source |
| --- | --- | --- |
| *Nitzschia palea* | MH113811.1 | <https://www.ncbi.nlm.nih.gov/nuccore/MH113811.1> |
| *Seminavis robusta* strain D6 | MH356727.1 | <https://www.ncbi.nlm.nih.gov/nuccore/MH356727.1> |
| *Odontella sinensis* | NC_001713.1  Z67753.1 | <https://www.ncbi.nlm.nih.gov/nuccore/NC_001713.1>  <https://www.ncbi.nlm.nih.gov/nuccore/Z67753.1> |
| *Leptocylindrus danicus* | NC_024084.1  KC509524.1 | <https://www.ncbi.nlm.nih.gov/nuccore/NC_024084.1>  <https://www.ncbi.nlm.nih.gov/nuccore/KC509524.1> |
| *Eunotia naegelii* | NC_024928.1  KF733443.1 | <https://www.ncbi.nlm.nih.gov/nuccore/NC_024928.1>  <https://www.ncbi.nlm.nih.gov/nuccore/KF733443.1> |
| *Thalassiosira oceanica* CCMP1005 | NC_014808.1  GU323224.1 | <https://www.ncbi.nlm.nih.gov/nuccore/NC_014808.1>  <https://www.ncbi.nlm.nih.gov/nuccore/GU323224.1> |
| *Toxarium undulatum* strain ECT3802 | NC_031425.1  KX619437.1 | <https://www.ncbi.nlm.nih.gov/nuccore/NC_031425.1>  <https://www.ncbi.nlm.nih.gov/nuccore/KX619437.1> |
| *Fistulifera solaris* | NC_015403.1  AP011960.1 | <https://www.ncbi.nlm.nih.gov/nuccore/NC_015403.1>  <https://www.ncbi.nlm.nih.gov/nuccore/AP011960.1> |
| *Roundia cardiophora* | NC_025312.1  KJ958483.1 | <https://www.ncbi.nlm.nih.gov/nuccore/NC_025312.1>  <https://www.ncbi.nlm.nih.gov/nuccore/KJ958483.1> |
| *Cyclotella* sp. L04_2 | KJ958480.1 | <https://www.ncbi.nlm.nih.gov/nuccore/KJ958480.1> |
| *Chaetoceros* *simplex* | NC_025310.1  KJ958479.1 | https://www.ncbi.nlm.nih.gov/nuccore/NC_025310.1  <https://www.ncbi.nlm.nih.gov/nuccore/KJ958479.1> |
| *Cerataulina* *daemon* | NC_025313.1  KJ958484.1 | https://www.ncbi.nlm.nih.gov/nuccore/NC_025313.1  <https://www.ncbi.nlm.nih.gov/nuccore/KJ958484.1> |
| *Rhizosolenia imbricata* | NC_025311.1  KJ958482.1 | https://www.ncbi.nlm.nih.gov/nuccore/NC_025311.1  <https://www.ncbi.nlm.nih.gov/nuccore/KJ958482.1> |
| *Thalassiosira weissflogii* | NC_025314.1  KJ958485.1 | https://www.ncbi.nlm.nih.gov/nuccore/NC_025314.1  <https://www.ncbi.nlm.nih.gov/nuccore/KJ958485.1> |
| *Cyclotella* sp. WC03_2 | KJ958481.1 | <https://www.ncbi.nlm.nih.gov/nuccore/KJ958481.1> |
| *Thalassiosira pseudonana* | NC_008589.1  EF067921.1 | https://www.ncbi.nlm.nih.gov/nuccore/NC_008589.1  <https://www.ncbi.nlm.nih.gov/nuccore/EF067921.1> |
| *Phaeodactylum tricornutum* | NC_008588.1  EF067920.1 | https://www.ncbi.nlm.nih.gov/nuccore/NC_008588.1  <https://www.ncbi.nlm.nih.gov/nuccore/EF067920.1> |
| *Biddulphia tridens* | NC_038007.1  MG755806.1 | https://www.ncbi.nlm.nih.gov/nuccore/NC_038007.1  <https://www.ncbi.nlm.nih.gov/nuccore/MG755806.1> |
| *Biddulphia biddulphiana* | NC_038006.1  MG755805.1 | https://www.ncbi.nlm.nih.gov/nuccore/NC_038006.1  <https://www.ncbi.nlm.nih.gov/nuccore/MG755805.1> |
| *Attheya longicornis* | NC_037999.1  MG755798.1 | https://www.ncbi.nlm.nih.gov/nuccore/NC_037999.1  <https://www.ncbi.nlm.nih.gov/nuccore/MG755798.1> |
| *Cyclotella pseudostelligera* | NC_038005.1  MG755804.1 | https://www.ncbi.nlm.nih.gov/nuccore/NC_038005.1  <https://www.ncbi.nlm.nih.gov/nuccore/MG755804.1> |
| *Acanthoceras zachariasii* | NC_038009.1  MG755808.1 | https://www.ncbi.nlm.nih.gov/nuccore/NC_038009.1  <https://www.ncbi.nlm.nih.gov/nuccore/MG755808.1> |
| *Lithodesmium undulatum* | NC_024085.1  KC509525.1 | https://www.ncbi.nlm.nih.gov/nuccore/NC_024085.1  <https://www.ncbi.nlm.nih.gov/nuccore/KC509525.1> |
| *Plagiogrammopsis vanheurckii* | NC_037997.1  MG755794.1 | https://www.ncbi.nlm.nih.gov/nuccore/NC_037997.1  <https://www.ncbi.nlm.nih.gov/nuccore/MG755794.1> |
| *Eunotogramma* sp. | MG755797.1 | <https://www.ncbi.nlm.nih.gov/nuccore/MG755797.1> |
| *Pseudo-nitzschia multiseries* | NC_027721.1  KR709240.1 | https://www.ncbi.nlm.nih.gov/nuccore/NC_027721.1  <https://www.ncbi.nlm.nih.gov/nuccore/KR709240.1> |
| *Nitzschia palea* NIES-2729 | AP018511.1 | <https://www.ncbi.nlm.nih.gov/nuccore/AP018511.1> |
| *Cylindrotheca closterium* | NC_024082.1  KC509522.1 | https://www.ncbi.nlm.nih.gov/nuccore/NC_024082.1  <https://www.ncbi.nlm.nih.gov/nuccore/KC509522.1> |
| *Psammoneis obaidii* | NC_038004.1  MG755803.1 | https://www.ncbi.nlm.nih.gov/nuccore/NC_038004.1  <https://www.ncbi.nlm.nih.gov/nuccore/MG755803.1> |
| *Plagiogramma staurophorum* | NC_037995.1  MG755792.1 | https://www.ncbi.nlm.nih.gov/nuccore/NC_037995.1  <https://www.ncbi.nlm.nih.gov/nuccore/MG755792.1> |
| *Gomphoneis minuta* var. *cassieae* | KY499654.1 | <https://www.ncbi.nlm.nih.gov/nuccore/KY499654.1> |
| *Synedra acus* | NC_016731.1  JQ088178.1 | https://www.ncbi.nlm.nih.gov/nuccore/NC_016731.1  <https://www.ncbi.nlm.nih.gov/nuccore/JQ088178.1> |
| *Astrosyne radiata* | NC_038008.1  MG755807.1 | https://www.ncbi.nlm.nih.gov/nuccore/NC_038008.1  <https://www.ncbi.nlm.nih.gov/nuccore/MG755807.1> |
| *Didymosphenia geminata* | NC_024083.1  KC509523.1 | https://www.ncbi.nlm.nih.gov/nuccore/NC_024083.1  <https://www.ncbi.nlm.nih.gov/nuccore/KC509523.1> |
| *Licmophora* sp. | MG755795.1 | <https://www.ncbi.nlm.nih.gov/nuccore/MG755795.1> |
| *Entomoneis* sp. | NC_038001.1  MG755800.1 | https://www.ncbi.nlm.nih.gov/nuccore/NC_038001.1  <https://www.ncbi.nlm.nih.gov/nuccore/MG755800.1> |
| *Asterionella formosa* | NC_024079.1  KC509519.1 | https://www.ncbi.nlm.nih.gov/nuccore/NC_024079.1  <https://www.ncbi.nlm.nih.gov/nuccore/KC509519.1> |
| *Asterionellopsis glacialis* | NC_024080.1  KC509520.1 | https://www.ncbi.nlm.nih.gov/nuccore/NC_024080.1  <https://www.ncbi.nlm.nih.gov/nuccore/KC509520.1> |
| *Rhizosolenia fallax* | NC_038003.1  MG755802.1 | https://www.ncbi.nlm.nih.gov/nuccore/NC_038003.1  <https://www.ncbi.nlm.nih.gov/nuccore/MG755802.1> |
| *Rhizosolenia setigera* | NC_037996.1  MG755793.1 | https://www.ncbi.nlm.nih.gov/nuccore/NC_037996.1  <https://www.ncbi.nlm.nih.gov/nuccore/MG755793.1> |
| *Guinardia striata* | NC_037998.1  MG755796.1 | https://www.ncbi.nlm.nih.gov/nuccore/NC_037998.1  <https://www.ncbi.nlm.nih.gov/nuccore/MG755796.1> |
| *Proboscia* sp. | MG755791.1 | <https://www.ncbi.nlm.nih.gov/nuccore/MG755791.1> |
| *Coscinodiscus radiatus* | NC_024081.1  KC509521.1 | https://www.ncbi.nlm.nih.gov/nuccore/NC_024081.1  <https://www.ncbi.nlm.nih.gov/nuccore/KC509521.1> |
| *Actinocyclus subtilis* | NC_038000.1  MG755799.1 | https://www.ncbi.nlm.nih.gov/nuccore/NC_038000.1  <https://www.ncbi.nlm.nih.gov/nuccore/MG755799.1> |
| *Triceratium dubium* | NC_038002.1  MG755801.1 | https://www.ncbi.nlm.nih.gov/nuccore/NC_038002.1  <https://www.ncbi.nlm.nih.gov/nuccore/MG755801.1> |
| *Lotharella* sp. CCMP622 | KF438023.1 | <https://www.ncbi.nlm.nih.gov/nuccore/KF438023.1> |
| *Staurastrum punctulatum* | NC_008116.1  AY958085.1 | https://www.ncbi.nlm.nih.gov/nuccore/NC_008116.1  AY958085.1 |
| *Zygnema circumcarinatum* | NC_008117.1  AY958086.1 | https://www.ncbi.nlm.nih.gov/nuccore/NC_008117.1  <https://www.ncbi.nlm.nih.gov/nuccore/AY958086.1> |
| *Chlorokybus atmophyticus* | NC_008822.1  DQ422812.2 | https://www.ncbi.nlm.nih.gov/nuccore/NC_008822.1  <https://www.ncbi.nlm.nih.gov/nuccore/DQ422812.2> |
| *Chara vulgaris* | NC_008097.1  DQ229107.1 | https://www.ncbi.nlm.nih.gov/nuccore/NC_008097.1  <https://www.ncbi.nlm.nih.gov/nuccore/DQ229107.1> |
| *Chaetosphaeridium globosum* | NC_004115.1  AF494278.1 | https://www.ncbi.nlm.nih.gov/nuccore/NC_004115.1  <https://www.ncbi.nlm.nih.gov/nuccore/AF494278.1> |
| *Entransia fimbriata* culture-collection UTEX:LB 2353 | NC_030313.1  KU646490.1 | https://www.ncbi.nlm.nih.gov/nuccore/NC_030313.1  <https://www.ncbi.nlm.nih.gov/nuccore/KU646490.1> |
| *Netrium digitus* culture-collection UTEXLB 561 | NC_030356.1  KU646491.1 | https://www.ncbi.nlm.nih.gov/nuccore/NC_030356.1  <https://www.ncbi.nlm.nih.gov/nuccore/KU646491.1> |
| *Cosmarium botrytis* culture-collection UTEX175 | NC_030357.1  KU646492.1 | https://www.ncbi.nlm.nih.gov/nuccore/NC_030357.1  <https://www.ncbi.nlm.nih.gov/nuccore/KU646492.1> |
| *Spirogyra maxima* culture-collection UTEX:LB 2495 | NC_030355.1  KU646489.1 | https://www.ncbi.nlm.nih.gov/nuccore/NC_030355.1  <https://www.ncbi.nlm.nih.gov/nuccore/KU646489.1> |
| *Closterium baillyanum* culture-collection SAG50.89 | NC_030314.1  KU646494.1 | https://www.ncbi.nlm.nih.gov/nuccore/NC_030314.1  <https://www.ncbi.nlm.nih.gov/nuccore/KU646494.1> |
| *Roya obtusa* culture-collection SAG:168.80 | NC_030315.1  KU646496.1 | https://www.ncbi.nlm.nih.gov/nuccore/NC_030315.1  <https://www.ncbi.nlm.nih.gov/nuccore/KU646496.1> |
| *Cylindrocystis brebissonii* culture-collection SAG:615-1 | NC_030359.1  KU646495.1 | https://www.ncbi.nlm.nih.gov/nuccore/NC_030359.1  <https://www.ncbi.nlm.nih.gov/nuccore/KU646495.1> |
| *Coleochaete scutata* culture-collection SAG:110.80M | NC_030358.1  KU646493.1 | https://www.ncbi.nlm.nih.gov/nuccore/NC_030358.1  <https://www.ncbi.nlm.nih.gov/nuccore/KU646493.1> |
| *Mesostigma viride* | NC_002186.1 | <https://www.ncbi.nlm.nih.gov/nuccore/NC_002186.1> |
| *Klebsormidium flaccidum* culture-collection SAG:121.80 | NC_024167.1  KJ461680.1 | https://www.ncbi.nlm.nih.gov/nuccore/NC_024167.1  <https://www.ncbi.nlm.nih.gov/nuccore/KJ461680.1> |
| *Mesotaenium endlicherianum* culture-collection SAG12.97 | NC_024169.1  KJ461682.1 | https://www.ncbi.nlm.nih.gov/nuccore/NC_024169.1  <https://www.ncbi.nlm.nih.gov/nuccore/KJ461682.1> |
| *Roya anglica* culture-collection ACOI:799 | NC_024168.1  KJ461681.1 | https://www.ncbi.nlm.nih.gov/nuccore/NC_024168.1  <https://www.ncbi.nlm.nih.gov/nuccore/KJ461681.1> |
| *Nitella hyalina* | KX306884.1 | <https://www.ncbi.nlm.nih.gov/nuccore/KX306884.1> |
| *Chloroparvula pacifica* culture RCC:4656 | NC_042489.1  MK085995.1 | https://www.ncbi.nlm.nih.gov/nuccore/NC_042489.1  <https://www.ncbi.nlm.nih.gov/nuccore/MK085995.1> |
| *Chloroparvula japonica* culture RCC:2339 | NC_042487.1  MK085993.1 | https://www.ncbi.nlm.nih.gov/nuccore/NC_042487.1  <https://www.ncbi.nlm.nih.gov/nuccore/MK085993.1> |
| *Chloroparvula* sp. RCC4572 culture RCC:4572 | MK085996.1 | <https://www.ncbi.nlm.nih.gov/nuccore/MK085996.1> |
| *Chloroparvula* sp. RCC999 culture RCC:999 | MK085990.1 | <https://www.ncbi.nlm.nih.gov/nuccore/MK085990.1> |
| *Chloroparvula* sp. RCC696 culture RCC696 | MK085988.1 | <https://www.ncbi.nlm.nih.gov/nuccore/MK085988.1> |
| *Chloropicon* *maureeniae* culture RCC:3374 | NC_042488.1  MK085994.1 | https://www.ncbi.nlm.nih.gov/nuccore/NC_042488.1  <https://www.ncbi.nlm.nih.gov/nuccore/MK085994.1> |
| *Chloropicon* *roscoffensis* culture RCC:1871 | NC_042486.1  MK085991.1 | https://www.ncbi.nlm.nih.gov/nuccore/NC_042486.1  <https://www.ncbi.nlm.nih.gov/nuccore/MK085991.1> |
| *Chloropicon* *mariensis* culture RCC998 | NC_042485.1  MK085989.1 | https://www.ncbi.nlm.nih.gov/nuccore/NC_042485.1  <https://www.ncbi.nlm.nih.gov/nuccore/MK085989.1> |
| *Chloropicon* *laureae* culture RCC:856 | NC_042484.1  MK085987.1 | https://www.ncbi.nlm.nih.gov/nuccore/NC_042484.1  <https://www.ncbi.nlm.nih.gov/nuccore/MK085987.1> |
| *Chloropicon* *sieburthii* culture RCC:287 | NC_042483.1  MK085986.1 | https://www.ncbi.nlm.nih.gov/nuccore/NC_042483.1  <https://www.ncbi.nlm.nih.gov/nuccore/MK085986.1> |
| *Chloropicon* sp. RCC4434 culture RCC:4434 | MK085997.1 | <https://www.ncbi.nlm.nih.gov/nuccore/MK085997.1> |
| *Chloropicon* *roscoffensis* culture RCC:2335 | MK085992.1 | <https://www.ncbi.nlm.nih.gov/nuccore/MK085992.1> |
| *Chlamydomonas* *leiostraca* strain SAG 11-49 | NC_032109.1  KX828176.1 | https://www.ncbi.nlm.nih.gov/nuccore/NC_032109.1  <https://www.ncbi.nlm.nih.gov/nuccore/KX828176.1> |
| *Floydiella* *terrestris* | NC_014346.1 | <https://www.ncbi.nlm.nih.gov/nuccore/NC_014346.1> |
| *Dunaliella* *salina* | NC_016732.1 | <https://www.ncbi.nlm.nih.gov/nuccore/NC_016732.1> |
| *Dunaliella* *salina* strain CCAP 19/18 | GQ250046.1 | <https://www.ncbi.nlm.nih.gov/nuccore/GQ250046.1> |
| *Lobosphaera incisa* | KM821265.1 | <https://www.ncbi.nlm.nih.gov/nuccore/KM821265.1> |
| *Helicosporidium* sp. ex *Simulium jonesii* | NC_008100.1  DQ398104.1 | https://www.ncbi.nlm.nih.gov/nuccore/NC_008100.1  <https://www.ncbi.nlm.nih.gov/nuccore/DQ398104.1> |
| *Acutodesmus obliquus* strain UTEX 393 | NC_008101.1 | <https://www.ncbi.nlm.nih.gov/nuccore/NC_008101.1> |
| *Dunaliella salina strain* SQ | KX530454.1 | <https://www.ncbi.nlm.nih.gov/nuccore/KX530454.1> |
| *Oltmannsiellopsis viridis* | NC_008099.1  DQ291132.1 | https://www.ncbi.nlm.nih.gov/nuccore/NC_008099.1  <https://www.ncbi.nlm.nih.gov/nuccore/DQ291132.1> |
| *Nephroselmis olivacea* | NC_000927.1 | <https://www.ncbi.nlm.nih.gov/nuccore/NC_000927.1> |
| *Nephroselmis olivacea* chloroplast DNA | AF137379.1 | <https://www.ncbi.nlm.nih.gov/nuccore/AF137379.1> |
| *Ulva* sp. UNA00071828 | KP720616.1 | <https://www.ncbi.nlm.nih.gov/nuccore/KP720616.1> |
| *Ostreococcus* *tauri* | NC_008289.1  CR954199.2 | https://www.ncbi.nlm.nih.gov/nuccore/NC_008289.1  <https://www.ncbi.nlm.nih.gov/nuccore/CR954199.2> |
| *Pyramimonas parkeae* | NC_012099.1  FJ493499.1 | https://www.ncbi.nlm.nih.gov/nuccore/NC_012099.1  <https://www.ncbi.nlm.nih.gov/nuccore/FJ493499.1> |
| *Pycnococcus provasolii* | NC_012097.1  FJ493498.1 | <https://www.ncbi.nlm.nih.gov/nuccore/NC_012097.1>  <https://www.ncbi.nlm.nih.gov/nuccore/FJ493498.1> |
| *Pedinomonas minor* | NC_016733.1 | <https://www.ncbi.nlm.nih.gov/nuccore/NC_016733.1> |
| *Pedinomonas minor* culture-collection UTEX:LB 1350 | FJ968740.1 | <https://www.ncbi.nlm.nih.gov/nuccore/FJ968740.1> |
| *Parachlorella kessleri* culture-collection SAG:211-11g | FJ968741.1 | <https://www.ncbi.nlm.nih.gov/nuccore/FJ968741.1> |
| *Parachlorella kessleri* | NC_012978.1 | <https://www.ncbi.nlm.nih.gov/nuccore/NC_012978.1> |
| *Bryopsis plumosa* strain West4718 | NC_026795.1  LN810504.1 | https://www.ncbi.nlm.nih.gov/nuccore/NC_026795.1  <https://www.ncbi.nlm.nih.gov/nuccore/LN810504.1> |
| *Tydemania expeditionis* strain FL1151 | NC_026796.1  LN810505.1 | https://www.ncbi.nlm.nih.gov/nuccore/NC_026796.1  <https://www.ncbi.nlm.nih.gov/nuccore/LN810505.1> |
| *Pseudendoclonium akinetum* | NC_008114.1  AY835431.1 | https://www.ncbi.nlm.nih.gov/nuccore/NC_008114.1  <https://www.ncbi.nlm.nih.gov/nuccore/AY835431.1> |
| *Leptosira terrestris* UTEX 333 | EF506945.1 | <https://www.ncbi.nlm.nih.gov/nuccore/EF506945.1> |
| *Schizomeris leibleinii* | NC_015645.1 | <https://www.ncbi.nlm.nih.gov/nuccore/NC_015645.1> |
| *Schizomeris leibleinii* culture-collection UTEX:LB 1228 | HQ700713.1 | <https://www.ncbi.nlm.nih.gov/nuccore/HQ700713.1> |
| *Bryopsis hypnoides* | NC_013359.1  GQ892829.1 | https://www.ncbi.nlm.nih.gov/nuccore/NC_013359.1  <https://www.ncbi.nlm.nih.gov/nuccore/GQ892829.1> |
| *Nephroselmis astigmatica* culture-collection NIES:252 | NC_024829.1  KJ746600.1 | https://www.ncbi.nlm.nih.gov/nuccore/NC_024829.1  <https://www.ncbi.nlm.nih.gov/nuccore/KJ746600.1> |
| *Prasinoderma coloniale* culture-collection CCMP:1220 | NC_024817.1  KJ746598.1 | https://www.ncbi.nlm.nih.gov/nuccore/NC_024817.1  <https://www.ncbi.nlm.nih.gov/nuccore/KJ746598.1> |
| *Prasinococcus* sp. CCMP1194 | KJ746597.1 | <https://www.ncbi.nlm.nih.gov/nuccore/KJ746597.1> |
| *Picocystis* *salinarum* culture-collection CCMP:1897 | NC_024828.1  KJ746599.1 | https://www.ncbi.nlm.nih.gov/nuccore/NC_024828.1  <https://www.ncbi.nlm.nih.gov/nuccore/KJ746599.1> |
| *Prasinophyceae* sp. CCMP1205 | KJ746601.1 | <https://www.ncbi.nlm.nih.gov/nuccore/KJ746601.1> |
| *Prasinophyceae* sp. MBIC10622 | KJ746602.1 | <https://www.ncbi.nlm.nih.gov/nuccore/KJ746602.1> |
| *Caulerpa* *verticillata* voucher HV05119 | NC_039523.1  MH591106.1 | https://www.ncbi.nlm.nih.gov/nuccore/NC_039523.1  <https://www.ncbi.nlm.nih.gov/nuccore/MH591106.1> |
| *Codium* *simulans* | NC_032043.1  KT946603.1 | https://www.ncbi.nlm.nih.gov/nuccore/NC_032043.1  <https://www.ncbi.nlm.nih.gov/nuccore/KT946603.1> |
| *Codium* *arabicum* voucher HV03886 | NC_039524.1  MH591107.1 | https://www.ncbi.nlm.nih.gov/nuccore/NC_039524.1  <https://www.ncbi.nlm.nih.gov/nuccore/MH591107.1> |
| *Pedobesia* *claviformis* voucher HV04005 | NC_039766.1  MH591108.1 | https://www.ncbi.nlm.nih.gov/nuccore/NC_039766.1  <https://www.ncbi.nlm.nih.gov/nuccore/MH591108.1> |
| *Udotea* *flabellum* voucher HV02674 | NC_039528.1  MH591112.1 | https://www.ncbi.nlm.nih.gov/nuccore/NC_039528.1  <https://www.ncbi.nlm.nih.gov/nuccore/MH591112.1> |
| *Udotea* *argentea* voucher HV04251 | NC_039527.1  MH591111.1 | https://www.ncbi.nlm.nih.gov/nuccore/NC_039527.1  <https://www.ncbi.nlm.nih.gov/nuccore/MH591111.1> |
| *Udotea* sp. TZ0819 | MH591113.1 | <https://www.ncbi.nlm.nih.gov/nuccore/MH591113.1> |
| *Callipsygma* *wilsonis* voucher HV03983 | NC_039522.1  MH591105.1 | https://www.ncbi.nlm.nih.gov/nuccore/NC_039522.1  <https://www.ncbi.nlm.nih.gov/nuccore/MH591105.1> |
| *Boodleopsis* sp. H.0758 | NC_039521.1  MH591104.1 | https://www.ncbi.nlm.nih.gov/nuccore/NC_039521.1  <https://www.ncbi.nlm.nih.gov/nuccore/MH591104.1> |
| *Boodleopsis* sp. FL1161 | MH591102.1 | <https://www.ncbi.nlm.nih.gov/nuccore/MH591102.1> |
| *Boodleopsis* *pusilla* voucher C.0205 | NC_039520.1  MH591103.1 | https://www.ncbi.nlm.nih.gov/nuccore/NC_039520.1  <https://www.ncbi.nlm.nih.gov/nuccore/MH591103.1> |
| *Pseudocodium* *devriesii* voucher DH02.0274 | NC_039525.1 | <https://www.ncbi.nlm.nih.gov/nuccore/NC_039525.1> |
| *Rhipiliopsis* *peltata* voucher JFC00414 | NC_039526.1  MH591110.1 | https://www.ncbi.nlm.nih.gov/nuccore/NC_039526.1  <https://www.ncbi.nlm.nih.gov/nuccore/MH591110.1> |
| *Lambia* *antarctica* | NC_032284.1  KU059765.1 | https://www.ncbi.nlm.nih.gov/nuccore/NC_032284.1  <https://www.ncbi.nlm.nih.gov/nuccore/KU059765.1> |
| *Pediastrum* *duplex* voucher strain AL0403MN | MF276979.1 | <https://www.ncbi.nlm.nih.gov/nuccore/MF276979.1> |
| *Hydrodictyon* *reticulatum* voucher HAM0289 | NC_034655.1  KY114065.1 | https://www.ncbi.nlm.nih.gov/nuccore/NC_034655.1  <https://www.ncbi.nlm.nih.gov/nuccore/KY114065.1> |
| *Chlorella* sp. ArM0029B | KF554427.1 | <https://www.ncbi.nlm.nih.gov/nuccore/KF554427.1> |
| *Ostreobium* sp. HV05042 | KY509314.1 | <https://www.ncbi.nlm.nih.gov/nuccore/KY509314.1> |
| *Pseudopediastrum* *boryanum* voucher ML0410MN | MF276983.1 | <https://www.ncbi.nlm.nih.gov/nuccore/MF276983.1> |
| *Chlorotetraedron* *incus* strain SAG 43.81 | NC_029673.1  KT199252.1 | https://www.ncbi.nlm.nih.gov/nuccore/NC_029673.1  <https://www.ncbi.nlm.nih.gov/nuccore/KT199252.1> |
| *Ostreococcus tauri* | KC990831.1 | <https://www.ncbi.nlm.nih.gov/nuccore/KC990831.1> |
| *Ostreococcus* *tauri* isolate RCC1108 | KF285522.1 | <https://www.ncbi.nlm.nih.gov/nuccore/KF285522.1> |
| *Ostreococcus* *tauri* isolate RCC1110 | KF285523.1 | <https://www.ncbi.nlm.nih.gov/nuccore/KF285523.1> |
| *Ostreococcus* *tauri* isolate RCC1112 | KF285524.1 | <https://www.ncbi.nlm.nih.gov/nuccore/KF285524.1> |
| *Ostreococcus* *tauri* isolate RCC1114 | KF285525.1 | <https://www.ncbi.nlm.nih.gov/nuccore/KF285525.1> |
| *Ostreococcus* *tauri* isolate RCC1115 | KF285526.1 | <https://www.ncbi.nlm.nih.gov/nuccore/KF285526.1> |
| *Ostreococcus* *tauri* isolate RCC1116 | KF285527.1 | <https://www.ncbi.nlm.nih.gov/nuccore/KF285527.1> |
| *Ostreococcus* *tauri* isolate RCC1558 | KF285531.1 | <https://www.ncbi.nlm.nih.gov/nuccore/KF285531.1> |
| *Ostreococcus* *tauri* isolate RCC1559 | KF285532.1 | <https://www.ncbi.nlm.nih.gov/nuccore/KF285532.1> |
| *Ostreococcus* *tauri* isolate RCC1561 | KF285533.1 | <https://www.ncbi.nlm.nih.gov/nuccore/KF285533.1> |
| *Ostreococcus* *tauri* isolate RCC1117 | KF285528.1 | <https://www.ncbi.nlm.nih.gov/nuccore/KF285528.1> |
| *Ostreococcus* *tauri* isolate RCC1118 | KF285529.1 | <https://www.ncbi.nlm.nih.gov/nuccore/KF285529.1> |
| *Ostreococcus* *tauri* isolate RCC1123 | KF285530.1 | <https://www.ncbi.nlm.nih.gov/nuccore/KF285530.1> |
| *Uronema* sp. CCAP 334/1 culture UTEX:CCAP 334/1 | MG778533.1 | <https://www.ncbi.nlm.nih.gov/nuccore/MG778533.1> |
| *Chlamydomonas* *reinhardtii* strain CC-503 cw92 mt+ | FJ423446.1 | <https://www.ncbi.nlm.nih.gov/nuccore/FJ423446.1> |
| *Chlamydomonas* *reinhardtii* | NC_005353.1 | <https://www.ncbi.nlm.nih.gov/nuccore/NC_005353.1> |
| *Picochlorum* sp. 'soloecismus' strain DOE 101 | MG552671.1 | <https://www.ncbi.nlm.nih.gov/nuccore/MG552671.1> |
| *Chlorella heliozoae* | NC_036805.1  KY629616.1 | <https://www.ncbi.nlm.nih.gov/nuccore/NC_036805.1>  <https://www.ncbi.nlm.nih.gov/nuccore/KY629616.1> |
| *Micractinium conductrix* | NC_036806.1  KY629620.1 | https://www.ncbi.nlm.nih.gov/nuccore/NC_036806.1  <https://www.ncbi.nlm.nih.gov/nuccore/KY629620.1> |
| *Prototheca* *cutis* chloroplast DNA | NC_037480.1  AP018373.1 | https://www.ncbi.nlm.nih.gov/nuccore/NC_037480.1  <https://www.ncbi.nlm.nih.gov/nuccore/AP018373.1> |
| *Prototheca* *stagnorum* chloroplast DNA, | NC_037479.1  AP018372.1 | https://www.ncbi.nlm.nih.gov/nuccore/NC_037479.1  <https://www.ncbi.nlm.nih.gov/nuccore/AP018372.1> |
| *Gloeotilopsis sarcinoidea* | KX306821.1 | <https://www.ncbi.nlm.nih.gov/nuccore/KX306821.1> |
| *Gloeotilopsis* *planctonica* culture-collection SAG:29.93 | KX306824.1 | <https://www.ncbi.nlm.nih.gov/nuccore/KX306824.1> |
| *Gonium* *pectorale* | NC_020438.1 | <https://www.ncbi.nlm.nih.gov/nuccore/NC_020438.1> |
| *Ulva* *ohnoi* chloroplast DNA | AP018696.1 | <https://www.ncbi.nlm.nih.gov/nuccore/AP018696.1> |
| *Bryopsis* sp. HV04063 | NC_037363.1  KY819063.1 | https://www.ncbi.nlm.nih.gov/nuccore/NC_037363.1  <https://www.ncbi.nlm.nih.gov/nuccore/KY819063.1> |
| *Caulerpa manorensis* | NC_037367.1  KY819068.1 | https://www.ncbi.nlm.nih.gov/nuccore/NC_037367.1  <https://www.ncbi.nlm.nih.gov/nuccore/KY819068.1> |
| *Chlorodesmis fastigiata* | NC_037364.1  KY819064.1 | https://www.ncbi.nlm.nih.gov/nuccore/NC_037364.1  <https://www.ncbi.nlm.nih.gov/nuccore/KY819064.1> |
| *Rhipilia penicilloides* | NC_037365.1  KY819065.1 | NC_037365.1  <https://www.ncbi.nlm.nih.gov/nuccore/KY819065.1> |
| *Prototheca* *zopfii* strain SAG 2063 | NC_037450.1  MF197535.1 | https://www.ncbi.nlm.nih.gov/nuccore/NC_037450.1  <https://www.ncbi.nlm.nih.gov/nuccore/MF197535.1> |
| *Prototheca* *zopfii* strain SAG 2021 | MF197536.1 | <https://www.ncbi.nlm.nih.gov/nuccore/MF197536.1> |
| *Ulva* *lactuca* voucher L0054996 | NC_042255.1  MH730972.1 | https://www.ncbi.nlm.nih.gov/nuccore/NC_042255.1  <https://www.ncbi.nlm.nih.gov/nuccore/MH730972.1> |
| *Capsosiphon* *fulvescens* | NC_039920.1  MG727869.1 | https://www.ncbi.nlm.nih.gov/nuccore/NC_039920.1  <https://www.ncbi.nlm.nih.gov/nuccore/MG727869.1> |
| *Yamagishiella* *unicocca* strain NIES-3982 | NC_039754.1  MH285950.1 | https://www.ncbi.nlm.nih.gov/nuccore/NC_039754.1  <https://www.ncbi.nlm.nih.gov/nuccore/MH285950.1> |
| *Volvox* *africanus* strain 2013-0703-VO4 | NC_039755.1  MH285951.1 | https://www.ncbi.nlm.nih.gov/nuccore/NC_039755.1  <https://www.ncbi.nlm.nih.gov/nuccore/MH285951.1> |
| *Eudorina* sp. NIES-3984 | MH267732.1 | <https://www.ncbi.nlm.nih.gov/nuccore/MH267732.1> |
| *Caulerpa* *cliftonii* voucher HV03798 | NC_031368.1  KX808498.1 | https://www.ncbi.nlm.nih.gov/nuccore/NC_031368.1  <https://www.ncbi.nlm.nih.gov/nuccore/KX808498.1> |
| *Derbesia* sp. WEST4838 | NC_031367.1  KX808497.1 | https://www.ncbi.nlm.nih.gov/nuccore/NC_031367.1  <https://www.ncbi.nlm.nih.gov/nuccore/KX808497.1> |
| *Scenedesmus* *obliquus* strain UTEX 393 | DQ396875.1 | <https://www.ncbi.nlm.nih.gov/nuccore/DQ396875.1> |
| *Pseudocharacium* *americanum* culture UTEX:2112 | NC_034711.1  KY407658.1 | https://www.ncbi.nlm.nih.gov/nuccore/NC_034711.1  <https://www.ncbi.nlm.nih.gov/nuccore/KY407658.1> |
| *Trichosarcina* *mucosa* culture SAG:4.90 | NC_034709.1  KY407656.1 | https://www.ncbi.nlm.nih.gov/nuccore/NC_034709.1  <https://www.ncbi.nlm.nih.gov/nuccore/KY407656.1> |
| *Ignatius* *tetrasporus* culture UTEX2012 | NC_034712.1  KY407659.1 | https://www.ncbi.nlm.nih.gov/nuccore/NC_034712.1  <https://www.ncbi.nlm.nih.gov/nuccore/KY407659.1> |
| *Neodangemannia* *microcystis* culture SAG:2022 | NC_034713.1  KY407660.1 | https://www.ncbi.nlm.nih.gov/nuccore/NC_034713.1  <https://www.ncbi.nlm.nih.gov/nuccore/KY407660.1> |
| *Pseudoneochloris* *marina* culture UTEX:1445 | NC_034710.1  KY407657.1 | https://www.ncbi.nlm.nih.gov/nuccore/NC_034710.1  <https://www.ncbi.nlm.nih.gov/nuccore/KY407657.1> |
| *Stigeoclonium* *helveticum* | NC_008372.1 | <https://www.ncbi.nlm.nih.gov/nuccore/NC_008372.1> |
| *Tetraselmis* sp. CCMP 881 | KU167097.1 | <https://www.ncbi.nlm.nih.gov/nuccore/KU167097.1> |
| *Coelastrella* *saipanensis* | NC_042181.1  MF423120.1 | https://www.ncbi.nlm.nih.gov/nuccore/NC_042181.1  <https://www.ncbi.nlm.nih.gov/nuccore/MF423120.1> |
| *Ulva* *fasciata* | NC_029040.1  KT882614.1 | https://www.ncbi.nlm.nih.gov/nuccore/NC_029040.1  <https://www.ncbi.nlm.nih.gov/nuccore/KT882614.1> |
| *Chlorella* *variabilis* isolate crs4cvariabilis2014 | KP271969.1 | <https://www.ncbi.nlm.nih.gov/nuccore/KP271969.1> |
| *Chlorella* *variabilis* isolate NC64A | KJ718922.1 | <https://www.ncbi.nlm.nih.gov/nuccore/KJ718922.1> |
| *Chlorella vulgaris* | NC_001865.1 | <https://www.ncbi.nlm.nih.gov/nuccore/NC_001865.1> |
| *Trebouxiophyceae* sp. MX-AZ01 | NC_018569.1  JX402620.1 | https://www.ncbi.nlm.nih.gov/nuccore/NC_018569.1  <https://www.ncbi.nlm.nih.gov/nuccore/JX402620.1> |
| *Chlorella* *sorokiniana* | NC_023835.1  KJ397925.1 | https://www.ncbi.nlm.nih.gov/nuccore/NC_023835.1  <https://www.ncbi.nlm.nih.gov/nuccore/KJ397925.1> |
| *Chlamydomonas* *reinhardtii* strain CC-373 ac-u-c-2-21 | MF083692.1 | <https://www.ncbi.nlm.nih.gov/nuccore/MF083692.1> |
| *Chlamydomonas* *reinhardtii* strain CC-2359 lts1-30 mt- | MF083689.2 | <https://www.ncbi.nlm.nih.gov/nuccore/MF083689.2> |
| *Chlamydomonas* *reinhardtii* strain CC-1375 ac-u-lambda mt+ | MF083690.1 | <https://www.ncbi.nlm.nih.gov/nuccore/MF083690.1> |
| *Chlamydomonas* *reinhardtii* strain CC-4199 lts1-204 | MF083688.1 | <https://www.ncbi.nlm.nih.gov/nuccore/MF083688.1> |
| *Chlamydomonas* *reinhardtii* strain CC-1051 M18 (ac9) mt+ | MF083691.1 | <https://www.ncbi.nlm.nih.gov/nuccore/MF083691.1> |
| *Palmophyllum* *crassum* chloroplast DNA | NC_033387.1  AP017927.1 | https://www.ncbi.nlm.nih.gov/nuccore/NC_033387.1  <https://www.ncbi.nlm.nih.gov/nuccore/AP017927.1> |
| *Cymbomonas* *tetramitiformis* strain PLY262 | NC_030169.1  KX013545.1 | https://www.ncbi.nlm.nih.gov/nuccore/NC_030169.1  <https://www.ncbi.nlm.nih.gov/nuccore/KX013545.1> |
| *Ulva* *prolifera* | NC_036137.1  KX342867.1 | https://www.ncbi.nlm.nih.gov/nuccore/NC_036137.1  <https://www.ncbi.nlm.nih.gov/nuccore/KX342867.1> |
| *Ulva* *flexuosa* | NC_035823.1  KX579943.1 | https://www.ncbi.nlm.nih.gov/nuccore/NC_035823.1  <https://www.ncbi.nlm.nih.gov/nuccore/KX579943.1> |
| *Caulerpa racemosa* | NC_032042.1  KT946602.1 | https://www.ncbi.nlm.nih.gov/nuccore/NC_032042.1  <https://www.ncbi.nlm.nih.gov/nuccore/KT946602.1> |
| *Botryococcus braunii* Showa | LT545991.1 | <https://www.ncbi.nlm.nih.gov/nuccore/LT545991.1> |
| *Pyramimonas parkeae* strain NIES254 | KX013546.1 | <https://www.ncbi.nlm.nih.gov/nuccore/KX013546.1> |
| *Neochloris aquatica* strain UTEX 138 | NC_029670.1  KT199248.1 | https://www.ncbi.nlm.nih.gov/nuccore/NC_029670.1  <https://www.ncbi.nlm.nih.gov/nuccore/KT199248.1> |
| *Bracteacoccus aerius* strain UTEX 1250 | NC_029675.1  KT199254.1 | https://www.ncbi.nlm.nih.gov/nuccore/NC_029675.1  <https://www.ncbi.nlm.nih.gov/nuccore/KT199254.1> |
| *Bracteacoccus minor* strain UTEX 66 | NC_029674.1  KT199253.1 | https://www.ncbi.nlm.nih.gov/nuccore/NC_029674.1  <https://www.ncbi.nlm.nih.gov/nuccore/KT199253.1> |
| *Ankyra judayi* strain SAG 17.84 | NC_029735.1  KT199255.1 | https://www.ncbi.nlm.nih.gov/nuccore/NC_029735.1  <https://www.ncbi.nlm.nih.gov/nuccore/KT199255.1> |
| *Chromochloris zofingiensis* strain UTEX 56 | NC_029672.1  KT199251.1 | https://www.ncbi.nlm.nih.gov/nuccore/NC_029672.1  <https://www.ncbi.nlm.nih.gov/nuccore/KT199251.1> |
| *Pseudomuriella schumacherensis* strain SAG 2137 | NC_029669.1  KT199256.1 | https://www.ncbi.nlm.nih.gov/nuccore/NC_029669.1  <https://www.ncbi.nlm.nih.gov/nuccore/KT199256.1> |
| *Mychonastes homosphaera* strain CAUP H6502 | NC_029671.1  KT199249.1 | https://www.ncbi.nlm.nih.gov/nuccore/NC_029671.1  <https://www.ncbi.nlm.nih.gov/nuccore/KT199249.1> |
| *Gloeotilopsis sterilis* culture-collection UTEX:1704 | NC_025538.1  KM462877.1 | https://www.ncbi.nlm.nih.gov/nuccore/NC_025538.1  <https://www.ncbi.nlm.nih.gov/nuccore/KM462877.1> |
| *Microthamnion kuetzingianum* culture-collection UTEX:318 | NC_025537.1  KM462876.1 | https://www.ncbi.nlm.nih.gov/nuccore/NC_025537.1  <https://www.ncbi.nlm.nih.gov/nuccore/KM462876.1> |
| *Fusochloris perforata* culture-collection SAG:28.85 | NC_025543.1  KM462882.1 | https://www.ncbi.nlm.nih.gov/nuccore/NC_025543.1  <https://www.ncbi.nlm.nih.gov/nuccore/KM462882.1> |
| *Paradoxia* *multiseta* culture-collection SAG:18.84 | NC_025540.1  KM462879.1 | https://www.ncbi.nlm.nih.gov/nuccore/NC_025540.1  <https://www.ncbi.nlm.nih.gov/nuccore/KM462879.1> |
| *Xylochloris* *irregularis* culture-collection CAUP<CZH>:H7801 | NC_025534.1  KM462872.1 | https://www.ncbi.nlm.nih.gov/nuccore/NC_025534.1  <https://www.ncbi.nlm.nih.gov/nuccore/KM462872.1> |
| *Chlorella* *mirabilis* culture-collection SAG:38.88 | NC_025528.1  KM462865.1 | https://www.ncbi.nlm.nih.gov/nuccore/NC_025528.1  <https://www.ncbi.nlm.nih.gov/nuccore/KM462865.1> |
| *Botryococcus braunii* culture-collection SAG:807-1 | NC_025545.1  KM462884.1 | https://www.ncbi.nlm.nih.gov/nuccore/NC_025545.1  <https://www.ncbi.nlm.nih.gov/nuccore/KM462884.1> |
| *Pedinomonas tuberculata* culture-collection SAG:42.84 | NC_025530.1  KM462867.1 | https://www.ncbi.nlm.nih.gov/nuccore/NC_025530.1  <https://www.ncbi.nlm.nih.gov/nuccore/KM462867.1> |
| *Neocystis brevis* culture-collection CAUP<CZH>:D802 | NC_025535.1  KM462873.1 | https://www.ncbi.nlm.nih.gov/nuccore/NC_025535.1  <https://www.ncbi.nlm.nih.gov/nuccore/KM462873.1> |
| *Lobosphaera incisa* culture-collection SAG:2007 | NC_025533.1  KM462871.1 | https://www.ncbi.nlm.nih.gov/nuccore/NC_025533.1  <https://www.ncbi.nlm.nih.gov/nuccore/KM462871.1> |
| *Watanabea reniformis* culture-collection SAG:211-9b | NC_025526.1  KM462863.1 | https://www.ncbi.nlm.nih.gov/nuccore/NC_025526.1  <https://www.ncbi.nlm.nih.gov/nuccore/KM462863.1> |
| *Pabia signiensis* culture-collection SAG:7.90 | NC_025529.1  KM462866.1 | https://www.ncbi.nlm.nih.gov/nuccore/NC_025529.1  <https://www.ncbi.nlm.nih.gov/nuccore/KM462866.1> |
| *Myrmecia israelensis* culture-collection UTEX:1181 | NC_025525.1  KM462861.1 | https://www.ncbi.nlm.nih.gov/nuccore/NC_025525.1  <https://www.ncbi.nlm.nih.gov/nuccore/KM462861.1> |
| *Prasiolopsis* sp. SAG 84.81 | KM462862.1 | <https://www.ncbi.nlm.nih.gov/nuccore/KM462862.1> |
| *Pseudochloris* *wilhelmii* culture-collection SAG:1.80 | NC_025547.1  KM462886.1 | https://www.ncbi.nlm.nih.gov/nuccore/NC_025547.1  <https://www.ncbi.nlm.nih.gov/nuccore/KM462886.1> |
| *Geminella minor* culture-collection SAG:22.88 | NC_025544.1  KM462883.1 | https://www.ncbi.nlm.nih.gov/nuccore/NC_025544.1  <https://www.ncbi.nlm.nih.gov/nuccore/KM462883.1> |
| *Dicloster* *acuatus* culture-collection SAG:41.98 | NC_025546.1  KM462885.1 | https://www.ncbi.nlm.nih.gov/nuccore/NC_025546.1  <https://www.ncbi.nlm.nih.gov/nuccore/KM462885.1> |
| *Marvania geminata* culture-collection SAG:12.88 | NC_025549.1  KM462888.1 | https://www.ncbi.nlm.nih.gov/nuccore/NC_025549.1  <https://www.ncbi.nlm.nih.gov/nuccore/KM462888.1> |
| *Planctonema lauterbornii* culture-collection SAG:68.94 | NC_025541.1  KM462880.1 | https://www.ncbi.nlm.nih.gov/nuccore/NC_025541.1  <https://www.ncbi.nlm.nih.gov/nuccore/KM462880.1> |
| *Koliella longiseta* culture-collection UTEX:339 | NC_025531.1 | <https://www.ncbi.nlm.nih.gov/nuccore/NC_025531.1> |
| *Stichococcus bacillaris* culture-collection UTEX:176 | NC_025527.1  KM462864.1 | https://www.ncbi.nlm.nih.gov/nuccore/NC_025527.1  <https://www.ncbi.nlm.nih.gov/nuccore/KM462864.1> |
| *Elliptochloris bilobata* culture-collection CAUP<CZH>:H7103 | NC_025548.1  KM462887.1 | https://www.ncbi.nlm.nih.gov/nuccore/NC_025548.1  <https://www.ncbi.nlm.nih.gov/nuccore/KM462887.1> |
| *Marsupiomonas* sp. NIES 1824 | KM462870.1 | <https://www.ncbi.nlm.nih.gov/nuccore/KM462870.1> |
| *Jenufa* *minuta* culture-collection CAUP:H 8102 | NC_028582.1  KT625414.1 | https://www.ncbi.nlm.nih.gov/nuccore/NC_028582.1  <https://www.ncbi.nlm.nih.gov/nuccore/KT625414.1> |
| *Jenufa perforata* culture-collection CAUP:H 8101 | NC_028581.1  KT625413.1 | https://www.ncbi.nlm.nih.gov/nuccore/NC_028581.1  <https://www.ncbi.nlm.nih.gov/nuccore/KT625413.1> |
| *Treubaria triappendiculata* culture-collection SAG:38.83 | NC_028578.1 | <https://www.ncbi.nlm.nih.gov/nuccore/NC_028578.1> |
| *Carteria cerasiformis* culture-collection NIES:425 | NC_028585.1  KT625420.1 | https://www.ncbi.nlm.nih.gov/nuccore/NC_028585.1  <https://www.ncbi.nlm.nih.gov/nuccore/KT625420.1> |
| *Carteria* sp. SAG 8-5 | KT625419.1 | <https://www.ncbi.nlm.nih.gov/nuccore/KT625419.1> |
| *Oogamochlamys gigantea* culture-collection SAG:44.91 | NC_028580.1  KT625412.1 | https://www.ncbi.nlm.nih.gov/nuccore/NC_028580.1  <https://www.ncbi.nlm.nih.gov/nuccore/KT625412.1> |
| *Characiochloris acuminata* culture-collection SAG:31.95 | NC_028584.1  KT625418.1 | https://www.ncbi.nlm.nih.gov/nuccore/NC_028584.1  <https://www.ncbi.nlm.nih.gov/nuccore/KT625418.1> |
| *Hafniomonas laevis* culture-collection NIES:257 | NC_028583.1  KT625415.1 | https://www.ncbi.nlm.nih.gov/nuccore/NC_028583.1  <https://www.ncbi.nlm.nih.gov/nuccore/KT625415.1> |
| *Phacotus lenticularis* culture-collection SAG:61-1 | NC_028587.1  KT625422.1 | https://www.ncbi.nlm.nih.gov/nuccore/NC_028587.1  <https://www.ncbi.nlm.nih.gov/nuccore/KT625422.1> |
| *Bracteacoccus giganteus* culture-collection UTEX:1251 | NC_028586.1  KT625421.1 | https://www.ncbi.nlm.nih.gov/nuccore/NC_028586.1  <https://www.ncbi.nlm.nih.gov/nuccore/KT625421.1> |
| *Mychonastes jurisii* culture-collection SAG:37.98 | NC_028579.1 | <https://www.ncbi.nlm.nih.gov/nuccore/NC_028579.1> |
| *Oedogonium cardiacum* strain SAG 575-1b | EU677193.1 | <https://www.ncbi.nlm.nih.gov/nuccore/EU677193.1> |
| *Oedogonium cardiacum* | NC_011031.1 | <https://www.ncbi.nlm.nih.gov/nuccore/NC_011031.1> |
| *Caulerpa lentillifera* | NC_039377.1  MG753774.1 | https://www.ncbi.nlm.nih.gov/nuccore/NC_039377.1  <https://www.ncbi.nlm.nih.gov/nuccore/MG753774.1> |
| *Trentepohlia odorata* | MK580484.1 | <https://www.ncbi.nlm.nih.gov/nuccore/MK580484.1> |
| *Chlamydomonas* sp. UWO241 | MH590838.1 | <https://www.ncbi.nlm.nih.gov/nuccore/MH590838.1> |
| *Prototheca* *wickerhamii* strain SAG 263-11 | KJ001761.1 | <https://www.ncbi.nlm.nih.gov/nuccore/KJ001761.1> |
| *Auxenochlorella protothecoides* | KC843975.1 | <https://www.ncbi.nlm.nih.gov/nuccore/KC843975.1> |
| *Hariotina* sp. MMOGRB0030F | KX131180.1 | <https://www.ncbi.nlm.nih.gov/nuccore/KX131180.1> |
| *Tetradesmus obliquus* | KX756229.1 | <https://www.ncbi.nlm.nih.gov/nuccore/KX756229.1> |
| *Pectinodesmus pectinatus* | NC_036668.1  KU847995.1 | https://www.ncbi.nlm.nih.gov/nuccore/NC_036668.1  <https://www.ncbi.nlm.nih.gov/nuccore/KU847995.1> |
| *Stauridium* *tetras* voucher ACOI 84 | NC_037923.1  MF276986.1 | https://www.ncbi.nlm.nih.gov/nuccore/NC_037923.1  <https://www.ncbi.nlm.nih.gov/nuccore/MF276986.1> |
| *Pseudopediastrum* sp. CL0201VA | NC_037922.1  MF276985.1 | https://www.ncbi.nlm.nih.gov/nuccore/NC_037922.1  <https://www.ncbi.nlm.nih.gov/nuccore/MF276985.1> |
| *Pseudopediastrum integrum* voucher ACOI 577 | NC_037921.1  MF276984.1 | https://www.ncbi.nlm.nih.gov/nuccore/NC_037921.1  <https://www.ncbi.nlm.nih.gov/nuccore/MF276984.1> |
| *Pseudopediastrum* *boryanum* voucher IL0402MN | NC_037920.1  MF276982.1 | https://www.ncbi.nlm.nih.gov/nuccore/NC_037920.1  <https://www.ncbi.nlm.nih.gov/nuccore/MF276982.1> |
| *Pediastrum* *angulosum* voucher *Pediastrum* *angulosum* strain ACOI 1354 | NC_037919.1  MF276977.1 | https://www.ncbi.nlm.nih.gov/nuccore/NC_037919.1  <https://www.ncbi.nlm.nih.gov/nuccore/MF276977.1> |
| *Pediastrum angulosum* voucher *Pediastrum* *angulosum* KP0301SC | MF276978.1 | <https://www.ncbi.nlm.nih.gov/nuccore/MF276978.1> |
| *Pediastrum* *duplex* voucher PL0501b | MF276980.1 | <https://www.ncbi.nlm.nih.gov/nuccore/MF276980.1> |
| *Pediastrum* *duplex* voucher SL0404MN | MF276981.1 | <https://www.ncbi.nlm.nih.gov/nuccore/MF276981.1> |
| *Pediastrum* *duplex* voucher EL0201CT | NC_034654.1  KY114064.1 | https://www.ncbi.nlm.nih.gov/nuccore/NC_034654.1  <https://www.ncbi.nlm.nih.gov/nuccore/KY114064.1> |
| *Lacunastrum* *gracillimum* voucher ACOI 392 | NC_037918.1  MF276976.1 | https://www.ncbi.nlm.nih.gov/nuccore/NC_037918.1  <https://www.ncbi.nlm.nih.gov/nuccore/MF276976.1> |
| *Stigeoclonium* *helveticum* strain UTEX 441 | DQ630521.1 | <https://www.ncbi.nlm.nih.gov/nuccore/DQ630521.1> |
| *Floydiella* *terrestris* culture-collection UTEX:1709 | GU196268.1 | <https://www.ncbi.nlm.nih.gov/nuccore/GU196268.1> |
| *Oedocladium* *carolinianum* culture-collection UTEX:LB 1686 | NC_031510.1  KX507373.1 | https://www.ncbi.nlm.nih.gov/nuccore/NC_031510.1  <https://www.ncbi.nlm.nih.gov/nuccore/KX507373.1> |
| *Chlamydomonas reinhardtii* | BK000554.2 | <https://www.ncbi.nlm.nih.gov/nuccore/BK000554.2> |
| *Gonium* *pectorale* chloroplast DNA,isolate: K3-F3-4 | AP012494.1 | <https://www.ncbi.nlm.nih.gov/nuccore/AP012494.1> |
| *Ettlia* *pseudoalveolaris* culture-collection UTEX:975 | NC_025532.1  KM462869.1 | https://www.ncbi.nlm.nih.gov/nuccore/NC_025532.1  <https://www.ncbi.nlm.nih.gov/nuccore/KM462869.1> |
| *Spermatozopsis* *similis* culture SAG:B 1.85 | NC_042251.1  MG778500.1 | https://www.ncbi.nlm.nih.gov/nuccore/NC_042251.1  <https://www.ncbi.nlm.nih.gov/nuccore/MG778500.1> |
| *Chlorococcum* *tatrense* culture UTEX:2227 | NC_042182.1  MG778173.1 | https://www.ncbi.nlm.nih.gov/nuccore/NC_042182.1  <https://www.ncbi.nlm.nih.gov/nuccore/MG778173.1> |
| *Chlorosarcinopsis* *eremi* culture UTEX:1186 | NC_042250.1  MG778185.1 | https://www.ncbi.nlm.nih.gov/nuccore/NC_042250.1  <https://www.ncbi.nlm.nih.gov/nuccore/MG778185.1> |
| *Haematococcus lacustris* voucher UTEX:2505 | NC_037007.1  MG677935.1 | https://www.ncbi.nlm.nih.gov/nuccore/NC_037007.1  <https://www.ncbi.nlm.nih.gov/nuccore/MG677935.1> |
| *Pleodorina starrii* | JX977846.1  NC_021109.1 | https://www.ncbi.nlm.nih.gov/nuccore/JX977846.1  <https://www.ncbi.nlm.nih.gov/nuccore/NC_021109.1> |
| *Parallela transversalis* strain UTEX LB 1252 | NC_042241.1  MG786420.1 | https://www.ncbi.nlm.nih.gov/nuccore/NC_042241.1  <https://www.ncbi.nlm.nih.gov/nuccore/MG786420.1> |
| *Kirchneriella aperta* strain SAG 2004 | NC_029676.1  KT199250.1 | https://www.ncbi.nlm.nih.gov/nuccore/NC_029676.1  <https://www.ncbi.nlm.nih.gov/nuccore/KT199250.1> |
| *Scherffelia dubia* culture-collection SAG:17.86 | NC_029807.1  KU167098.1 | https://www.ncbi.nlm.nih.gov/nuccore/NC_029807.1  <https://www.ncbi.nlm.nih.gov/nuccore/KU167098.1> |
| *Interfilum terricola* culture-collection SAG:20.91 | NC_025542.1  KM462881.1 | https://www.ncbi.nlm.nih.gov/nuccore/NC_025542.1  <https://www.ncbi.nlm.nih.gov/nuccore/KM462881.1> |
| *Ulva linza* isolate QD08 | NC_030312.1  KX058323.1 | https://www.ncbi.nlm.nih.gov/nuccore/NC_030312.1  <https://www.ncbi.nlm.nih.gov/nuccore/KX058323.1> |
| *Ulva mutabilis* | MK069584.1 | <https://www.ncbi.nlm.nih.gov/nuccore/MK069584.1> |
| *Capsosiphon fulvescens* isolate typeB | MH790906.1 | <https://www.ncbi.nlm.nih.gov/nuccore/MH790906.1> |
| *Hazenia capsulata* culture UTEX:1918 | KY407661.1 | <https://www.ncbi.nlm.nih.gov/nuccore/KY407661.1> |
| *Hazenia capsulata* | NC_034714.1 | <https://www.ncbi.nlm.nih.gov/nuccore/NC_034714.1> |
| *Caulerpa okamurae* | KX809677.1 | <https://www.ncbi.nlm.nih.gov/nuccore/KX809677.1> |
| *Codium* sp. 'arenicola' | NC_037366.1  KY819066.1 | https://www.ncbi.nlm.nih.gov/nuccore/NC_037366.1  <https://www.ncbi.nlm.nih.gov/nuccore/KY819066.1> |
| *Ostreobium* sp. OS1B | KU979013.1 | <https://www.ncbi.nlm.nih.gov/nuccore/KU979013.1> |
| *Micractinium* sp. LBA 32 | MH983006.1 | <https://www.ncbi.nlm.nih.gov/nuccore/MH983006.1> |
| *Chlorella* sp. ATCC 30562 | KY629617.1 | <https://www.ncbi.nlm.nih.gov/nuccore/KY629617.1> |
| *Coccomyxa* sp. SUA001 | MF805805.1 | <https://www.ncbi.nlm.nih.gov/nuccore/MF805805.1> |
| *Coccomyxa* sp. C-169 | NC_015084.1  HQ693844.1 | https://www.ncbi.nlm.nih.gov/nuccore/NC_015084.1  <https://www.ncbi.nlm.nih.gov/nuccore/HQ693844.1> |
| *Choricystis parasitica* culture-collection SAG:17.98 | NC_025539.1  KM462878.1 | https://www.ncbi.nlm.nih.gov/nuccore/NC_025539.1  <https://www.ncbi.nlm.nih.gov/nuccore/KM462878.1> |
| *Leptosira terrestris* | NC_009681.1 | <https://www.ncbi.nlm.nih.gov/nuccore/NC_009681.1> |
| *Symbiochloris handae* culture-collection SAG:2150 | NC_025524.1  KM462860.1 | https://www.ncbi.nlm.nih.gov/nuccore/NC_025524.1  <https://www.ncbi.nlm.nih.gov/nuccore/KM462860.1> |
| *Chlorella sorokiniana* isolate 1230 | KJ742376.1 | <https://www.ncbi.nlm.nih.gov/nuccore/KJ742376.1> |
| *Chlorella variabilis* | NC_015359.1  HQ914635.1 | https://www.ncbi.nlm.nih.gov/nuccore/NC_015359.1  <https://www.ncbi.nlm.nih.gov/nuccore/HQ914635.1> |
| *Auxenochlorella protothecoides* strain UTEX 2341 | KY613608.1 | <https://www.ncbi.nlm.nih.gov/nuccore/KY613608.1> |
| *Auxenochlorella protothecoides* | NC_023775.1  KC631634.1 | https://www.ncbi.nlm.nih.gov/nuccore/NC_023775.1  <https://www.ncbi.nlm.nih.gov/nuccore/KC631634.1> |
| *Koliella corcontica* culture-collection SAG:24.84 | NC_025536.1  KM462874.1 | https://www.ncbi.nlm.nih.gov/nuccore/NC_025536.1  <https://www.ncbi.nlm.nih.gov/nuccore/KM462874.1> |
| *Micromonas* sp. RCC299 | NC_012575.1  FJ858267.1 | https://www.ncbi.nlm.nih.gov/nuccore/NC_012575.1  <https://www.ncbi.nlm.nih.gov/nuccore/FJ858267.1> |
| *Bathycoccus prasinos* | NC_024811.1 | <https://www.ncbi.nlm.nih.gov/nuccore/NC_024811.1> |
| *Monomastix* sp. OKE-1 | NC_012101.1  FJ493497.1 | https://www.ncbi.nlm.nih.gov/nuccore/NC_012101.1  <https://www.ncbi.nlm.nih.gov/nuccore/FJ493497.1> |
| *Pedinophyceae* sp. YPF-701 | KY347917.1 | <https://www.ncbi.nlm.nih.gov/nuccore/KY347917.1> |
| *Guillardia theta* | NC_000926.1  AF041468.1 | https://www.ncbi.nlm.nih.gov/nuccore/NC_000926.1  <https://www.ncbi.nlm.nih.gov/nuccore/AF041468.1> |
| *Teleaulax amphioxeia* strain HACCP-CR01 | NC_027589.1  KP899713.1 | https://www.ncbi.nlm.nih.gov/nuccore/NC_027589.1  <https://www.ncbi.nlm.nih.gov/nuccore/KP899713.1> |
| *Cryptomonas paramecium* | NC_013703.1 | <https://www.ncbi.nlm.nih.gov/nuccore/NC_013703.1> |
| *Guillardia theta* voucher CCMP:2712 | KT428890.1 | <https://www.ncbi.nlm.nih.gov/nuccore/KT428890.1> |
| *Rhodomonas salina* strain CCMP1319 | EF508371.1 | <https://www.ncbi.nlm.nih.gov/nuccore/EF508371.1> |
| *Chroomonas mesostigmatica* CCMP1168 | KY860574.1 | <https://www.ncbi.nlm.nih.gov/nuccore/KY860574.1> |
| *Rhodomonas salina* | NC_009573.1 | <https://www.ncbi.nlm.nih.gov/nuccore/NC_009573.1> |
| *Cryptomonas paramecium* strain CCAP9772a | GQ358203.1 | <https://www.ncbi.nlm.nih.gov/nuccore/GQ358203.1> |
| *Eutreptiella gymnastica* | NC_017754.2 | <https://www.ncbi.nlm.nih.gov/nuccore/NC_017754.2> |
| *Eutreptiella gymnastica* strain K-0333 | HE605038.1 | <https://www.ncbi.nlm.nih.gov/nuccore/HE605038.1> |
| *Euglena mutabilis* voucher SAG 1224-9b | KT223519.1 | <https://www.ncbi.nlm.nih.gov/nuccore/KT223519.1> |
| *Euglena hiemalis* strain CCAP1224.35 | NC_039156.1  MF622086.1 | https://www.ncbi.nlm.nih.gov/nuccore/NC_039156.1  <https://www.ncbi.nlm.nih.gov/nuccore/MF622086.1> |
| *Monomorphina aenigmatica* strain UTEX1284 | NC_020018.1  JX457480.1 | https://www.ncbi.nlm.nih.gov/nuccore/NC_020018.1  <https://www.ncbi.nlm.nih.gov/nuccore/JX457480.1> |
| *Lepocinclis steinii* voucher UTEX 523 | NC_039971.1  MH898672.1 | https://www.ncbi.nlm.nih.gov/nuccore/NC_039971.1  <https://www.ncbi.nlm.nih.gov/nuccore/MH898672.1> |
| *Lepocinclis playfairiana* voucher MI 102 | NC_039970.1  MH898671.1 | https://www.ncbi.nlm.nih.gov/nuccore/NC_039970.1  <https://www.ncbi.nlm.nih.gov/nuccore/MH898671.1> |
| *Lepocinclis tripteris* voucher MI 101 | NC_039968.1  MH898668.1 | https://www.ncbi.nlm.nih.gov/nuccore/NC_039968.1  <https://www.ncbi.nlm.nih.gov/nuccore/MH898668.1> |
| *Lepocinclis tripteris* voucher UTEX 1331 | MH898669.1 | <https://www.ncbi.nlm.nih.gov/nuccore/MH898669.1> |
| *Lepocinclis ovum* voucher SAG 1244-8 | NC_039928.1  MH898674.1 | https://www.ncbi.nlm.nih.gov/nuccore/NC_039928.1  <https://www.ncbi.nlm.nih.gov/nuccore/MH898674.1> |
| *Phacus inflexus* voucher ACOI 1336 | NC_039967.1  MH898667.1 | https://www.ncbi.nlm.nih.gov/nuccore/NC_039967.1  <https://www.ncbi.nlm.nih.gov/nuccore/MH898667.1> |
| *Phacus pleuronectes* voucher SAG 1261-3b | NC_039927.1  MH898673.1 | https://www.ncbi.nlm.nih.gov/nuccore/NC_039927.1  <https://www.ncbi.nlm.nih.gov/nuccore/MH898673.1> |
| *Discoplastis spathirhyncha* voucher SAG 1224-42 | NC_039969.1  MH898670.1 | https://www.ncbi.nlm.nih.gov/nuccore/NC_039969.1  <https://www.ncbi.nlm.nih.gov/nuccore/MH898670.1> |
| *Euglena longa* | NC_002652.1 | <https://www.ncbi.nlm.nih.gov/nuccore/NC_002652.1> |
| *Astasia longa* | AJ294725.1 | <https://www.ncbi.nlm.nih.gov/nuccore/AJ294725.1> |
| *Euglena gracilis* var. *bacillaris* strain SAG 1224-5/15 | KP686076.1 | <https://www.ncbi.nlm.nih.gov/nuccore/KP686076.1> |
| *Euglena viridis* strain SAG 1224-17d | KP686075.1 | <https://www.ncbi.nlm.nih.gov/nuccore/KP686075.1> |
| *Euglenaria anabaena* strain UTEX 373 | NC_027269.1  KP453743.1 | https://www.ncbi.nlm.nih.gov/nuccore/NC_027269.1  <https://www.ncbi.nlm.nih.gov/nuccore/KP453743.1> |
| *Cryptoglena skujai* strain SAG 10.88 | NC_027286.1  KP410781.1 | https://www.ncbi.nlm.nih.gov/nuccore/NC_027286.1  <https://www.ncbi.nlm.nih.gov/nuccore/KP410781.1> |
| *Trachelomonas volvocina* strain UTEX 1327 | NC_027288.1  KP686077.1 | https://www.ncbi.nlm.nih.gov/nuccore/NC_027288.1  <https://www.ncbi.nlm.nih.gov/nuccore/KP686077.1> |
| *Monomorphina parapyrum* strain UTEX 2354 culture-collection UTEX:2354 | NC_027287.1  KP455987.1 | https://www.ncbi.nlm.nih.gov/nuccore/NC_027287.1  <https://www.ncbi.nlm.nih.gov/nuccore/KP455987.1> |
| *Euglenaformis proxima* strain SAG 1224-11a | NC_024154.1  KC684276.1 | https://www.ncbi.nlm.nih.gov/nuccore/NC_024154.1  <https://www.ncbi.nlm.nih.gov/nuccore/KC684276.1> |
| *Euglena gracilis* | NC_001603.2  X70810.2 | https://www.ncbi.nlm.nih.gov/nuccore/NC_001603.2  <https://www.ncbi.nlm.nih.gov/nuccore/X70810.2> |
| *Euglena archaeoplastidiata* isolate MI 67 | NC_035156.1  KP939040.1 | https://www.ncbi.nlm.nih.gov/nuccore/NC_035156.1  <https://www.ncbi.nlm.nih.gov/nuccore/KP939040.1> |
| *Euglena clara* strain SAG 25.98 | NC_038187.1  MF630936.1 | https://www.ncbi.nlm.nih.gov/nuccore/NC_038187.1  <https://www.ncbi.nlm.nih.gov/nuccore/MF630936.1> |
| *Euglena viridis* culture-collection ATCC:PRA110 | NC_020460.2  JQ237893.2 | https://www.ncbi.nlm.nih.gov/nuccore/NC_020460.2  <https://www.ncbi.nlm.nih.gov/nuccore/JQ237893.2> |
| *Gloeochaete wittrockiana* strain SAG 46.84 | NC_040153.1  MF167426.1 | https://www.ncbi.nlm.nih.gov/nuccore/NC_040153.1  <https://www.ncbi.nlm.nih.gov/nuccore/MF167426.1> |
| *Glaucocystis incrassata* strain SAG 229-2 | NC_040152.1  MF167425.1 | https://www.ncbi.nlm.nih.gov/nuccore/NC_040152.1  <https://www.ncbi.nlm.nih.gov/nuccore/MF167425.1> |
| *Glaucocystis* sp. BBH | MF167424.1 | <https://www.ncbi.nlm.nih.gov/nuccore/MF167424.1> |
| *Cyanophora* *biloba* strain UTEX LB 2766 | NC_038216.1  MG601103.1 | https://www.ncbi.nlm.nih.gov/nuccore/NC_038216.1  <https://www.ncbi.nlm.nih.gov/nuccore/MG601103.1> |
| *Cyanophora* *sudae* strain NIES-764 | NC_038215.1  MG601102.1 | https://www.ncbi.nlm.nih.gov/nuccore/NC_038215.1  <https://www.ncbi.nlm.nih.gov/nuccore/MG601102.1> |
| *Emiliania* *huxleyi* culture-collection CCMP:1516 | JN022705.1 | <https://www.ncbi.nlm.nih.gov/nuccore/JN022705.1> |
| *Chrysochromulina* sp. CCMP291 | KJ201907.2 | <https://www.ncbi.nlm.nih.gov/nuccore/KJ201907.2> |
| *Emiliania* *huxleyi* | NC_007288.1 | <https://www.ncbi.nlm.nih.gov/nuccore/NC_007288.1> |
| *Emiliania* *huxleyi* strain CCMP 373 | AY741371.1 | <https://www.ncbi.nlm.nih.gov/nuccore/AY741371.1> |
| *Tisochrysis* *lutea* | NC_040291.1  MF795089.2 | https://www.ncbi.nlm.nih.gov/nuccore/NC_040291.1  <https://www.ncbi.nlm.nih.gov/nuccore/MF795089.2> |
| *Pavlova lutheri* | NC_020371.1  KC573041.1 | https://www.ncbi.nlm.nih.gov/nuccore/NC_020371.1  <https://www.ncbi.nlm.nih.gov/nuccore/KC573041.1> |
| *Phaeocystis globosa* strain Pg-G(A) | NC_021637.1  KC900889.1 | https://www.ncbi.nlm.nih.gov/nuccore/NC_021637.1  <https://www.ncbi.nlm.nih.gov/nuccore/KC900889.1> |
| *Phaeocystis* *antarctica* strain CCMP1374 | NC_016703.2  JN117275.2 | https://www.ncbi.nlm.nih.gov/nuccore/NC_016703.2  <https://www.ncbi.nlm.nih.gov/nuccore/JN117275.2> |
| *Chrysochromulina parva* | NC_036937.1  MG520331.1 | https://www.ncbi.nlm.nih.gov/nuccore/NC_036937.1  <https://www.ncbi.nlm.nih.gov/nuccore/MG520331.1> |
| *Durinskia baltica* | NC_014287.1 | <https://www.ncbi.nlm.nih.gov/nuccore/NC_014287.1> |
| *Durinskia baltica* strain CS-38 | GU591327.1 | <https://www.ncbi.nlm.nih.gov/nuccore/GU591327.1> |
| *Kryptoperidinium foliaceum* | NC_014267.1 | <https://www.ncbi.nlm.nih.gov/nuccore/NC_014267.1> |
| *Kryptoperidinium foliaceum* strain CCMP1326 | GU591328.1 | <https://www.ncbi.nlm.nih.gov/nuccore/GU591328.1> |
| *Lepidodinium* *chlorophorum* | NC_027093.1  LC008447.1 | https://www.ncbi.nlm.nih.gov/nuccore/NC_027093.1  <https://www.ncbi.nlm.nih.gov/nuccore/LC008447.1> |
| *Ochromonas* sp. CCMP1393 | KJ877675.1 | <https://www.ncbi.nlm.nih.gov/nuccore/KJ877675.1> |
| *Trachydiscus minutus* | NC_026851.1  KJ624065 | https://www.ncbi.nlm.nih.gov/nuccore/NC_026851.1  <https://www.ncbi.nlm.nih.gov/nuccore/KJ624065> |
| *Monodopsis* sp. MarTras21 | KX839260.1 | <https://www.ncbi.nlm.nih.gov/nuccore/KX839260.1> |
| *Vischeria* sp. CAUP Q 202 | KX839261.1 | <https://www.ncbi.nlm.nih.gov/nuccore/KX839261.1> |
| *Fucus* *vesiculosus* | NC_016735.1  FM957154.1 | https://www.ncbi.nlm.nih.gov/nuccore/NC_016735.1  <https://www.ncbi.nlm.nih.gov/nuccore/FM957154.1> |
| *Ectocarpus* *siliculosus* | NC_013498.1  FP102343.1 | https://www.ncbi.nlm.nih.gov/nuccore/NC_013498.1  <https://www.ncbi.nlm.nih.gov/nuccore/FP102343.1> |
| *Eustigmatophyceae* sp. Ndem 8/9T-3m6.8 | NC_040298.1  MK281456.1 | https://www.ncbi.nlm.nih.gov/nuccore/NC_040298.1  <https://www.ncbi.nlm.nih.gov/nuccore/MK281456.1> |
| *Eustigmatophyceae* sp. Mont 10/10-1w | NC_040297.1  MK281455.1 | https://www.ncbi.nlm.nih.gov/nuccore/NC_040297.1  <https://www.ncbi.nlm.nih.gov/nuccore/MK281455.1> |
| *Eustigmatophyceae* sp. Chic 10/23 P-6w | NC_040296.1  MK281454.1 | https://www.ncbi.nlm.nih.gov/nuccore/NC_040296.1  <https://www.ncbi.nlm.nih.gov/nuccore/MK281454.1> |
| *Eustigmatophyceae* sp. Bat 8/9-7w | NC_040295.1  MK281453.1 | https://www.ncbi.nlm.nih.gov/nuccore/NC_040295.1  <https://www.ncbi.nlm.nih.gov/nuccore/MK281453.1> |
| *Vischeria* sp. ACOI 3415 | NC_040300.1  MK281458.1 | https://www.ncbi.nlm.nih.gov/nuccore/NC_040300.1  <https://www.ncbi.nlm.nih.gov/nuccore/MK281458.1> |
| *Pseudellipsoidion* *edaphicum* CAUP Q 401 | NC_040299.1  MK281457.1 | https://www.ncbi.nlm.nih.gov/nuccore/NC_040299.1  <https://www.ncbi.nlm.nih.gov/nuccore/MK281457.1> |
| *Characiopsis* *acuta* strain ACOI 456 | NC_040294.1  MK281452.1 | https://www.ncbi.nlm.nih.gov/nuccore/NC_040294.1  <https://www.ncbi.nlm.nih.gov/nuccore/MK281452.1> |
| *Dictyopteris divaricata* | NC_036804.1  KY433579.1 | https://www.ncbi.nlm.nih.gov/nuccore/NC_036804.1  <https://www.ncbi.nlm.nih.gov/nuccore/KY433579.1> |
| *Sargassum* *confusum* | MG459429.1 | <https://www.ncbi.nlm.nih.gov/nuccore/MG459429.1> |
| *Nannochloropsis* *oceanica* CCMP531 | NC_022263.1  KC598090.1 | https://www.ncbi.nlm.nih.gov/nuccore/NC_022263.1  <https://www.ncbi.nlm.nih.gov/nuccore/KC598090.1> |
| Nannochloropsis oceanica IMET1 | KC598086.1 | <https://www.ncbi.nlm.nih.gov/nuccore/KC598086.1> |
| *Nannochloropsis* *gaditana* CCMP527 | KC598084.1 | <https://www.ncbi.nlm.nih.gov/nuccore/KC598084.1> |
| *Nannochloropsis* *salina* CCMP537 | NC_022261.1  KC598088.1 | https://www.ncbi.nlm.nih.gov/nuccore/NC_022261.1  <https://www.ncbi.nlm.nih.gov/nuccore/KC598088.1> |
| *Nannochloropsis* *limnetica* CCMP505 | NC_022262.1  KC598089.1 | https://www.ncbi.nlm.nih.gov/nuccore/NC_022262.1  <https://www.ncbi.nlm.nih.gov/nuccore/KC598089.1> |
| *Nannochloropsis* *oculata* CCMP525 | NC_022260.1  KC598087.1 | https://www.ncbi.nlm.nih.gov/nuccore/NC_022260.1  <https://www.ncbi.nlm.nih.gov/nuccore/KC598087.1> |
| *Nannochloropsis* *gaditana* | NC_020014.1  KC012944.1 | https://www.ncbi.nlm.nih.gov/nuccore/NC_020014.1  <https://www.ncbi.nlm.nih.gov/nuccore/KC012944.1> |
| *Rhizochromulina* *marina* voucher A13,803 | MK561360.1 | <https://www.ncbi.nlm.nih.gov/nuccore/MK561360.1> |
| *Dictyocha* *speculum* CCMP1381 | MK561359.1 | <https://www.ncbi.nlm.nih.gov/nuccore/MK561359.1> |
| *Undaria* *pinnatifida* | NC_028503.1  KP298002.1 | https://www.ncbi.nlm.nih.gov/nuccore/NC_028503.1  <https://www.ncbi.nlm.nih.gov/nuccore/KP298002.1> |
| *Costaria costata* | NC_028502.1  KR336545.1 | https://www.ncbi.nlm.nih.gov/nuccore/NC_028502.1  <https://www.ncbi.nlm.nih.gov/nuccore/KR336545.1> |
| *Sargassum thunbergii* | NC_029134.1  KU500638.1 | https://www.ncbi.nlm.nih.gov/nuccore/NC_029134.1  <https://www.ncbi.nlm.nih.gov/nuccore/KU500638.1> |
| *Coccophora langsdorfii* | NC_032288.1  KU255795.1 | https://www.ncbi.nlm.nih.gov/nuccore/NC_032288.1  <https://www.ncbi.nlm.nih.gov/nuccore/KU255795.1> |
| *Pleurocladia* *lacustris* Sa2 | NC_032045.1  KU164871.1 | https://www.ncbi.nlm.nih.gov/nuccore/NC_032045.1  <https://www.ncbi.nlm.nih.gov/nuccore/KU164871.1> |
| *Pleurocladia* *lacustris* SAG 25.93 | KU164872.1 | <https://www.ncbi.nlm.nih.gov/nuccore/KU164872.1> |
| *Sargassum vachellianum* | KT188823.1 | <https://www.ncbi.nlm.nih.gov/nuccore/KT188823.1> |
| *Synura* *petersenii* S114.C7 | MH795128.1 | <https://www.ncbi.nlm.nih.gov/nuccore/MH795128.1> |
| *Heterosigma* *akashiwo* CCMP 452 | EU168191.1 | <https://www.ncbi.nlm.nih.gov/nuccore/EU168191.1> |
| *Heterosigma* *akashiwo* NIES 293 | EU168190.1 | <https://www.ncbi.nlm.nih.gov/nuccore/EU168190.1> |
| *Heterosigma* *akashiwo* CCMP1596 | LC269924.1 | <https://www.ncbi.nlm.nih.gov/nuccore/LC269924.1> |
| *Heterosigma* *akashiwo* HaFk01 | LC269923.1 | <https://www.ncbi.nlm.nih.gov/nuccore/LC269923.1> |
| *Heterosigma akashiwo* CCMP3374 | LC269922.1 | <https://www.ncbi.nlm.nih.gov/nuccore/LC269922.1> |
| *Heterosigma akashiwo* CCMP2274 | LC269921.1 | <https://www.ncbi.nlm.nih.gov/nuccore/LC269921.1> |
| *Heterosigma akashiwo* CCAP934/4 | LC269920.1 | <https://www.ncbi.nlm.nih.gov/nuccore/LC269920.1> |
| *Heterosigma akashiwo* EHUSP01 | LC269919.1 | <https://www.ncbi.nlm.nih.gov/nuccore/LC269919.1> |
| *Heterosigma akashiwo* CCAP934/8 | LC269918.1 | <https://www.ncbi.nlm.nih.gov/nuccore/LC269918.1> |
| *Heterosigma akashiwo* | NC_010772.1 | <https://www.ncbi.nlm.nih.gov/nuccore/NC_010772.1> |
| *Sargassum horneri* | NC_029856.1  KP881334.1 | https://www.ncbi.nlm.nih.gov/nuccore/NC_029856.1  <https://www.ncbi.nlm.nih.gov/nuccore/KP881334.1> |
| *Saccharina japonica* | NC_018523.1  JQ405663.1 | https://www.ncbi.nlm.nih.gov/nuccore/NC_018523.1  <https://www.ncbi.nlm.nih.gov/nuccore/JQ405663.1> |
| *Vaucheria litorea* | NC_011600.1  EU912438.1 | https://www.ncbi.nlm.nih.gov/nuccore/NC_011600.1  <https://www.ncbi.nlm.nih.gov/nuccore/EU912438.1> |
| *Fucus vesiculosus* var. spiralis UC 2050586 | MG922855.1 | <https://www.ncbi.nlm.nih.gov/nuccore/MG922855.1> |
| *Aureoumbra lagunensis* CCMP 1507 | GQ231542.1 | <https://www.ncbi.nlm.nih.gov/nuccore/GQ231542.1> |
| *Aureococcus anophagefferens* CCMP 1984 | GQ231541.1 | <https://www.ncbi.nlm.nih.gov/nuccore/GQ231541.1> |
| *Nannochloropsis salina* CCMP1776 | KJ410685.1 | <https://www.ncbi.nlm.nih.gov/nuccore/KJ410685.1> |
| *Nannochloropsis oceanica* strain LAMB2011 | CP038136.1 | <https://www.ncbi.nlm.nih.gov/nuccore/CP038136.1> |
| *Nannochloropsis granulata* | NC_022259.1  KC598085.1 | https://www.ncbi.nlm.nih.gov/nuccore/NC_022259.1  <https://www.ncbi.nlm.nih.gov/nuccore/KC598085.1> |
| *Nannochloropsis gaditana* CCMP1894 | CM009081.1 | <https://www.ncbi.nlm.nih.gov/nuccore/CM009081.1> |
| *Nannochloropsis gaditana* CCMP526 | KJ410682.1 | <https://www.ncbi.nlm.nih.gov/nuccore/KJ410682.1> |
| *Aureoumbra lagunensis* | NC_012903.1 | <https://www.ncbi.nlm.nih.gov/nuccore/NC_012903.1> |
| *Aureococcus anophagefferens* | NC_012898.1 | <https://www.ncbi.nlm.nih.gov/nuccore/NC_012898.1> |
| *Undaria pinnatifida* 474 | KU200463.1 | <https://www.ncbi.nlm.nih.gov/nuccore/KU200463.1> |
| *Endarachne binghamiae* | NC_038231.1  MF460360.1 | https://www.ncbi.nlm.nih.gov/nuccore/NC_038231.1  <https://www.ncbi.nlm.nih.gov/nuccore/MF460360.1> |
| *Mallomonas splendens* CCMP1782 | NC_040135.1  MH795131.1 | https://www.ncbi.nlm.nih.gov/nuccore/NC_040135.1  <https://www.ncbi.nlm.nih.gov/nuccore/MH795131.1> |
| *Neotessella volvocina* CCMP1781 | MH795132.1 | <https://www.ncbi.nlm.nih.gov/nuccore/MH795132.1> |
| *Synura uvella* FBCC200023 | NC_040134.1  MH795130.1 | https://www.ncbi.nlm.nih.gov/nuccore/NC_040134.1  <https://www.ncbi.nlm.nih.gov/nuccore/MH795130.1> |
| *Synura sphagnicola* FBCC200022 | MH795129.1 | <https://www.ncbi.nlm.nih.gov/nuccore/MH795129.1> |
| *Gracilaria changii* | NC_038051.1  KY018922.1 | https://www.ncbi.nlm.nih.gov/nuccore/NC_038051.1  <https://www.ncbi.nlm.nih.gov/nuccore/KY018922.1> |
| *Cyanidium caldarium* strain RK1 | AF022186.2 | <https://www.ncbi.nlm.nih.gov/nuccore/AF022186.2> |
| *Grateloupia taiwanensis* | NC_021618.1  KC894740.1 | https://www.ncbi.nlm.nih.gov/nuccore/NC_021618.1  <https://www.ncbi.nlm.nih.gov/nuccore/KC894740.1> |
| *Pleurostichidium falkenbergii* | NC_042794.1  MH853471.1 | https://www.ncbi.nlm.nih.gov/nuccore/NC_042794.1  <https://www.ncbi.nlm.nih.gov/nuccore/MH853471.1> |
| *Boldia erythrosiphon* strain UTEX LB2858 | NC_034776.1  KY709208.1 | https://www.ncbi.nlm.nih.gov/nuccore/NC_034776.1  <https://www.ncbi.nlm.nih.gov/nuccore/KY709208.1> |
| *Bangiopsis subsimplex* strain UTEX LB2854 | KY709207.1 | <https://www.ncbi.nlm.nih.gov/nuccore/KY709207.1> |
| *Bulboplastis apyrenoidosa* strain NIES-2742 | NC_034787.1  KY709209.1 | https://www.ncbi.nlm.nih.gov/nuccore/NC_034787.1  <https://www.ncbi.nlm.nih.gov/nuccore/KY709209.1> |
| *Choreocolax polysiphoniae* | NC_026522.1  KP308096.1 | https://www.ncbi.nlm.nih.gov/nuccore/NC_026522.1  <https://www.ncbi.nlm.nih.gov/nuccore/KP308096.1> |
| *Bangia fuscopurpurea* OUCPT01 | KP714733.1 | <https://www.ncbi.nlm.nih.gov/nuccore/KP714733.1> |
| *Pyropia endiviifolia* | KT716756.1 | <https://www.ncbi.nlm.nih.gov/nuccore/KT716756.1> |
| *Grateloupia filicina* | NC_037841.1  MG598531.1 | https://www.ncbi.nlm.nih.gov/nuccore/NC_037841.1  <https://www.ncbi.nlm.nih.gov/nuccore/MG598531.1> |
| *Corallina chilensis* voucher UC2050474 | MK598845.1  NC_042901.1 | https://www.ncbi.nlm.nih.gov/nuccore/MK598845.1  <https://www.ncbi.nlm.nih.gov/nuccore/NC_042901.1> |
| *Gracilariopsis lemaneiformis* voucher 475 | NC_029644.1  KU179794.1 | https://www.ncbi.nlm.nih.gov/nuccore/NC_029644.1  <https://www.ncbi.nlm.nih.gov/nuccore/KU179794.1> |
| *Kumanoa americana* | NC_031178.1  KX284725.1 | https://www.ncbi.nlm.nih.gov/nuccore/NC_031178.1  <https://www.ncbi.nlm.nih.gov/nuccore/KX284725.1> |
| *Palmaria palmata* | NC_031147.1  KX284726.1 | https://www.ncbi.nlm.nih.gov/nuccore/NC_031147.1  <https://www.ncbi.nlm.nih.gov/nuccore/KX284726.1> |
| *Thorea hispida* | NC_031171.1  KX284714.1 | https://www.ncbi.nlm.nih.gov/nuccore/NC_031171.1  <https://www.ncbi.nlm.nih.gov/nuccore/KX284714.1> |
| *Gelidium gabrielsonii* voucher UC2050580 | MG922860.1 | <https://www.ncbi.nlm.nih.gov/nuccore/MG922860.1> |
| *Gelidium gabrielsonii* isolate Upper intertidal crevice, Hopkins Marine Station, Pacific Grove, California voucher UC2050580 | NC_040156.1 | <https://www.ncbi.nlm.nih.gov/nuccore/NC_040156.1> |
| *Rhodymenia pseudopalmata* | NC_031144.1  KX284709.1 | https://www.ncbi.nlm.nih.gov/nuccore/NC_031144.1  <https://www.ncbi.nlm.nih.gov/nuccore/KX284709.1> |
| *Porphyridium sordidum* | NC_031175.1  KX284720.1 | https://www.ncbi.nlm.nih.gov/nuccore/NC_031175.1  <https://www.ncbi.nlm.nih.gov/nuccore/KX284720.1> |
| *Rhodochaete parvula* | NC_031180.2  KX284728.2 | https://www.ncbi.nlm.nih.gov/nuccore/NC_031180.2  <https://www.ncbi.nlm.nih.gov/nuccore/KX284728.2> |
| *Erythrotrichia carnea* | NC_031176.2  KX284721.2 | https://www.ncbi.nlm.nih.gov/nuccore/NC_031176.2  <https://www.ncbi.nlm.nih.gov/nuccore/KX284721.2> |
| *Bangiopsis subsimplex* | NC_031173.1  KX284718.1 | https://www.ncbi.nlm.nih.gov/nuccore/NC_031173.1  <https://www.ncbi.nlm.nih.gov/nuccore/KX284718.1> |
| *Schimmelmannia schousboei* | NC_031168.1  KX284711.1 | https://www.ncbi.nlm.nih.gov/nuccore/NC_031168.1  <https://www.ncbi.nlm.nih.gov/nuccore/KX284711.1> |
| *Ahnfeltia plicata* | NC_031145.1  KX284715.1 | https://www.ncbi.nlm.nih.gov/nuccore/NC_031145.1  <https://www.ncbi.nlm.nih.gov/nuccore/KX284715.1> |
| *Asparagopsis taxiformis* | NC_031148.1  KX284717.1 | https://www.ncbi.nlm.nih.gov/nuccore/NC_031148.1  <https://www.ncbi.nlm.nih.gov/nuccore/KX284717.1> |
| *Ceramium japonicum* | NC_031174.1  KX284719.1 | https://www.ncbi.nlm.nih.gov/nuccore/NC_031174.1  <https://www.ncbi.nlm.nih.gov/nuccore/KX284719.1> |
| *Gracilaria chorda* | NC_031149.1  KX284722.1 | https://www.ncbi.nlm.nih.gov/nuccore/NC_031149.1  <https://www.ncbi.nlm.nih.gov/nuccore/KX284722.1> |
| *Hildenbrandia rivularis* | NC_031177.1  KX284723.1 | https://www.ncbi.nlm.nih.gov/nuccore/NC_031177.1  <https://www.ncbi.nlm.nih.gov/nuccore/KX284723.1> |
| *Hildenbrandia rubra* | NC_031146.1  KX284724.1 | https://www.ncbi.nlm.nih.gov/nuccore/NC_031146.1  <https://www.ncbi.nlm.nih.gov/nuccore/KX284724.1> |
| *Apophlaea sinclairii* | NC_031172.1  KX284716.1 | https://www.ncbi.nlm.nih.gov/nuccore/NC_031172.1  <https://www.ncbi.nlm.nih.gov/nuccore/KX284716.1> |
| *Schizymenia dubyi* | NC_031169.1  KX284712.1 | https://www.ncbi.nlm.nih.gov/nuccore/NC_031169.1  <https://www.ncbi.nlm.nih.gov/nuccore/KX284712.1> |
| *Plocamium cartilagineum* | NC_031179.1  KX284727.1 | https://www.ncbi.nlm.nih.gov/nuccore/NC_031179.1  <https://www.ncbi.nlm.nih.gov/nuccore/KX284727.1> |
| *Riquetophycus* sp. | KX284710.1 | <https://www.ncbi.nlm.nih.gov/nuccore/KX284710.1> |
| *Sebdenia* *flabellata* | NC_031170.1  KX284713.1 | https://www.ncbi.nlm.nih.gov/nuccore/NC_031170.1  <https://www.ncbi.nlm.nih.gov/nuccore/KX284713.1> |
| *Compsopogon caeruleus* | NC_035350.1  KY083067.1 | https://www.ncbi.nlm.nih.gov/nuccore/NC_035350.1  <https://www.ncbi.nlm.nih.gov/nuccore/KY083067.1> |
| *Porphyra yezoensis* | AP006715.1 | <https://www.ncbi.nlm.nih.gov/nuccore/AP006715.1> |
| *Sheathia arcuata* | NC_035231.1  KY033529.1 | https://www.ncbi.nlm.nih.gov/nuccore/NC_035231.1  <https://www.ncbi.nlm.nih.gov/nuccore/KY033529.1> |
| *Neogoniolithon spectabile* | NC_039978.1  MH281628.1 | https://www.ncbi.nlm.nih.gov/nuccore/NC_039978.1  <https://www.ncbi.nlm.nih.gov/nuccore/MH281628.1> |
| *Lithothamnion* sp. | MH281627.1 | <https://www.ncbi.nlm.nih.gov/nuccore/MH281627.1> |
| *Synarthrophyton chejuense* | NC_039977.1  MH281626.1 | https://www.ncbi.nlm.nih.gov/nuccore/NC_039977.1  <https://www.ncbi.nlm.nih.gov/nuccore/MH281626.1> |
| *Rhodogorgon* sp. | MH281630.1 | <https://www.ncbi.nlm.nih.gov/nuccore/MH281630.1> |
| *Renouxia* sp. | MH281629.1 | <https://www.ncbi.nlm.nih.gov/nuccore/MH281629.1> |
| *Mastocarpus papillatus* voucher UC2050562 | NC_031167.1  KX525588.1 | https://www.ncbi.nlm.nih.gov/nuccore/NC_031167.1  <https://www.ncbi.nlm.nih.gov/nuccore/KX525588.1> |
| *Pyropia perforata* | NC_024050.1  KC904971.1 | https://www.ncbi.nlm.nih.gov/nuccore/NC_024050.1  <https://www.ncbi.nlm.nih.gov/nuccore/KC904971.1> |
| *Pyropia perforata* voucher VK-11-00061 | KF515973.1 | <https://www.ncbi.nlm.nih.gov/nuccore/KF515973.1> |
| *Pyropia perforata* voucher UC 807662 | KF515972.1 | <https://www.ncbi.nlm.nih.gov/nuccore/KF515972.1> |
| *Pyropia perforata* voucher UC 2019900 | KJ776833.1 | <https://www.ncbi.nlm.nih.gov/nuccore/KJ776833.1> |
| *Pyropia perforata* voucher UC 2019901 | KJ776834.1 | <https://www.ncbi.nlm.nih.gov/nuccore/KJ776834.1> |
| *Pyropia perforata* voucher UC 2019902 | KJ776835.1 | <https://www.ncbi.nlm.nih.gov/nuccore/KJ776835.1> |
| *Pyropia perforata* voucher LD-Ag 13031 | KJ776829.1 | <https://www.ncbi.nlm.nih.gov/nuccore/KJ776829.1> |
| *Pyropia perforata* voucher LD-Ag 13032 | KJ776831.1 | <https://www.ncbi.nlm.nih.gov/nuccore/KJ776831.1> |
| *Pyropia perforata* voucher LD-Ag 13038 | KJ776827.1 | <https://www.ncbi.nlm.nih.gov/nuccore/KJ776827.1> |
| *Pyropia perforata* voucher UC 1450590 | KJ776832.1 | <https://www.ncbi.nlm.nih.gov/nuccore/KJ776832.1> |
| *Pyropia perforata* voucher UC 95735 | KJ776830.1 | <https://www.ncbi.nlm.nih.gov/nuccore/KJ776830.1> |
| *Pyropia perforata* voucher UC 95739 | KJ776828.1 | https://www.ncbi.nlm.nih.gov/nuccore/KJ776828.1 https://www.ncbi.nlm.nih.gov/nuccore/ |
| *Porphyra umbilicalis* | NC_035573.1  MF385003.1 | https://www.ncbi.nlm.nih.gov/nuccore/NC_035573.1  <https://www.ncbi.nlm.nih.gov/nuccore/MF385003.1> |
| *Galdieria sulphuraria* strain 074W | NC_024665.1  KJ700459.1 | https://www.ncbi.nlm.nih.gov/nuccore/NC_024665.1  <https://www.ncbi.nlm.nih.gov/nuccore/KJ700459.1> |
| *Calliarthron tuberculosum* | NC_021075.1  KC153978.1 | https://www.ncbi.nlm.nih.gov/nuccore/NC_021075.1  <https://www.ncbi.nlm.nih.gov/nuccore/KC153978.1> |
| *Chondrus crispus* strain PCG | NC_020795.1  HF562234.1 | https://www.ncbi.nlm.nih.gov/nuccore/NC_020795.1  <https://www.ncbi.nlm.nih.gov/nuccore/HF562234.1> |
| *Cyanidioschyzon merolae* strain 10D | NC_004799.1 | <https://www.ncbi.nlm.nih.gov/nuccore/NC_004799.1> |
| *Pyropia haitanensis* voucher PH-38 | NC_021189.1  KC464603.1 | https://www.ncbi.nlm.nih.gov/nuccore/NC_021189.1  <https://www.ncbi.nlm.nih.gov/nuccore/KC464603.1> |
| *Pyropia yezoensis* | NC_007932.1  KC517072.1 | https://www.ncbi.nlm.nih.gov/nuccore/NC_007932.1  <https://www.ncbi.nlm.nih.gov/nuccore/KC517072.1> |
| *Kappaphycus alvarezii* | NC_036637.1  KU892652.1 | https://www.ncbi.nlm.nih.gov/nuccore/NC_036637.1  <https://www.ncbi.nlm.nih.gov/nuccore/KU892652.1> |
| *Porphyra purpurea* | NC_000925.1  U38804.1 | https://www.ncbi.nlm.nih.gov/nuccore/NC_000925.1  <https://www.ncbi.nlm.nih.gov/nuccore/U38804.1> |
| *Cyanidiaceae* sp. MX-AZ01 | KJ569775.1 | <https://www.ncbi.nlm.nih.gov/nuccore/KJ569775.1> |
| *Crassa firma* | KX601051.1 | <https://www.ncbi.nlm.nih.gov/nuccore/KX601051.1> |
| *Gracilaria firma* | NC_033877.1 | https://www.ncbi.nlm.nih.gov/nuccore/NC_033877.1 |
| *Gracilaria tenuistipitata* var. liui | NC_006137.1  AY673996.1 | https://www.ncbi.nlm.nih.gov/nuccore/NC_006137.1  <https://www.ncbi.nlm.nih.gov/nuccore/AY673996.1> |
| *Gracilaria chilensis* | MF401963.1 | <https://www.ncbi.nlm.nih.gov/nuccore/MF401963.1> |
| *Balbiania investiens* | NC_042171.1  MH026108.1 | https://www.ncbi.nlm.nih.gov/nuccore/NC_042171.1  https://www.ncbi.nlm.nih.gov/nuccore/MH026108.1 |
| *Membranoptera weeksiae* | NC_032396.1  KJ513670.1 | https://www.ncbi.nlm.nih.gov/nuccore/NC_032396.1  <https://www.ncbi.nlm.nih.gov/nuccore/KJ513670.1> |
| *Porphyridium purpureum* | NC_023133.1 | <https://www.ncbi.nlm.nih.gov/nuccore/NC_023133.1> |
| *Acrochaetium secundatum* | NC_042170.1  MH026107.1 | https://www.ncbi.nlm.nih.gov/nuccore/NC_042170.1  <https://www.ncbi.nlm.nih.gov/nuccore/MH026107.1> |
| *Chondria* sp. isolate PD1582 | MF101451.1 | <https://www.ncbi.nlm.nih.gov/nuccore/MF101451.1> |
| *Chondria* sp. isolate PD745 | MF101431.1 | <https://www.ncbi.nlm.nih.gov/nuccore/MF101431.1> |
| *Chondria* sp. isolate PD620 | MF101429.1 | <https://www.ncbi.nlm.nih.gov/nuccore/MF101429.1> |
| *Dasyclonium* *flaccidum* isolate PD1087 | NC_035287.1  MF101443.1 | https://www.ncbi.nlm.nih.gov/nuccore/NC_035287.1  https://www.ncbi.nlm.nih.gov/nuccore/MF101443.1 |
| *Dipterosiphonia* *australica* isolate PD1107 | NC_035288.1  MF101444.1 | https://www.ncbi.nlm.nih.gov/nuccore/NC_035288.1  <https://www.ncbi.nlm.nih.gov/nuccore/MF101444.1> |
| *Sonderella linearis* isolate PD1151 | NC_035289.1  MF101445.1 | https://www.ncbi.nlm.nih.gov/nuccore/NC_035289.1  <https://www.ncbi.nlm.nih.gov/nuccore/MF101445.1> |
| *Gredgaria maugeana* isolate PD1230 | NC_035290.1  MF101446.1 | https://www.ncbi.nlm.nih.gov/nuccore/NC_035290.1  <https://www.ncbi.nlm.nih.gov/nuccore/MF101446.1> |
| *Thaumatella adunca* isolate PD1388 | NC_035291.1  MF101447.1 | https://www.ncbi.nlm.nih.gov/nuccore/NC_035291.1  <https://www.ncbi.nlm.nih.gov/nuccore/MF101447.1> |
| *Lophocladia kuetzingii* isolate PD1509 | NC_035292.1  MF101448.1 | https://www.ncbi.nlm.nih.gov/nuccore/NC_035292.1  <https://www.ncbi.nlm.nih.gov/nuccore/MF101448.1> |
| *Kuetzingia canaliculata* isolate PD1540 | NC_035293.1  MF101449.1 | https://www.ncbi.nlm.nih.gov/nuccore/NC_035293.1  <https://www.ncbi.nlm.nih.gov/nuccore/MF101449.1> |
| *Tolypiocladia glomerulata* isolate PD1825 | NC_035299.1  MF101467.1 | https://www.ncbi.nlm.nih.gov/nuccore/NC_035299.1  <https://www.ncbi.nlm.nih.gov/nuccore/MF101467.1> |
| *Digenea simplex* isolate PD1820 | NC_035298.1  MF101465.1 | https://www.ncbi.nlm.nih.gov/nuccore/NC_035298.1  <https://www.ncbi.nlm.nih.gov/nuccore/MF101465.1> |
| *Polysiphonia* *schneideri* isolate PD1720 | NC_035296.1  MF101454.1 | https://www.ncbi.nlm.nih.gov/nuccore/NC_035296.1  <https://www.ncbi.nlm.nih.gov/nuccore/MF101454.1> |
| *Polysiphonia* *scopulorum* isolate PD899 | NC_035282.1  MF101438.1 | https://www.ncbi.nlm.nih.gov/nuccore/NC_035282.1  <https://www.ncbi.nlm.nih.gov/nuccore/MF101438.1> |
| *Polysiphonia* *stricta* isolate PD550 | NC_035275.1  MF101428.1 | https://www.ncbi.nlm.nih.gov/nuccore/NC_035275.1  <https://www.ncbi.nlm.nih.gov/nuccore/MF101428.1> |
| *Polysiphonia elongata* isolate PD547 | NC_035274.1  MF101427.1 | https://www.ncbi.nlm.nih.gov/nuccore/NC_035274.1  <https://www.ncbi.nlm.nih.gov/nuccore/MF101427.1> |
| *Polysiphonia infestans* isolate PD763 | NC_035277.1  MF101432.1 | https://www.ncbi.nlm.nih.gov/nuccore/NC_035277.1  <https://www.ncbi.nlm.nih.gov/nuccore/MF101432.1> |
| *Polysiphonia brodiei* isolate PD516 | NC_035272.1  MF101425.1 | https://www.ncbi.nlm.nih.gov/nuccore/NC_035272.1  <https://www.ncbi.nlm.nih.gov/nuccore/MF101425.1> |
| *Polysiphonia sertularioides* isolate PD001 | NC_035270.1  MF101423.1 | https://www.ncbi.nlm.nih.gov/nuccore/NC_035270.1  <https://www.ncbi.nlm.nih.gov/nuccore/MF101423.1> |
| *Polysiphonia sertularioides* isolate PD863 | MF101435.1 | <https://www.ncbi.nlm.nih.gov/nuccore/MF101435.1> |
| *Polysiphonia* sp. isolate PD1760 | MF101456.1 | <https://www.ncbi.nlm.nih.gov/nuccore/MF101456.1> |
| *Polysiphonia* sp. isolate JH1432 | MF101414.1 | <https://www.ncbi.nlm.nih.gov/nuccore/MF101414.1> |
| *Bryothamnion* *seaforthii* isolate PD644 | NC_035276.1  MF101430.1 | https://www.ncbi.nlm.nih.gov/nuccore/NC_035276.1  <https://www.ncbi.nlm.nih.gov/nuccore/MF101430.1> |
| *Cliftonaea pectinata* isolate PD1561 | NC_035294.1  MF101450.1 | https://www.ncbi.nlm.nih.gov/nuccore/NC_035294.1  <https://www.ncbi.nlm.nih.gov/nuccore/MF101450.1> |
| *Ophidocladus simpliciusculus* isolate PD949 | NC_035284.1  MF101440.1 | https://www.ncbi.nlm.nih.gov/nuccore/NC_035284.1  <https://www.ncbi.nlm.nih.gov/nuccore/MF101440.1> |
| *Vertebrata australis* isolate PD931 | NC_035283.1  MF101439.1 | https://www.ncbi.nlm.nih.gov/nuccore/NC_035283.1  <https://www.ncbi.nlm.nih.gov/nuccore/MF101439.1> |
| *Vertebrata isogona* isolate PD831 | NC_035278.1  MF101433.1 | https://www.ncbi.nlm.nih.gov/nuccore/NC_035278.1  <https://www.ncbi.nlm.nih.gov/nuccore/MF101433.1> |
| *Vertebrata thuyoides* isolate PD546 | NC_035273.1  MF101426.1 | https://www.ncbi.nlm.nih.gov/nuccore/NC_035273.1  <https://www.ncbi.nlm.nih.gov/nuccore/MF101426.1> |
| *Vertebrata lanosa* | NC_026523.1  KP308097.1 | https://www.ncbi.nlm.nih.gov/nuccore/NC_026523.1  <https://www.ncbi.nlm.nih.gov/nuccore/KP308097.1> |
| *Melanothamnus harveyi* isolate PD890 | NC_035281.1  MF101437.1 | https://www.ncbi.nlm.nih.gov/nuccore/NC_035281.1  <https://www.ncbi.nlm.nih.gov/nuccore/MF101437.1> |
| *Herposiphonia versicolor* isolate PD852 | NC_035279.1  MF101434.1 | https://www.ncbi.nlm.nih.gov/nuccore/NC_035279.1  <https://www.ncbi.nlm.nih.gov/nuccore/MF101434.1> |
| *Rhodomela confervoides* isolate PD508 | NC_035271.1  MF101424.1 | https://www.ncbi.nlm.nih.gov/nuccore/NC_035271.1  <https://www.ncbi.nlm.nih.gov/nuccore/MF101424.1> |
| *Bostrychia simpliciuscula* isolate JW3897 | NC_035268.1  MF101421.1 | https://www.ncbi.nlm.nih.gov/nuccore/NC_035268.1  <https://www.ncbi.nlm.nih.gov/nuccore/MF101421.1> |
| *Bostrychia moritziana* isolate JW3660 | NC_035266.1  MF101419.1 | https://www.ncbi.nlm.nih.gov/nuccore/NC_035266.1  <https://www.ncbi.nlm.nih.gov/nuccore/MF101419.1> |
| *Bostrychia tenella* isolate JW3079 | NC_035264.1  MF101417.1 | https://www.ncbi.nlm.nih.gov/nuccore/NC_035264.1  <https://www.ncbi.nlm.nih.gov/nuccore/MF101417.1> |
| *Symphyocladia dendroidea* isolate JW3780 | NC_035267.1  MF101420.1 | https://www.ncbi.nlm.nih.gov/nuccore/NC_035267.1  <https://www.ncbi.nlm.nih.gov/nuccore/MF101420.1> |
| *Osmundaria fimbriata* isolate JW2841 | NC_035262.1  MF101415.1 | https://www.ncbi.nlm.nih.gov/nuccore/NC_035262.1  <https://www.ncbi.nlm.nih.gov/nuccore/MF101415.1> |
| *Laurenciella marilzae* isolate HV1501 | NC_035259.1  MF101410.1 | https://www.ncbi.nlm.nih.gov/nuccore/NC_035259.1  <https://www.ncbi.nlm.nih.gov/nuccore/MF101410.1> |
| *Periphykon beckeri* isolate JH1427 | NC_035261.1  MF101413.1 | https://www.ncbi.nlm.nih.gov/nuccore/NC_035261.1  <https://www.ncbi.nlm.nih.gov/nuccore/MF101413.1> |
| *Dictyomenia sonderi* isolate PD1725 | NC_035297.1  MF101455.1 | https://www.ncbi.nlm.nih.gov/nuccore/NC_035297.1  <https://www.ncbi.nlm.nih.gov/nuccore/MF101455.1> |
| *Palisada* sp. isolate PD1686 | MF101453.1 | <https://www.ncbi.nlm.nih.gov/nuccore/MF101453.1> |
| *Laurencieae* sp. isolate JFC1711 | MF101412.1 | <https://www.ncbi.nlm.nih.gov/nuccore/MF101412.1> |
| *Taenioma* *perpusillum* isolate PD1676 | NC_035295.1  MF101452.1 | https://www.ncbi.nlm.nih.gov/nuccore/NC_035295.1  <https://www.ncbi.nlm.nih.gov/nuccore/MF101452.1> |
| *Thuretia quercifolia* isolate PD1024 | NC_035286.1  MF101442.1 | https://www.ncbi.nlm.nih.gov/nuccore/NC_035286.1  <https://www.ncbi.nlm.nih.gov/nuccore/MF101442.1> |
| *Acrosorium ciliolatum* isolate HV3939 | NC_035260.1  MF101411.1 | https://www.ncbi.nlm.nih.gov/nuccore/NC_035260.1  <https://www.ncbi.nlm.nih.gov/nuccore/MF101411.1> |
| *Platysiphonia delicata* isolate HV1445 | NC_035258.1  MF101409.1 | https://www.ncbi.nlm.nih.gov/nuccore/NC_035258.1  <https://www.ncbi.nlm.nih.gov/nuccore/MF101409.1> |
| *Dipterocladia arabiensis* isolate DHO101 | NC_035257.1  MF101408.1 | https://www.ncbi.nlm.nih.gov/nuccore/NC_035257.1  <https://www.ncbi.nlm.nih.gov/nuccore/MF101408.1> |
| *Membranoptera platyphylla* voucher AHFH:5795 | NC_032041.1  KT266849.1 | https://www.ncbi.nlm.nih.gov/nuccore/NC_032041.1  <https://www.ncbi.nlm.nih.gov/nuccore/KT266849.1> |
| *Membranoptera tenuis* voucher UC266439 | NC_032399.1  KP675983.1 | https://www.ncbi.nlm.nih.gov/nuccore/NC_032399.1  <https://www.ncbi.nlm.nih.gov/nuccore/KP675983.1> |
| *Dasya naccarioides* isolate PD888 | NC_035280.1  MF101436.1 | https://www.ncbi.nlm.nih.gov/nuccore/NC_035280.1  <https://www.ncbi.nlm.nih.gov/nuccore/MF101436.1> |
| *Dasya binghamiae* voucher UC2050572 | NC_031161.1  KX247284.1 | https://www.ncbi.nlm.nih.gov/nuccore/NC_031161.1  <https://www.ncbi.nlm.nih.gov/nuccore/KX247284.1> |
| *Caloglossa beccarii* isolate JW4523 | NC_035269.1  MF101422.1 | https://www.ncbi.nlm.nih.gov/nuccore/NC_035269.1  <https://www.ncbi.nlm.nih.gov/nuccore/MF101422.1> |
| *Caloglossa intermedia* isolate JW3535 | NC_035265.1  MF101418.1 | https://www.ncbi.nlm.nih.gov/nuccore/NC_035265.1  <https://www.ncbi.nlm.nih.gov/nuccore/MF101418.1> |
| *Caloglossa monosticha* isolate JW3046 | NC_035263.1  MF101416.1 | https://www.ncbi.nlm.nih.gov/nuccore/NC_035263.1  <https://www.ncbi.nlm.nih.gov/nuccore/MF101416.1> |
| *Ceramium sungminbooi* voucher UC2050592 | NC_031211.1  KR025491.1 | https://www.ncbi.nlm.nih.gov/nuccore/NC_031211.1  <https://www.ncbi.nlm.nih.gov/nuccore/KR025491.1> |
| *Ceramium sungminbooi* | KR814486.1 | <https://www.ncbi.nlm.nih.gov/nuccore/KR814486.1> |
| *Spyridia filamentosa* isolate PD1020 | NC_035285.1  MF101441.1 | https://www.ncbi.nlm.nih.gov/nuccore/NC_035285.1  <https://www.ncbi.nlm.nih.gov/nuccore/MF101441.1> |
| *Corallina ferreyrae* voucher UC 1404138 | NC_041636.1 | <https://www.ncbi.nlm.nih.gov/nuccore/NC_041636.1> |
| *Porolithon onkodes* voucher TRH A26-1494 | NC_038144.1  KY212106.1 | https://www.ncbi.nlm.nih.gov/nuccore/NC_038144.1  <https://www.ncbi.nlm.nih.gov/nuccore/KY212106.1> |
| *Gracilariopsis heteroclada* | NC_038100.1  MF372957.1 | https://www.ncbi.nlm.nih.gov/nuccore/NC_038100.1  <https://www.ncbi.nlm.nih.gov/nuccore/MF372957.1> |
| *Gracilariopsis mclachlanii* | NC_039144.1 | <https://www.ncbi.nlm.nih.gov/nuccore/NC_039144.1> |
| *Gracilariopsis longissima* | NC_039143.1  MH396014.1 | https://www.ncbi.nlm.nih.gov/nuccore/NC_039143.1  <https://www.ncbi.nlm.nih.gov/nuccore/MH396014.1> |
| *Gracilariopsis lemaneiformis* | KP330491.1 | <https://www.ncbi.nlm.nih.gov/nuccore/KP330491.1> |
| *Gracilaria caudata* voucher SPF:57390 | NC_039139.1  MH396009.1 | https://www.ncbi.nlm.nih.gov/nuccore/NC_039139.1  <https://www.ncbi.nlm.nih.gov/nuccore/MH396009.1> |
| *Gracilaria salicornia* | NC_023785.1  KF861575.1 | https://www.ncbi.nlm.nih.gov/nuccore/NC_023785.1  <https://www.ncbi.nlm.nih.gov/nuccore/KF861575.1> |
| *Gracilaria chilensis* | KT266788.1 | <https://www.ncbi.nlm.nih.gov/nuccore/KT266788.1> |
| *Gracilaria vermiculophylla* | NC_039092.1  MH396013.1 | https://www.ncbi.nlm.nih.gov/nuccore/NC_039092.1  <https://www.ncbi.nlm.nih.gov/nuccore/MH396013.1> |
| *Gracilaria gracilis* voucher SPF55734 | NC_039141.1  MH396011.1 | https://www.ncbi.nlm.nih.gov/nuccore/NC_039141.1  <https://www.ncbi.nlm.nih.gov/nuccore/MH396011.1> |
| *Gracilaria ferox* voucher SPF:26055 | NC_039140.1  MH396010.1 | https://www.ncbi.nlm.nih.gov/nuccore/NC_039140.1  <https://www.ncbi.nlm.nih.gov/nuccore/MH396010.1> |
| *Hydropuntia rangiferina* voucher SPF:56055 | NC_039142.1  MH396012.1 | https://www.ncbi.nlm.nih.gov/nuccore/NC_039142.1  <https://www.ncbi.nlm.nih.gov/nuccore/MH396012.1> |
| *Melanthalia intermedia* | NC_039145.1  MH396016.1 | https://www.ncbi.nlm.nih.gov/nuccore/NC_039145.1  <https://www.ncbi.nlm.nih.gov/nuccore/MH396016.1> |
| *Gelidium vagum* | NC_029859.1  KT266787.1 | https://www.ncbi.nlm.nih.gov/nuccore/NC_029859.1  <https://www.ncbi.nlm.nih.gov/nuccore/KT266787.1> |
| *Gelidium elegans* | NC_029858.1  KT266786.1 | https://www.ncbi.nlm.nih.gov/nuccore/NC_029858.1  <https://www.ncbi.nlm.nih.gov/nuccore/KT266786.1> |
| *Gelidium sinicola* voucher UC276620 | NC_041175.1  MG922862.1 | https://www.ncbi.nlm.nih.gov/nuccore/NC_041175.1  <https://www.ncbi.nlm.nih.gov/nuccore/MG922862.1> |
| *Gelidium galapagense* voucher UC1884224 | NC_041174.1  MG922861.1 | https://www.ncbi.nlm.nih.gov/nuccore/NC_041174.1  <https://www.ncbi.nlm.nih.gov/nuccore/MG922861.1> |
| *Gelidium coulteri* voucher UC2050582 | NC_041173.1  MG922858.1 | https://www.ncbi.nlm.nih.gov/nuccore/NC_041173.1  <https://www.ncbi.nlm.nih.gov/nuccore/MG922858.1> |
| *Gelidium kathyanniae* voucher UC2050581 | NC_040158.1  MG922864.1 | https://www.ncbi.nlm.nih.gov/nuccore/NC_040158.1  <https://www.ncbi.nlm.nih.gov/nuccore/MG922864.1> |
| *Nemalion* sp. H.1444 | LT622871.1 | <https://www.ncbi.nlm.nih.gov/nuccore/LT622871.1> |
| *Dermonema virens* isolate J.0258 | NC_031655.1  LT622863.1 | https://www.ncbi.nlm.nih.gov/nuccore/NC_031655.1  <https://www.ncbi.nlm.nih.gov/nuccore/LT622863.1> |
| *Helminthora furcellata* isolate J.0165 | NC_031654.1  LT622876.1 | https://www.ncbi.nlm.nih.gov/nuccore/NC_031654.1  <https://www.ncbi.nlm.nih.gov/nuccore/LT622876.1> |
| *Helminthora furcellata* isolate J.0165 | LT622862.1 | <https://www.ncbi.nlm.nih.gov/nuccore/LT622862.1> |
| *Trichogloeopsis pedicelleta*  isolate C.0024 | NC_031668.1  LT622878.1 | https://www.ncbi.nlm.nih.gov/nuccore/NC_031668.1  <https://www.ncbi.nlm.nih.gov/nuccore/LT622878.1> |
| *Liagora brachyclada* isolate J.0126 | NC_031667.1  LT622877.1 | https://www.ncbi.nlm.nih.gov/nuccore/NC_031667.1  <https://www.ncbi.nlm.nih.gov/nuccore/LT622877.1> |
| *Liagora harveyana* | NC_031661.1  LT622869.1 | https://www.ncbi.nlm.nih.gov/nuccore/NC_031661.1  <https://www.ncbi.nlm.nih.gov/nuccore/LT622869.1> |
| *Titanophycus setchellii*  isolate J.0604 | NC_031665.1  LT622874.1 | https://www.ncbi.nlm.nih.gov/nuccore/NC_031665.1  <https://www.ncbi.nlm.nih.gov/nuccore/LT622874.1> |
| *Neoizziella asiatica* isolate J.0154 | NC_031663.1  LT622872.1 | https://www.ncbi.nlm.nih.gov/nuccore/NC_031663.1  <https://www.ncbi.nlm.nih.gov/nuccore/LT622872.1> |
| *Izziella formosana* isolate J.0158 | NC_031660.1  LT622868.1 | https://www.ncbi.nlm.nih.gov/nuccore/NC_031660.1  <https://www.ncbi.nlm.nih.gov/nuccore/LT622868.1> |
| *Hommersandiophycus borowitzkae* isolate HV00480 | NC_031659.1  LT622867.1 | https://www.ncbi.nlm.nih.gov/nuccore/NC_031659.1  <https://www.ncbi.nlm.nih.gov/nuccore/LT622867.1> |
| *Helminthocladia australis* isolate J.0167 | NC_031658.1  LT622866.1 | https://www.ncbi.nlm.nih.gov/nuccore/NC_031658.1  <https://www.ncbi.nlm.nih.gov/nuccore/LT622866.1> |
| *Galaxaura rugosa* isolate JFC0074 | NC_031657.1  LT622865.1 | https://www.ncbi.nlm.nih.gov/nuccore/NC_031657.1  <https://www.ncbi.nlm.nih.gov/nuccore/LT622865.1> |
| *Dichotomaria marginata* isolate HV04060 | NC_031656.1  LT622864.1 | https://www.ncbi.nlm.nih.gov/nuccore/NC_031656.1  <https://www.ncbi.nlm.nih.gov/nuccore/LT622864.1> |
| *Liagoropsis maxima*  isolate J.0256 | NC_031662.1  LT622870.1 | https://www.ncbi.nlm.nih.gov/nuccore/NC_031662.1  <https://www.ncbi.nlm.nih.gov/nuccore/LT622870.1> |
| *Scinaia undulata* isolate J.0081 | NC_031664.1  LT622873.1 | https://www.ncbi.nlm.nih.gov/nuccore/NC_031664.1  <https://www.ncbi.nlm.nih.gov/nuccore/LT622873.1> |
| *Yamadaella caenomyce* isolate J.0255 | NC_031666.1  LT622875.1 | https://www.ncbi.nlm.nih.gov/nuccore/NC_031666.1  <https://www.ncbi.nlm.nih.gov/nuccore/LT622875.1> |
| *Palmaria palmata* chloroplast DNA | AB807662.1 | <https://www.ncbi.nlm.nih.gov/nuccore/AB807662.1> |
| *Coeloseira compressa* | NC_030338.1  KU053957.1 | https://www.ncbi.nlm.nih.gov/nuccore/NC_030338.1  <https://www.ncbi.nlm.nih.gov/nuccore/KU053957.1> |
| *Sporolithon durum* | NC_029857.1  KT266785.1 | https://www.ncbi.nlm.nih.gov/nuccore/NC_029857.1  <https://www.ncbi.nlm.nih.gov/nuccore/KT266785.1> |
| *Porphyra pulchra* | NC_029861.1  KT266789.1 | https://www.ncbi.nlm.nih.gov/nuccore/NC_029861.1  <https://www.ncbi.nlm.nih.gov/nuccore/KT266789.1> |
| *Wildemania schizophylla* voucher UC 306400 | NC_029576.1  KR020505.1 | https://www.ncbi.nlm.nih.gov/nuccore/NC_029576.1  <https://www.ncbi.nlm.nih.gov/nuccore/KR020505.1> |
| *Porphyridium purpureum* culture CCMP:1328 | MF401423.1 | <https://www.ncbi.nlm.nih.gov/nuccore/MF401423.1> |
| *Cyanidium caldarium* | NC_001840.1 | <https://www.ncbi.nlm.nih.gov/nuccore/NC_001840.1> |
| *Palmaria decipiens* | MN967052 | <https://www.ncbi.nlm.nih.gov/nuccore/MN967052> |
| *Pyropia dentata* | LC521919 | <https://www.ncbi.nlm.nih.gov/nuccore/LC521919> |
| *Gracilaria textorii* | MN053320 | <https://www.ncbi.nlm.nih.gov/nuccore/MN053320> |
| *Gracilaria spinulosa* | MN053319 | <https://www.ncbi.nlm.nih.gov/nuccore/MN053319> |
| *Gracilaria edulis* | MN053318 | <https://www.ncbi.nlm.nih.gov/nuccore/MN053318> |
| *Grateloupia turuturu* | MN853877 | <https://www.ncbi.nlm.nih.gov/nuccore/MN853877> |
| *Caulerpa sertularioides* f. *longipes* | MK792750 | <https://www.ncbi.nlm.nih.gov/nuccore/MK792750> |
| *Caulerpa serrulata* | MK792749 | <https://www.ncbi.nlm.nih.gov/nuccore/MK792749> |
| *Micractinium pusillum* strain CCAP 232/1 | MN649872 | <https://www.ncbi.nlm.nih.gov/nuccore/MN649872> |
| *Auxenochlorella pyrenoidosa* | MN128434 | <https://www.ncbi.nlm.nih.gov/nuccore/MN128434> |
| *Caulerpa ashmeadii* voucher TS1851 | NC_045914  MH745228 | https://www.ncbi.nlm.nih.gov/nuccore/NC_045914  <https://www.ncbi.nlm.nih.gov/nuccore/MH745228> |
| *Codium fragile* | MN853876 | <https://www.ncbi.nlm.nih.gov/nuccore/MN853876> |
| Ulva pertusa | MN853875 | <https://www.ncbi.nlm.nih.gov/nuccore/MN853875> |
| *Chlorella vulgaris* strain UTEX259 | MK948102 | <https://www.ncbi.nlm.nih.gov/nuccore/MK948102> |
| *Chlorella vulgaris* strain NJ-7 | MK948100 | <https://www.ncbi.nlm.nih.gov/nuccore/MK948100> |
| *Asterarcys* sp. GP-2019 | MK995333 | <https://www.ncbi.nlm.nih.gov/nuccore/MK995333> |
| *Microrhizoidea pickettheapsiorum* voucher 5FNG | NC_044704  MN056173 | https://www.ncbi.nlm.nih.gov/nuccore/NC_044704  <https://www.ncbi.nlm.nih.gov/nuccore/MN056173> |
| *Chlorosarcinopsis eremi* | MN102114 | <https://www.ncbi.nlm.nih.gov/nuccore/MN102114> |
| *Chlorella sorokiniana* BD08 | CM017271 | <https://www.ncbi.nlm.nih.gov/nuccore/CM017271> |
| *Chlorella* sp. Dachan | CM017270 | <https://www.ncbi.nlm.nih.gov/nuccore/CM017270> |
| *Colemanosphaera angeleri* | NC_045893  MH511734 | https://www.ncbi.nlm.nih.gov/nuccore/NC_045893  <https://www.ncbi.nlm.nih.gov/nuccore/MH511734> |
| *Colemanosphaera charkowiensis* | MH511733 | <https://www.ncbi.nlm.nih.gov/nuccore/MH511733> |
| *Pediastrum duplex* | MK895950 | <https://www.ncbi.nlm.nih.gov/nuccore/MK895950> |
| *Sargassum fusiforme* | MN121852 | <https://www.ncbi.nlm.nih.gov/nuccore/MN121852> |
| *Vischeria stellata* strain SAG 33.83 | MK212028 | <https://www.ncbi.nlm.nih.gov/nuccore/MK212028> |
| *Laminaria solidungula* | NC_044690  MH784528 | https://www.ncbi.nlm.nih.gov/nuccore/NC_044690  <https://www.ncbi.nlm.nih.gov/nuccore/MH784528> |
| *Laminaria digitata* | NC_044689  MH784527 | https://www.ncbi.nlm.nih.gov/nuccore/NC_044689  <https://www.ncbi.nlm.nih.gov/nuccore/MH784527> |
| *Pseudopedinella elastica* voucher CCMP716 | NC_044408  MK518353 | https://www.ncbi.nlm.nih.gov/nuccore/NC_044408  <https://www.ncbi.nlm.nih.gov/nuccore/MK518353> |
| *Florenciella parvula* voucher CCMP2471 | NC_044407  MK518352 | https://www.ncbi.nlm.nih.gov/nuccore/NC_044407  <https://www.ncbi.nlm.nih.gov/nuccore/MK518352> |
| *Lessonia spicata* voucher DBM0003 | NC_044182 | <https://www.ncbi.nlm.nih.gov/nuccore/NC_044182> |
| *Cladosiphon okamuranus* | NC_046005  MG739403 | https://www.ncbi.nlm.nih.gov/nuccore/NC_046005  <https://www.ncbi.nlm.nih.gov/nuccore/MG739403> |
| *Ectocarpus siliculosus* | MN181444 | <https://www.ncbi.nlm.nih.gov/nuccore/MN181444> |
| *Scytosiphon promiscuus* | MK107985 | <https://www.ncbi.nlm.nih.gov/nuccore/MK107985> |
| *Scytosiphon promiscuus* | MK107984 | <https://www.ncbi.nlm.nih.gov/nuccore/MK107984> |
| *Scytosiphon canaliculatus* | NC_044758  MF591718 | https://www.ncbi.nlm.nih.gov/nuccore/NC_044758  <https://www.ncbi.nlm.nih.gov/nuccore/MF591718> |
| *Cryptomonas* sp. SAG 977-2f | LC484194 | <https://www.ncbi.nlm.nih.gov/nuccore/LC484194> |
| *Cryptomonas* sp. CCAC 1634B | LC484193 | <https://www.ncbi.nlm.nih.gov/nuccore/LC484193> |
| *Cryptomonas* *curvata* CCAP979/52 | LC484192 | <https://www.ncbi.nlm.nih.gov/nuccore/LC484192> |
| *Nitzschia* sp. strain Nitz4 | MG273660 | <https://www.ncbi.nlm.nih.gov/nuccore/MG273660> |
| *Nanofrustulum shiloi* strain SZCZM404 | MN276191 | <https://www.ncbi.nlm.nih.gov/nuccore/MN276191> |
| *Nitzschia alba* | NC_044785  MN065498 | https://www.ncbi.nlm.nih.gov/nuccore/NC_044785  <https://www.ncbi.nlm.nih.gov/nuccore/MN065498> |
| *Fragilariopsis cylindrus* | NC_045244  MK217824 | https://www.ncbi.nlm.nih.gov/nuccore/NC_045244  <https://www.ncbi.nlm.nih.gov/nuccore/MK217824> |
| *Skeletonema pseudocostatum* | MK372941 | <https://www.ncbi.nlm.nih.gov/nuccore/MK372941> |
| *Phaeodactylum tricornutum* strain ICE-H | MN937452 | <https://www.ncbi.nlm.nih.gov/nuccore/MN937452> |
| *Haslea nusantara* | NC_044491  MH681881 | https://www.ncbi.nlm.nih.gov/nuccore/NC_044491  <https://www.ncbi.nlm.nih.gov/nuccore/MH681881> |
| *Halamphora coffeaeformis* | NC_044465  MK045452 | https://www.ncbi.nlm.nih.gov/nuccore/NC_044465  <https://www.ncbi.nlm.nih.gov/nuccore/MK045452> |
| *Halamphora calidilacuna* | NC_044464  MK045451 | https://www.ncbi.nlm.nih.gov/nuccore/NC_044464  <https://www.ncbi.nlm.nih.gov/nuccore/MK045451> |
| *Halamphora americana* | NC_044463  MK045450 | https://www.ncbi.nlm.nih.gov/nuccore/NC_044463  <https://www.ncbi.nlm.nih.gov/nuccore/MK045450> |

# Table S 2. The mitochondrial genome data from the public database

| Species | accession  number | Raw data source |
| --- | --- | --- |
| *Didymosphenia geminata* | NC_032171.1  KX889125.1 | https://www.ncbi.nlm.nih.gov/nuccore/NC_032171.1  <https://www.ncbi.nlm.nih.gov/nuccore/KX889125.1> |
| *Pseudo-nitzschia multiseries* | NC_027265.1  KR149143.1 | https://www.ncbi.nlm.nih.gov/nuccore/NC_027265.1  <https://www.ncbi.nlm.nih.gov/nuccore/KR149143.1> |
| *Nitzschia palea* | MH297491.1 | <https://www.ncbi.nlm.nih.gov/nuccore/MH297491.1> |
| *Berkeleya fennica* | NC_026126.1  KM886611.1 | https://www.ncbi.nlm.nih.gov/nuccore/NC_026126.1  <https://www.ncbi.nlm.nih.gov/nuccore/KM886611.1> |
| *Toxarium undulatum* strain ECT3802 | NC_037988.1  MG271847.1 | https://www.ncbi.nlm.nih.gov/nuccore/NC_037988.1  <https://www.ncbi.nlm.nih.gov/nuccore/MG271847.1> |
| *Nitzschia* sp. strain Nitz4 | NC_037990.1  MG182051.1 | https://www.ncbi.nlm.nih.gov/nuccore/NC_037990.1  <https://www.ncbi.nlm.nih.gov/nuccore/MG182051.1> |
| *Cylindrotheca* *closterium* strain CCMP1855 | NC_037986.1  MG271845.1 | https://www.ncbi.nlm.nih.gov/nuccore/NC_037986.1  <https://www.ncbi.nlm.nih.gov/nuccore/MG271845.1> |
| Psammoneis *japonica* | NC_037989.1  MG148339.1 | https://www.ncbi.nlm.nih.gov/nuccore/NC_037989.1  <https://www.ncbi.nlm.nih.gov/nuccore/MG148339.1> |
| *Eunotia naegelii* strain UTEX FD354 | NC_037987.1  MG271846.1 | https://www.ncbi.nlm.nih.gov/nuccore/NC_037987.1  <https://www.ncbi.nlm.nih.gov/nuccore/MG271846.1> |
| *Phaeodactylum tricornutum* | NC_016739.1  HQ840789.1 | https://www.ncbi.nlm.nih.gov/nuccore/NC_016739.1  <https://www.ncbi.nlm.nih.gov/nuccore/HQ840789.1> |
| *Synedra acus* | NC_013710.1  GU002153.1 | https://www.ncbi.nlm.nih.gov/nuccore/NC_013710.1  <https://www.ncbi.nlm.nih.gov/nuccore/GU002153.1> |
| *Asterionella formosa* strain BGM1 | NC_032029.1  KY021079.1 | https://www.ncbi.nlm.nih.gov/nuccore/NC_032029.1  <https://www.ncbi.nlm.nih.gov/nuccore/KY021079.1> |
| *Skeletonema marinoi* voucher 06.JK029.Smar.mt | NC_028615.1  KT874463.1 | https://www.ncbi.nlm.nih.gov/nuccore/NC_028615.1  <https://www.ncbi.nlm.nih.gov/nuccore/KT874463.1> |
| *Fistulifera solaris* | NC_027978.1  KT363689.1 | https://www.ncbi.nlm.nih.gov/nuccore/NC_027978.1  <https://www.ncbi.nlm.nih.gov/nuccore/KT363689.1> |
| *Navicula ramosissima* voucher 10.TA439.Nram.mt | NC_031848.1  KX343079.1 | https://www.ncbi.nlm.nih.gov/nuccore/NC_031848.1  <https://www.ncbi.nlm.nih.gov/nuccore/KX343079.1> |
| *Proschkinia* sp. SZCZR1824 | MH800316.1 | <https://www.ncbi.nlm.nih.gov/nuccore/MH800316.1> |
| *Melosira undulata* | NC_037728.1  MF997421.1 | https://www.ncbi.nlm.nih.gov/nuccore/NC_037728.1  <https://www.ncbi.nlm.nih.gov/nuccore/MF997421.1> |
| *Entomoneis* sp. | MF997419.1 | <https://www.ncbi.nlm.nih.gov/nuccore/MF997419.1> |
| *Surirella* sp. | MF997423.1 | <https://www.ncbi.nlm.nih.gov/nuccore/MF997423.1> |
| *Halamphora* *coffeaeformis* | NC_037727.1  MF997420.1 | https://www.ncbi.nlm.nih.gov/nuccore/NC_037727.1  <https://www.ncbi.nlm.nih.gov/nuccore/MF997420.1> |
| *Thalassiosira pseudonana* | NC_007405.1  DQ186202.1 | https://www.ncbi.nlm.nih.gov/nuccore/NC_007405.1  <https://www.ncbi.nlm.nih.gov/nuccore/DQ186202.1> |
| *Nitzschia alba* | NC_037729.1  MF997422.1 | https://www.ncbi.nlm.nih.gov/nuccore/NC_037729.1  <https://www.ncbi.nlm.nih.gov/nuccore/MF997422.1> |
| *Nitzschia* sp. NIES-3581 | AP018510.1 | <https://www.ncbi.nlm.nih.gov/nuccore/AP018510.1> |
| *Nitzschia* sp. NIES-3576 | AP018509.1 | <https://www.ncbi.nlm.nih.gov/nuccore/AP018509.1> |
| *Nitzschia* sp. PL1-4 | AP018507.1 | <https://www.ncbi.nlm.nih.gov/nuccore/AP018507.1> |
| *Halamphora* sp. CSP-2018a | MF997424.1 | <https://www.ncbi.nlm.nih.gov/nuccore/MF997424.1> |
| *Haslea nusantara* | NC_044492.1 | <https://www.ncbi.nlm.nih.gov/nuccore/NC_044492.1> |
| *Lotharella oceanica* strain CCMP622 | NC_029731.1 | <https://www.ncbi.nlm.nih.gov/nuccore/NC_029731.1> |
| *Spongospora subterranea* | NC_034004.1 | <https://www.ncbi.nlm.nih.gov/nuccore/NC_034004.1> |
| *Roya obtusa* culture-collection SAG:168.80 | NC_022863.1 | <https://www.ncbi.nlm.nih.gov/nuccore/NC_022863.1> |
| *Closterium baillyanum* culture-collection SAG:50.89 | NC_022860.1  KF060940.1 | https://www.ncbi.nlm.nih.gov/nuccore/NC_022860.1  <https://www.ncbi.nlm.nih.gov/nuccore/KF060940.1> |
| *Entransia fimbriata* culture-collection UTEXLB 2353 | NC_022861.1  KF060941.1 | https://www.ncbi.nlm.nih.gov/nuccore/NC_022861.1  <https://www.ncbi.nlm.nih.gov/nuccore/KF060941.1> |
| *Chara vulgaris* | NC_005255.1  AY267353.1 | https://www.ncbi.nlm.nih.gov/nuccore/NC_005255.1  <https://www.ncbi.nlm.nih.gov/nuccore/AY267353.1> |
| *Mesostigma viride* | NC_008240.1 | <https://www.ncbi.nlm.nih.gov/nuccore/NC_008240.1> |
| *Chaetosphaeridium globosum* | NC_004118.1 | <https://www.ncbi.nlm.nih.gov/nuccore/NC_004118.1> |
| *Chaetosphaeridium globosum* mitochondrial DNA | AF494279.1 | <https://www.ncbi.nlm.nih.gov/nuccore/AF494279.1> |
| *Mesostigma viride* | AF353999.1 | <https://www.ncbi.nlm.nih.gov/nuccore/AF353999.1> |
| *Chlorokybus atmophyticus* | NC_009630.1 | <https://www.ncbi.nlm.nih.gov/nuccore/NC_009630.1> |
| *Chlorokybus atmophyticus* strain SAG 48.80 | EF463011.1 | <https://www.ncbi.nlm.nih.gov/nuccore/EF463011.1> |
| *Nitella hyalina* | NC_017598.1  JF810595.1 | https://www.ncbi.nlm.nih.gov/nuccore/NC_017598.1  https://www.ncbi.nlm.nih.gov/nuccore/JF810595.1 |
| *Mychonastes homosphaera* strain CAUP H6502 | NC_024760.1 | <https://www.ncbi.nlm.nih.gov/nuccore/NC_024760.1> |
| *Microspora stagnorum* culture-collection SAG:51.86 | NC_022862.1  KF060942.1 | https://www.ncbi.nlm.nih.gov/nuccore/NC_022862.1  <https://www.ncbi.nlm.nih.gov/nuccore/KF060942.1> |
| *Chromochloris zofingiensis* strain UTEX 56 | NC_024758.1  KJ806268.1 | https://www.ncbi.nlm.nih.gov/nuccore/NC_024758.1  <https://www.ncbi.nlm.nih.gov/nuccore/KJ806268.1> |
| *Kirchneriella aperta* strain SAG 2004 | KJ806269.1 | <https://www.ncbi.nlm.nih.gov/nuccore/KJ806269.1> |
| *Hariotina* sp. MMOGRB0030F | KU145405.1 | <https://www.ncbi.nlm.nih.gov/nuccore/KU145405.1> |
| *Scenedesmus obliquus* | X17375.2 | https://www.ncbi.nlm.nih.gov/nuccore/X17375.2 |
| *Scenedesmus obliquus* strain UTEX 78 | AF204057.1 | <https://www.ncbi.nlm.nih.gov/nuccore/AF204057.1> |
| *Tetradesmus obliquus* | KX756228.1 | <https://www.ncbi.nlm.nih.gov/nuccore/KX756228.1> |
| *Tetradesmus obliquus* strain KS3-2 | NC_002254.1 | <https://www.ncbi.nlm.nih.gov/nuccore/NC_002254.1> |
| *Tetradesmus obliquus* strain DOE152z | CM007918.1 | <https://www.ncbi.nlm.nih.gov/nuccore/CM007918.1> |
| *Pectinodesmus pectinatus* | NC_036659.1  KT946995.1 | https://www.ncbi.nlm.nih.gov/nuccore/NC_036659.1  <https://www.ncbi.nlm.nih.gov/nuccore/KT946995.1> |
| *Sphaeropleales* sp. YC001 | KT259054.1 | <https://www.ncbi.nlm.nih.gov/nuccore/KT259054.1> |
| *Bracteacoccus* *aeriusstrain* UTEX 1250 | NC_024755.1  KJ806265.1 | https://www.ncbi.nlm.nih.gov/nuccore/NC_024755.1  <https://www.ncbi.nlm.nih.gov/nuccore/KJ806265.1> |
| *Bracteacoccus* *minor* strain UTEX B66 | NC_024756.1  KJ806266.1 | https://www.ncbi.nlm.nih.gov/nuccore/NC_024756.1  <https://www.ncbi.nlm.nih.gov/nuccore/KJ806266.1> |
| *Neochloris aquatica* strain UTEX 138 | NC_024761.1  KJ806271.1 | https://www.ncbi.nlm.nih.gov/nuccore/NC_024761.1  <https://www.ncbi.nlm.nih.gov/nuccore/KJ806271.1> |
| *Chlorotetraedron incus* strain SAG 43.81 | NC_024757.1  KJ806267.1 | https://www.ncbi.nlm.nih.gov/nuccore/NC_024757.1  <https://www.ncbi.nlm.nih.gov/nuccore/KJ806267.1> |
| *Gonium pectorale* | NC_020437.1 | <https://www.ncbi.nlm.nih.gov/nuccore/NC_020437.1> |
| *Gonium pectorale* isolate: K3-F3-4 | AP012493.1 | <https://www.ncbi.nlm.nih.gov/nuccore/AP012493.1> |
| *Yamagishiella unicocca* strain NIES-3982 | NC_033969.1  KY442293.1 | https://www.ncbi.nlm.nih.gov/nuccore/NC_033969.1  <https://www.ncbi.nlm.nih.gov/nuccore/KY442293.1> |
| *Eudorina* sp. 2006-703-Eu-15 | KY442294.1 | <https://www.ncbi.nlm.nih.gov/nuccore/KY442294.1> |
| *Pleodorina starrii* | NC_021108.1  JX977845.1 | https://www.ncbi.nlm.nih.gov/nuccore/NC_021108.1  <https://www.ncbi.nlm.nih.gov/nuccore/JX977845.1> |
| *Polytomella capuana* | NC_010357.1 | <https://www.ncbi.nlm.nih.gov/nuccore/NC_010357.1> |
| *Polytomella capuana* strain SAG 63-5 | EF645804.1 | <https://www.ncbi.nlm.nih.gov/nuccore/EF645804.1> |
| *Polytomella* sp. SAG 63-10 chromosome 1 | NC_013472.1  GU108480.1 | https://www.ncbi.nlm.nih.gov/nuccore/NC_013472.1  <https://www.ncbi.nlm.nih.gov/nuccore/GU108480.1> |
| *Polytomella parva* mitochondrion chromosome 1 | NC_016916.1 | <https://www.ncbi.nlm.nih.gov/nuccore/NC_016916.1> |
| *Polytomella parva* strain UTEX L 193 chromosome 1 | AY062933.1 | <https://www.ncbi.nlm.nih.gov/nuccore/AY062933.1> |
| *Polytomella parva strain* UTEX L 193 chromosome 2 | AY062934.1 | <https://www.ncbi.nlm.nih.gov/nuccore/AY062934.1> |
| *Polytomella magna* | KC733827.1 | <https://www.ncbi.nlm.nih.gov/nuccore/KC733827.1> |
| *Polytoma uvella* strain UTEX 964 | NC_026572.1  KP696388.1 | https://www.ncbi.nlm.nih.gov/nuccore/NC_026572.1  <https://www.ncbi.nlm.nih.gov/nuccore/KP696388.1> |
| *Dunaliella salina* | NC_012930.1 | <https://www.ncbi.nlm.nih.gov/nuccore/NC_012930.1> |
| *Dunaliella salina* strain CCAP 19/18 | GQ250045.1 | <https://www.ncbi.nlm.nih.gov/nuccore/GQ250045.1> |
| *Dunaliella salina* strain SQ | KX641170.1 | <https://www.ncbi.nlm.nih.gov/nuccore/KX641170.1> |
| *Dunaliella salina* strain GN | KX641169.1 | <https://www.ncbi.nlm.nih.gov/nuccore/KX641169.1> |
| *Dunaliella viridis* strain CCM-UDEC 002 | NC_026571.1  KP691602.1 | https://www.ncbi.nlm.nih.gov/nuccore/NC_026571.1  <https://www.ncbi.nlm.nih.gov/nuccore/KP691602.1> |
| *Chlamydomonas reinhardtii* | NC_001638.1  U03843.1 | https://www.ncbi.nlm.nih.gov/nuccore/NC_001638.1  https://www.ncbi.nlm.nih.gov/nuccore/U03843.1 |
| *Chlamydomonas reinhardtii* strain CC-1952 mt- | EU306621.1 | <https://www.ncbi.nlm.nih.gov/nuccore/EU306621.1> |
| *Chlamydomonas reinhardtii* strain CC-2342 mt- | EU306620.1 | <https://www.ncbi.nlm.nih.gov/nuccore/EU306620.1> |
| *Chlamydomonas reinhardtii* strain CC-2343 mt+ | EU306623.1 | <https://www.ncbi.nlm.nih.gov/nuccore/EU306623.1> |
| *Chlamydomonas reinhardtii* strain CC-2344 mt+ | EU306619.1 | <https://www.ncbi.nlm.nih.gov/nuccore/EU306619.1> |
| *Chlamydomonas reinhardtii* strain CC-277 cw15 mt+ | EU306622.1 | <https://www.ncbi.nlm.nih.gov/nuccore/EU306622.1> |
| *Chlamydomonas reinhardtii* strain CC-2931 mt- | EU306618.1 | <https://www.ncbi.nlm.nih.gov/nuccore/EU306618.1> |
| *Chlamydomonas reinhardtii* strain CC-1373 mt+ | EU306617.1 | <https://www.ncbi.nlm.nih.gov/nuccore/EU306617.1> |
| *Chlamydomonas moewusii* | NC_001872.1 | <https://www.ncbi.nlm.nih.gov/nuccore/NC_001872.1> |
| *Chlamydomonas moewusii* strain UTEX 9 | AF008237.1 | <https://www.ncbi.nlm.nih.gov/nuccore/AF008237.1> |
| *Chlamydomonas leiostraca* strain SAG 11-49 | NC_026573.1  KP696389.1 | https://www.ncbi.nlm.nih.gov/nuccore/NC_026573.1  <https://www.ncbi.nlm.nih.gov/nuccore/KP696389.1> |
| *Chlamydomonas* sp. UWO241 | MH598508.1 | <https://www.ncbi.nlm.nih.gov/nuccore/MH598508.1> |
| *Chlorosarcinopsis* *eremi* strain MKA.28 | NC_041430.1  MH665695.1 | https://www.ncbi.nlm.nih.gov/nuccore/NC_041430.1  <https://www.ncbi.nlm.nih.gov/nuccore/MH665695.1> |
| *Pseudomuriella* *schumacherensis* strain SAG 2137 | NC_024763.1  KJ806273.1 | https://www.ncbi.nlm.nih.gov/nuccore/NC_024763.1  <https://www.ncbi.nlm.nih.gov/nuccore/KJ806273.1> |
| *Pediastrum* *duplex* Group II strain PL0501b | KR026340.1 | <https://www.ncbi.nlm.nih.gov/nuccore/KR026340.1> |
| *Pediastrum duplex* Group I strain AL0403MN | KR026339.1 | <https://www.ncbi.nlm.nih.gov/nuccore/KR026339.1> |
| *Chloroparvula pacifica* culture RCC:4656 | NC_042603.1  MK086011.1 | https://www.ncbi.nlm.nih.gov/nuccore/NC_042603.1  <https://www.ncbi.nlm.nih.gov/nuccore/MK086011.1> |
| *Chloroparvula* *japonica* culture RCC:2339 | NC_042601.1  MK086007.1 | https://www.ncbi.nlm.nih.gov/nuccore/NC_042601.1  <https://www.ncbi.nlm.nih.gov/nuccore/MK086007.1> |
| *Chloroparvula* sp. RCC4572 culture RCC:4572 | MK086010.1 | <https://www.ncbi.nlm.nih.gov/nuccore/MK086010.1> |
| *Chloroparvula* sp. RCC999 culture RCC:999 | MK086002.1 | <https://www.ncbi.nlm.nih.gov/nuccore/MK086002.1> |
| *Chloroparvula* sp. RCC696 culture RCC:696 | MK085999.1 | <https://www.ncbi.nlm.nih.gov/nuccore/MK085999.1> |
| *Chloropicon* *maureeniae* culture RCC:3374 | NC_042602.1  MK086008.1 | https://www.ncbi.nlm.nih.gov/nuccore/NC_042602.1  <https://www.ncbi.nlm.nih.gov/nuccore/MK086008.1> |
| *Chloropicon* *mariensis* culture RCC:998 | NC_042600.1  MK086005.1 | https://www.ncbi.nlm.nih.gov/nuccore/NC_042600.1  <https://www.ncbi.nlm.nih.gov/nuccore/MK086005.1> |
| *Chloropicon* *roscoffensis* culture RCC:1871 | NC_042599.1  MK086004.1 | https://www.ncbi.nlm.nih.gov/nuccore/NC_042599.1  <https://www.ncbi.nlm.nih.gov/nuccore/MK086004.1> |
| *Chloropicon roscoffensis* culture RCC:2335 | MK086006.1 | <https://www.ncbi.nlm.nih.gov/nuccore/MK086006.1> |
| *Chloropicon sieburthii* culture RCC:287 | NC_042598.1  MK086003.1 | https://www.ncbi.nlm.nih.gov/nuccore/NC_042598.1  <https://www.ncbi.nlm.nih.gov/nuccore/MK086003.1> |
| *Chloropicon laureae* culture RCC:856 | NC_042492.1  MK086001.1 | https://www.ncbi.nlm.nih.gov/nuccore/NC_042492.1  <https://www.ncbi.nlm.nih.gov/nuccore/MK086001.1> |
| *Chloropicon* sp. RCC4434 culture RCC:4434 | MK086009.1 | <https://www.ncbi.nlm.nih.gov/nuccore/MK086009.1> |
| *Chloropicon* *primus* culture CCMP:1205 | NC_042490.1  MK085998.1 | https://www.ncbi.nlm.nih.gov/nuccore/NC_042490.1  <https://www.ncbi.nlm.nih.gov/nuccore/MK085998.1> |
| *Ostreococcus tauri* | NC_008290.1  CR954200.2 | https://www.ncbi.nlm.nih.gov/nuccore/NC_008290.1  <https://www.ncbi.nlm.nih.gov/nuccore/CR954200.2> |
| *Ostreococcus tauri* isolate RCC745-2009 | KC967294.1 | <https://www.ncbi.nlm.nih.gov/nuccore/KC967294.1> |
| *Ostreococcus tauri* isolate RCC1108 | KC967295.1 | <https://www.ncbi.nlm.nih.gov/nuccore/KC967295.1> |
| *Ostreococcus tauri* isolate RCC1110 | KC967296.1 | <https://www.ncbi.nlm.nih.gov/nuccore/KC967296.1> |
| *Ostreococcus tauri* isolate RCC1112 | KC967297.1 | <https://www.ncbi.nlm.nih.gov/nuccore/KC967297.1> |
| *Ostreococcus tauri* isolate RCC1114 | KC967298.1 | <https://www.ncbi.nlm.nih.gov/nuccore/KC967298.1> |
| *Ostreococcus tauri* isolate RCC1115 | KC967299.1 | <https://www.ncbi.nlm.nih.gov/nuccore/KC967299.1> |
| *Ostreococcus tauri* isolate RCC1116 | KC967300.1 | <https://www.ncbi.nlm.nih.gov/nuccore/KC967300.1> |
| *Ostreococcus tauri* isolate RCC1117 | KC967301.1 | <https://www.ncbi.nlm.nih.gov/nuccore/KC967301.1> |
| *Ostreococcus tauri* isolate RCC1118 | KC967302.1 | <https://www.ncbi.nlm.nih.gov/nuccore/KC967302.1> |
| *Ostreococcus tauri* isolate RCC1123 | KC967303.1 | <https://www.ncbi.nlm.nih.gov/nuccore/KC967303.1> |
| *Ostreococcus tauri* isolate RCC1558 | KC967304.1 | <https://www.ncbi.nlm.nih.gov/nuccore/KC967304.1> |
| *Ostreococcus tauri* isolate RCC1559 | KC967305.1 | <https://www.ncbi.nlm.nih.gov/nuccore/KC967305.1> |
| *Ostreococcus tauri* isolate RCC1561 | KC967306.1 | <https://www.ncbi.nlm.nih.gov/nuccore/KC967306.1> |
| *Bathycoccus prasinos* | NC_023273.1 | <https://www.ncbi.nlm.nih.gov/nuccore/NC_023273.1> |
| *Micromonas* sp. RCC299 | NC_012643.1  FJ859351.1 | https://www.ncbi.nlm.nih.gov/nuccore/NC_012643.1  https://www.ncbi.nlm.nih.gov/nuccore/FJ859351.1 |
| *Monomastix* sp.OKE-1 | NC_022797.1  KF060939.1 | https://www.ncbi.nlm.nih.gov/nuccore/NC_022797.1  <https://www.ncbi.nlm.nih.gov/nuccore/KF060939.1> |
| *Nephroselmis* *olivacea* | AF110138.1  NC_008239.1 | https://www.ncbi.nlm.nih.gov/nuccore/AF110138.1  <https://www.ncbi.nlm.nih.gov/nuccore/NC_008239.1> |
| *Pedinomonas* *minor* | NC_000892.1  AF116775.1 | https://www.ncbi.nlm.nih.gov/nuccore/NC_000892.1  <https://www.ncbi.nlm.nih.gov/nuccore/AF116775.1> |
| *Picocystis* *salinarum* culture CCMP:1897 | NC_042491.1  MK086000.1 | https://www.ncbi.nlm.nih.gov/nuccore/NC_042491.1  <https://www.ncbi.nlm.nih.gov/nuccore/MK086000.1> |
| *Prasinoderma coloniale* culture-collection CCMP:1220 | NC_023355.1  KF387569.1 | https://www.ncbi.nlm.nih.gov/nuccore/NC_023355.1  <https://www.ncbi.nlm.nih.gov/nuccore/KF387569.1> |
| *Pycnococcus provasolii* | NC_013935.1  GQ497137.1 | https://www.ncbi.nlm.nih.gov/nuccore/NC_013935.1  <https://www.ncbi.nlm.nih.gov/nuccore/GQ497137.1> |
| *Pyramimonas parkeae*strain SCCAP K-0007 | NC_031504.1  KX756655.1 | https://www.ncbi.nlm.nih.gov/nuccore/NC_031504.1  <https://www.ncbi.nlm.nih.gov/nuccore/KX756655.1> |
| *Pyramimonas parkeae* strain NIES254 | KX013547.1 | <https://www.ncbi.nlm.nih.gov/nuccore/KX013547.1> |
| *Cymbomonas tetramitiformis* strain PLY262 | KX013548.1  NC_036614.1 | https://www.ncbi.nlm.nih.gov/nuccore/KX013548.1  <https://www.ncbi.nlm.nih.gov/nuccore/NC_036614.1> |
| *Botryococcus braunii* | NC_027722.1  KR057902.1 | https://www.ncbi.nlm.nih.gov/nuccore/NC_027722.1  <https://www.ncbi.nlm.nih.gov/nuccore/KR057902.1> |
| *Botryococcus braunii* Showa | LT545992.1 | <https://www.ncbi.nlm.nih.gov/nuccore/LT545992.1> |
| *Lobosphaera incisa* strain SAG 2468 | NC_027060.1  KP902678.1 | https://www.ncbi.nlm.nih.gov/nuccore/NC_027060.1  <https://www.ncbi.nlm.nih.gov/nuccore/KP902678.1> |
| *Trebouxiophyceae* sp. MX-AZ01 | NC_018568.1  JX315601.1 | https://www.ncbi.nlm.nih.gov/nuccore/NC_018568.1  <https://www.ncbi.nlm.nih.gov/nuccore/JX315601.1> |
| *Ourococcus* *multisporus* strain UTEX 1240 | NC_024762.1  KJ806272.1 | https://www.ncbi.nlm.nih.gov/nuccore/NC_024762.1  <https://www.ncbi.nlm.nih.gov/nuccore/KJ806272.1> |
| *Coccomyxa* sp. C-169 | HQ874522.1  NC_015316.1 | https://www.ncbi.nlm.nih.gov/nuccore/HQ874522.1  <https://www.ncbi.nlm.nih.gov/nuccore/NC_015316.1> |
| *Picochlorum* sp. 'soloecismus' strain DOE 101 | MG552670.1 | <https://www.ncbi.nlm.nih.gov/nuccore/MG552670.1> |
| *Prototheca wickerhamii* | NC_001613.1 | <https://www.ncbi.nlm.nih.gov/nuccore/NC_001613.1> |
| *Prototheca zopfii* strain SAG 2021 | MF197534.1 | <https://www.ncbi.nlm.nih.gov/nuccore/MF197534.1> |
| *Prototheca zopfii* strain SAG 2063 | NC_037449.1  MF197533.1 | https://www.ncbi.nlm.nih.gov/nuccore/NC_037449.1  <https://www.ncbi.nlm.nih.gov/nuccore/MF197533.1> |
| *Helicosporidium* sp. ex *Simulium* *jonesi* | GQ339576.1 | <https://www.ncbi.nlm.nih.gov/nuccore/GQ339576.1> |
| *Auxenochlorella* *protothecoides* | NC_026009.1  KC843974.1 | https://www.ncbi.nlm.nih.gov/nuccore/NC_026009.1  <https://www.ncbi.nlm.nih.gov/nuccore/KC843974.1> |
| *Auxenochlorella* *protothecoides* strain UTEX 2341 | KY681419.1 | <https://www.ncbi.nlm.nih.gov/nuccore/KY681419.1> |
| *Micractinium* *conductrix* | KY629619.1 | <https://www.ncbi.nlm.nih.gov/nuccore/KY629619.1> |
| *Micractinium* sp. LBA 32 | MH718999.1 | <https://www.ncbi.nlm.nih.gov/nuccore/MH718999.1> |
| *Chlorella* *heliozoae* | KY629615.1 | <https://www.ncbi.nlm.nih.gov/nuccore/KY629615.1> |
| *Chlorella* *variabilis* isolate NC64A | NC_025413.1  KM252919.1 | https://www.ncbi.nlm.nih.gov/nuccore/NC_025413.1  <https://www.ncbi.nlm.nih.gov/nuccore/KM252919.1> |
| *Chlorella* *variabilis* isolate cvariabilis2014 | KP271968.1 | <https://www.ncbi.nlm.nih.gov/nuccore/KP271968.1> |
| *Chlorella* *sorokiniana* | KM241869.1 | <https://www.ncbi.nlm.nih.gov/nuccore/KM241869.1> |
| *Chlorella* *sorokiniana* isolate 1230 | NC_024626.1  KJ742377.1 | https://www.ncbi.nlm.nih.gov/nuccore/NC_024626.1  <https://www.ncbi.nlm.nih.gov/nuccore/KJ742377.1> |
| *Chlorella* sp. ArM0029B | KF554428.1 | <https://www.ncbi.nlm.nih.gov/nuccore/KF554428.1> |
| *Chlorella* sp. ATCC 30562 | KY629618.1 | <https://www.ncbi.nlm.nih.gov/nuccore/KY629618.1> |
| *Ulva* sp. UNA00071828 | KP720617.1 | <https://www.ncbi.nlm.nih.gov/nuccore/KP720617.1> |
| *Ulva* *prolifera* | NC_028538.1  KT428794.1 | https://www.ncbi.nlm.nih.gov/nuccore/NC_028538.1  <https://www.ncbi.nlm.nih.gov/nuccore/KT428794.1> |
| *Ulva* *prolifera* | KU161104.1 | <https://www.ncbi.nlm.nih.gov/nuccore/KU161104.1> |
| *Ulva* *fasciata* | NC_028081.1  KT364296.1 | https://www.ncbi.nlm.nih.gov/nuccore/NC_028081.1  <https://www.ncbi.nlm.nih.gov/nuccore/KT364296.1> |
| *Ulva* *fasciata* | KU182748.1 | <https://www.ncbi.nlm.nih.gov/nuccore/KU182748.1> |
| *Ulva linza* | NC_029701.1  KU189740.1 | https://www.ncbi.nlm.nih.gov/nuccore/NC_029701.1  <https://www.ncbi.nlm.nih.gov/nuccore/KU189740.1> |
| *Ulva pertusa* | NC_035722.1  KX530816.1 | https://www.ncbi.nlm.nih.gov/nuccore/NC_035722.1  <https://www.ncbi.nlm.nih.gov/nuccore/KX530816.1> |
| *Ulva pertusa* | KX530817.1 | <https://www.ncbi.nlm.nih.gov/nuccore/KX530817.1> |
| *Ulva compressa* | NC_041082.1  KY626327.1 | https://www.ncbi.nlm.nih.gov/nuccore/NC_041082.1  <https://www.ncbi.nlm.nih.gov/nuccore/KY626327.1> |
| *Ulva compressa* | KX595276.1 | <https://www.ncbi.nlm.nih.gov/nuccore/KX595276.1> |
| *Ulva flexuosa* | NC_035809.1  KX455878.1 | https://www.ncbi.nlm.nih.gov/nuccore/NC_035809.1  <https://www.ncbi.nlm.nih.gov/nuccore/KX455878.1> |
| *Ulva flexuosa* | KY626326.1 | <https://www.ncbi.nlm.nih.gov/nuccore/KY626326.1> |
| *Ulva expansa* voucher UC2050480 | NC_040163.1  MH730971.1 | https://www.ncbi.nlm.nih.gov/nuccore/NC_040163.1  <https://www.ncbi.nlm.nih.gov/nuccore/MH730971.1> |
| *Ulva ohnoi* | AP018695.1 | <https://www.ncbi.nlm.nih.gov/nuccore/AP018695.1> |
| *Pseudendoclonium akinetum* | NC_005926.1  AY359242.1 | https://www.ncbi.nlm.nih.gov/nuccore/NC_005926.1  <https://www.ncbi.nlm.nih.gov/nuccore/AY359242.1> |
| *Gloeotilopsis sarcinoidea* culture-collection UTEX:1710 | KX306822.1 | <https://www.ncbi.nlm.nih.gov/nuccore/KX306822.1> |
| *Gloeotilopsis planctonica* culture-collection SAG:29.93 | KX306823.1 | <https://www.ncbi.nlm.nih.gov/nuccore/KX306823.1> |
| *Caulerpa lentillifera* | NC_038217.1  KX761577.1 | https://www.ncbi.nlm.nih.gov/nuccore/NC_038217.1  <https://www.ncbi.nlm.nih.gov/nuccore/KX761577.1> |
| *Oltmannsiellopsis viridis* | NC_008256.1  DQ365900.1 | https://www.ncbi.nlm.nih.gov/nuccore/NC_008256.1  <https://www.ncbi.nlm.nih.gov/nuccore/DQ365900.1> |
| *Jaagichlorella roystonensis* | NC_046060.1  MN934958.1 | https://www.ncbi.nlm.nih.gov/nuccore/NC_046060.1  <https://www.ncbi.nlm.nih.gov/nuccore/MN934958.1> |
| *Bryopsis plumosa* | MN853874.1 | <https://www.ncbi.nlm.nih.gov/nuccore/MN853874.1> |
| *Ulva compressa* | MH093740.1 | <https://www.ncbi.nlm.nih.gov/nuccore/MH093740.1> |
| *Chloroidium* sp. UTEX 3077 | MN646686.1 | <https://www.ncbi.nlm.nih.gov/nuccore/MN646686.1> |
| *Micractinium pusillum* strain CCAP 232/1 | MN649871.1 | <https://www.ncbi.nlm.nih.gov/nuccore/MN649871.1> |
| *Chlorella vulgaris* strain NJ-7 | NC_045362.1  MK948101.1 | https://www.ncbi.nlm.nih.gov/nuccore/NC_045362.1  <https://www.ncbi.nlm.nih.gov/nuccore/MK948101.1> |
| *Chlorella vulgaris* strain UTEX259 | MK948103.1 | <https://www.ncbi.nlm.nih.gov/nuccore/MK948103.1> |
| *Scherffelia dubia* culture SAG:17.86 | NC_045363.1  MN642088.1 | https://www.ncbi.nlm.nih.gov/nuccore/NC_045363.1  <https://www.ncbi.nlm.nih.gov/nuccore/MN642088.1> |
| *Tetraselmis* sp. CCMP 881 | MN642087.1 | <https://www.ncbi.nlm.nih.gov/nuccore/MN642087.1> |
| *Prasinococcus* sp. CCMP1194 | MN662312.1 | <https://www.ncbi.nlm.nih.gov/nuccore/MN662312.1> |
| *Prasinophyte* sp. MBIC10622 | MN662311.1 | <https://www.ncbi.nlm.nih.gov/nuccore/MN662311.1> |
| *Prototheca* *zopfii* strain SAG 2021 | NC_045058.1 | <https://www.ncbi.nlm.nih.gov/nuccore/NC_045058.1> |
| *Caulerpa ashmeadii* voucher TS1851 | NC_045849.1  MH745227.1 | https://www.ncbi.nlm.nih.gov/nuccore/NC_045849.1  <https://www.ncbi.nlm.nih.gov/nuccore/MH745227.1> |
| *Ostreobium quekettii* strain SAG6.99 | NC_045361.1  MN514984.1 | https://www.ncbi.nlm.nih.gov/nuccore/NC_045361.1  <https://www.ncbi.nlm.nih.gov/nuccore/MN514984.1> |
| *Pediastrum duplex* | MK895949.1 | <https://www.ncbi.nlm.nih.gov/nuccore/MK895949.1> |
| *Trebouxia* sp. TR9 | MH917293.1 | <https://www.ncbi.nlm.nih.gov/nuccore/MH917293.1> |
| *Haematococcus* *lacustris* strain UTEX 2505 | NC_044670.1 | <https://www.ncbi.nlm.nih.gov/nuccore/NC_044670.1> |
| *Rhodomonas salina* | NC_002572.1 | <https://www.ncbi.nlm.nih.gov/nuccore/NC_002572.1> |
| *Hemiselmis andersenii* strain CCMP 644 | EU651892.1 | <https://www.ncbi.nlm.nih.gov/nuccore/EU651892.1> |
| *Chroomonas placoidea* strain CCAP978 / 8 | NC_037451.1  MG680941.1 | https://www.ncbi.nlm.nih.gov/nuccore/NC_037451.1  <https://www.ncbi.nlm.nih.gov/nuccore/MG680941.1> |
| *Proteomonas sulcata* strain CCMP705 | NC_037453.1  MG680945.1 | https://www.ncbi.nlm.nih.gov/nuccore/NC_037453.1  <https://www.ncbi.nlm.nih.gov/nuccore/MG680945.1> |
| *Teleaulax amphioxeia* strain HACCP-CR01 | NC_037436.1  MG680944.1 | https://www.ncbi.nlm.nih.gov/nuccore/NC_037436.1  <https://www.ncbi.nlm.nih.gov/nuccore/MG680944.1> |
| *Cryptomonas curvata* strain CNUKR | NC_037454.1  MG680942.1 | https://www.ncbi.nlm.nih.gov/nuccore/NC_037454.1  <https://www.ncbi.nlm.nih.gov/nuccore/MG680942.1> |
| *Hemiselmis andersenii* | NC_010637.1 | <https://www.ncbi.nlm.nih.gov/nuccore/NC_010637.1> |
| *Goniomonas avonlea* | AP018919.1 | <https://www.ncbi.nlm.nih.gov/nuccore/AP018919.1> |
| *Rhodomonas salina* | AF288090.1 | <https://www.ncbi.nlm.nih.gov/nuccore/AF288090.1> |
| *Storeatula* sp. CCMP1868 | NC_037452.1  MG680943.1 | https://www.ncbi.nlm.nih.gov/nuccore/NC_037452.1  <https://www.ncbi.nlm.nih.gov/nuccore/MG680943.1> |
| *Guillardia* *theta* | NC_041490.1  MH844545.1 | https://www.ncbi.nlm.nih.gov/nuccore/NC_041490.1  <https://www.ncbi.nlm.nih.gov/nuccore/MH844545.1> |
| *Cryptophyta* sp. CCMP2293 | MK292549.1 | <https://www.ncbi.nlm.nih.gov/nuccore/MK292549.1> |
| *Leucocryptos marina* NIES-1335 | NC_045933.1  LC515368.1 | https://www.ncbi.nlm.nih.gov/nuccore/NC_045933.1  <https://www.ncbi.nlm.nih.gov/nuccore/LC515368.1> |
| *Hemiarma marina* SRT149 | LC515367.1 | <https://www.ncbi.nlm.nih.gov/nuccore/LC515367.1> |
| *Cyanoptyche gloeocystis* | NC_025294.1 | <https://www.ncbi.nlm.nih.gov/nuccore/NC_025294.1> |
| *Cyanoptyche gloeocystis* strain SAG 4.97 | KJ867411.1 | <https://www.ncbi.nlm.nih.gov/nuccore/KJ867411.1> |
| *Gloeochaete wittrockiana* | NC_025293.1 | <https://www.ncbi.nlm.nih.gov/nuccore/NC_025293.1> |
| *Gloeochaete wittrockiana* strain SAG 46.84 | KJ867410.1 | <https://www.ncbi.nlm.nih.gov/nuccore/KJ867410.1> |
| *Cyanophora paradoxa* | NC_017836.1 | <https://www.ncbi.nlm.nih.gov/nuccore/NC_017836.1> |
| *Cyanophora paradoxa* strain CCMP 329 | HQ849544.1 | <https://www.ncbi.nlm.nih.gov/nuccore/HQ849544.1> |
| *Glaucocystis nostochinearum* | NC_015117.1 | <https://www.ncbi.nlm.nih.gov/nuccore/NC_015117.1> |
| *Glaucocystis nostochinearum* strain UTEX 64 | HQ908425.1 | <https://www.ncbi.nlm.nih.gov/nuccore/HQ908425.1> |
| *Emiliania huxleyi* culture-collection CCMP:1516 | JN022704.1 | <https://www.ncbi.nlm.nih.gov/nuccore/JN022704.1> |
| *Chrysochromulina* sp. CCMP291 | KJ201908.1 | <https://www.ncbi.nlm.nih.gov/nuccore/KJ201908.1> |
| *Emiliania* *huxleyi* | NC_005332.1  AY342361.1 | https://www.ncbi.nlm.nih.gov/nuccore/NC_005332.1  <https://www.ncbi.nlm.nih.gov/nuccore/AY342361.1> |
| *Chrysochromulina* sp. NIES-1333 mitochondrial DNA | AB930144.1 | <https://www.ncbi.nlm.nih.gov/nuccore/AB930144.1> |
| *Chrysochromulina parva* | NC_036938.1  MG520332.1 | https://www.ncbi.nlm.nih.gov/nuccore/NC_036938.1  <https://www.ncbi.nlm.nih.gov/nuccore/MG520332.1> |
| *Pavlova* sp. NIVA-4 / 92 | MN564259.1 | <https://www.ncbi.nlm.nih.gov/nuccore/MN564259.1> |
| *Dictyopteris divaricata* | NC_043845.1  MG940856.1 | https://www.ncbi.nlm.nih.gov/nuccore/NC_043845.1  <https://www.ncbi.nlm.nih.gov/nuccore/MG940856.1> |
| *Chrysodidymus synuroideus* | NC_002174.1  AF222718.1 | https://www.ncbi.nlm.nih.gov/nuccore/NC_002174.1  <https://www.ncbi.nlm.nih.gov/nuccore/AF222718.1> |
| *Laminaria digitata* | NC_004024.1  AJ344328.1 | https://www.ncbi.nlm.nih.gov/nuccore/NC_004024.1  <https://www.ncbi.nlm.nih.gov/nuccore/AJ344328.1> |
| *Ectocarpus siliculosus* | NC_030223.1 | <https://www.ncbi.nlm.nih.gov/nuccore/NC_030223.1> |
| *Pylaiella littoralis* | NC_003055.1 | <https://www.ncbi.nlm.nih.gov/nuccore/NC_003055.1> |
| *Saccharina japonica x latissima* ‘Rongfu’ | NC_015669.1  JF937591.1 | https://www.ncbi.nlm.nih.gov/nuccore/NC_015669.1  https://www.ncbi.nlm.nih.gov/nuccore/JF937591.1 |
| *Fucus distichus* voucher UC2050487 | NC_034672.1  KY678904.1 | https://www.ncbi.nlm.nih.gov/nuccore/NC_034672.1  <https://www.ncbi.nlm.nih.gov/nuccore/KY678904.1> |
| *Saccharina japonica x latissima* cultivar Xinbenniu | MG712780.1 | <https://www.ncbi.nlm.nih.gov/nuccore/MG712780.1> |
| *Saccharina japonica* cultivar Dongfang No.6 | MG712776.1 | <https://www.ncbi.nlm.nih.gov/nuccore/MG712776.1> |
| *Saccharina japonica* isolate Zaohoucheng | KX073816.1 | <https://www.ncbi.nlm.nih.gov/nuccore/KX073816.1> |
| *Saccharina japonica x latissima* Pingbancai | KX073817.1 | <https://www.ncbi.nlm.nih.gov/nuccore/KX073817.1> |
| *Saccharina japonica* | NC_013476.1 | <https://www.ncbi.nlm.nih.gov/nuccore/NC_013476.1> |
| *Saccharina japonica* isolate Fujian | KX073815.1 | <https://www.ncbi.nlm.nih.gov/nuccore/KX073815.1> |
| *Laminaria hyperborea* | NC_021639.1  JN099683.1 | https://www.ncbi.nlm.nih.gov/nuccore/NC_021639.1  JN099683.1 |
| *Saccharina longipedalis* | NC_013484.1 | <https://www.ncbi.nlm.nih.gov/nuccore/NC_013484.1> |
| *Saccharina longissima* | NC_021640.1  JN099684.1 | https://www.ncbi.nlm.nih.gov/nuccore/NC_021640.1  <https://www.ncbi.nlm.nih.gov/nuccore/JN099684.1> |
| *Macrocystis integrifolia* | NC_042669.1  MH411105.1 | https://www.ncbi.nlm.nih.gov/nuccore/NC_042669.1  <https://www.ncbi.nlm.nih.gov/nuccore/MH411105.1> |
| *Vischeria stellata* strain SAG 33.83 | NC_041506.1  MH981596.1 | https://www.ncbi.nlm.nih.gov/nuccore/NC_041506.1  <https://www.ncbi.nlm.nih.gov/nuccore/MH981596.1> |
| *Saccharina latissima* strain ye-C14 | NC_026108.1  KM675818.1 | https://www.ncbi.nlm.nih.gov/nuccore/NC_026108.1  <https://www.ncbi.nlm.nih.gov/nuccore/KM675818.1> |
| *Saccharina* sp. ye-W | KR056087.1 | <https://www.ncbi.nlm.nih.gov/nuccore/KR056087.1> |
| *Saccharina* sp. ye-G | KM657964.1 | <https://www.ncbi.nlm.nih.gov/nuccore/KM657964.1> |
| *Saccharina* sp. ye-F | KP058499.1 | <https://www.ncbi.nlm.nih.gov/nuccore/KP058499.1> |
| *Saccharina* sp. ye-C5 | KT336421.1 | <https://www.ncbi.nlm.nih.gov/nuccore/KT336421.1> |
| *Saccharina* sp. ye-C2-2 | KT336420.1 | <https://www.ncbi.nlm.nih.gov/nuccore/KT336420.1> |
| *Saccharina* sp. ye-C | KT315643.1 | <https://www.ncbi.nlm.nih.gov/nuccore/KT315643.1> |
| *Saccharina* sp. ye-C6 | KT271760.1 | <https://www.ncbi.nlm.nih.gov/nuccore/KT271760.1> |
| *Saccharina* sp. ye-C12 | NC_028211.1  KT428594.1 | https://www.ncbi.nlm.nih.gov/nuccore/NC_028211.1  <https://www.ncbi.nlm.nih.gov/nuccore/KT428594.1> |
| *Coccophora* *langsdorfii* | NC_032287.1  KU255794.1 | https://www.ncbi.nlm.nih.gov/nuccore/NC_032287.1  <https://www.ncbi.nlm.nih.gov/nuccore/KU255794.1> |
| *Sargassum* *confusum* | MG459430.1 | <https://www.ncbi.nlm.nih.gov/nuccore/MG459430.1> |
| *Nannochloropsis* *limnetica* strain CCMP505 | NC_022256.1  KC568459.1 | https://www.ncbi.nlm.nih.gov/nuccore/NC_022256.1  <https://www.ncbi.nlm.nih.gov/nuccore/KC568459.1> |
| *Nannochloropsis* *oculata* strain CCMP525 | NC_022257.1  KC568460.1 | https://www.ncbi.nlm.nih.gov/nuccore/NC_022257.1  <https://www.ncbi.nlm.nih.gov/nuccore/KC568460.1> |
| *Nannochloropsis* *gaditana* strain CCMP527 | KC568461.1 | <https://www.ncbi.nlm.nih.gov/nuccore/KC568461.1> |
| *Nannochloropsis* *granulata* strain CCMP529 | NC_022254.1  KC568457.1 | https://www.ncbi.nlm.nih.gov/nuccore/NC_022254.1  <https://www.ncbi.nlm.nih.gov/nuccore/KC568457.1> |
| *Nannochloropsis* *oceanica* strain CCMP531 | KC568462.1 | <https://www.ncbi.nlm.nih.gov/nuccore/KC568462.1> |
| *Nannochloropsis* *oceanica* strain IMET1 | KC568456.1 | <https://www.ncbi.nlm.nih.gov/nuccore/KC568456.1> |
| *Nannochloropsis* *salina* strain CCMP537 | NC_022255.1  KC568458.1 | https://www.ncbi.nlm.nih.gov/nuccore/NC_022255.1  <https://www.ncbi.nlm.nih.gov/nuccore/KC568458.1> |
| *Heterosigma* *akashiwo* strain HaTj01 | KU561550.1 | <https://www.ncbi.nlm.nih.gov/nuccore/KU561550.1> |
| *Heterosigma* *akashiwo* strain Ha00_17 | KU561548.1 | <https://www.ncbi.nlm.nih.gov/nuccore/KU561548.1> |
| *Heterosigma* *akashiwo* strain H93616 | KU561547.1 | <https://www.ncbi.nlm.nih.gov/nuccore/KU561547.1> |
| *Heterosigma* *akashiwo* isolate NEPCC522 | KU726247.1 | <https://www.ncbi.nlm.nih.gov/nuccore/KU726247.1> |
| *Turbinaria ornata* | NC_027413.1  KM501562.1 | https://www.ncbi.nlm.nih.gov/nuccore/NC_027413.1  <https://www.ncbi.nlm.nih.gov/nuccore/KM501562.1> |
| *Sargassum thunbergii* | NC_026700.1  KP280065.1 | https://www.ncbi.nlm.nih.gov/nuccore/NC_026700.1  <https://www.ncbi.nlm.nih.gov/nuccore/KP280065.1> |
| *Saccharina angustata* | NC_013473.1 | <https://www.ncbi.nlm.nih.gov/nuccore/NC_013473.1> |
| *Saccharina coriacea* | NC_013475.1 | <https://www.ncbi.nlm.nih.gov/nuccore/NC_013475.1> |
| *Endarachne binghamiae* | NC_036747.1  MF374731.1 | https://www.ncbi.nlm.nih.gov/nuccore/NC_036747.1  <https://www.ncbi.nlm.nih.gov/nuccore/MF374731.1> |
| *Pleurocladia lacustris* strain Sa2 | NC_032046.1  KU164873.1 | https://www.ncbi.nlm.nih.gov/nuccore/NC_032046.1  <https://www.ncbi.nlm.nih.gov/nuccore/KU164873.1> |
| *Pleurocladia lacustris* strain SAG 25.93 | KU164874.1 | <https://www.ncbi.nlm.nih.gov/nuccore/KU164874.1> |
| *Saccharina japonica x latissima* cultivar Ailunwan | KU556731.1 | <https://www.ncbi.nlm.nih.gov/nuccore/KU556731.1> |
| *Fucus vesiculosus* | NC_007683.1  AY494079.1 | https://www.ncbi.nlm.nih.gov/nuccore/NC_007683.1  <https://www.ncbi.nlm.nih.gov/nuccore/AY494079.1> |
| *Dictyota dichotoma* | NC_007685.1  AY500368.1 | https://www.ncbi.nlm.nih.gov/nuccore/NC_007685.1  <https://www.ncbi.nlm.nih.gov/nuccore/AY500368.1> |
| *Desmarestia viridis* | NC_007684.1  AY500367.1 | https://www.ncbi.nlm.nih.gov/nuccore/NC_007684.1  <https://www.ncbi.nlm.nih.gov/nuccore/AY500367.1> |
| *Undaria pinnatifida* | NC_023354.1  KF319031.1 | https://www.ncbi.nlm.nih.gov/nuccore/NC_023354.1  <https://www.ncbi.nlm.nih.gov/nuccore/KF319031.1> |
| *Sargassum muticum* | NC_024614.1  KJ938301.1 | https://www.ncbi.nlm.nih.gov/nuccore/NC_024614.1  <https://www.ncbi.nlm.nih.gov/nuccore/KJ938301.1> |
| *Sargassum horneri* | NC_024613.1  KJ938300.1 | https://www.ncbi.nlm.nih.gov/nuccore/NC_024613.1  <https://www.ncbi.nlm.nih.gov/nuccore/KJ938300.1> |
| *Scytosiphon lomentaria* | NC_025240.1  KJ995702.1 | https://www.ncbi.nlm.nih.gov/nuccore/NC_025240.1  <https://www.ncbi.nlm.nih.gov/nuccore/KJ995702.1> |
| *Sargassum vachellianum* | NC_027508.1  KR132242.1 | https://www.ncbi.nlm.nih.gov/nuccore/NC_027508.1  <https://www.ncbi.nlm.nih.gov/nuccore/KR132242.1> |
| *Sargassum hemiphyllum* | NC_024861.1  KM210510.1 | https://www.ncbi.nlm.nih.gov/nuccore/NC_024861.1  <https://www.ncbi.nlm.nih.gov/nuccore/KM210510.1> |
| *Sargassum fusiforme* | NC_024655.1  KJ946428.1 | https://www.ncbi.nlm.nih.gov/nuccore/NC_024655.1  <https://www.ncbi.nlm.nih.gov/nuccore/KJ946428.1> |
| *Colpomenia peregrina* | NC_025302.1  KM244739.1 | https://www.ncbi.nlm.nih.gov/nuccore/NC_025302.1  <https://www.ncbi.nlm.nih.gov/nuccore/KM244739.1> |
| *Sargassum yezoense* | NC_038156.1  MG674825.1 | https://www.ncbi.nlm.nih.gov/nuccore/NC_038156.1  <https://www.ncbi.nlm.nih.gov/nuccore/MG674825.1> |
| *Costaria costata* | NC_023506.1  KF384641.1 | https://www.ncbi.nlm.nih.gov/nuccore/NC_023506.1  <https://www.ncbi.nlm.nih.gov/nuccore/KF384641.1> |
| *Sargassum natans* isolate *Sargassum natans* I | NC_033384.1  KY084907.1 | https://www.ncbi.nlm.nih.gov/nuccore/NC_033384.1  <https://www.ncbi.nlm.nih.gov/nuccore/KY084907.1> |
| *Nereocystis luetkeana* | NC_042395.1  MH392199.1 | https://www.ncbi.nlm.nih.gov/nuccore/NC_042395.1  <https://www.ncbi.nlm.nih.gov/nuccore/MH392199.1> |
| *Nannochloropsis oculata* strain CCMP525 | KJ410688.1 | <https://www.ncbi.nlm.nih.gov/nuccore/KJ410688.1> |
| *Nannochloropsis gaditana* strain CCMP526 | KJ410686.1 | <https://www.ncbi.nlm.nih.gov/nuccore/KJ410686.1> |
| *Nannochloropsis oceanica* strain LAMB0001 | KJ410687.1 | <https://www.ncbi.nlm.nih.gov/nuccore/KJ410687.1> |
| *Nannochloropsis salina* strain CCMP1776 | KJ410689.1 | <https://www.ncbi.nlm.nih.gov/nuccore/KJ410689.1> |
| *Vischeria* sp. CAUP Q 202 | KU501221.1 | <https://www.ncbi.nlm.nih.gov/nuccore/KU501221.1> |
| *Monodopsis* sp. MarTras21 | KU501222.1 | <https://www.ncbi.nlm.nih.gov/nuccore/KU501222.1> |
| *Trachydiscus minutus* strain CCALA 838 | NC_029643.1 | <https://www.ncbi.nlm.nih.gov/nuccore/NC_029643.1> |
| *Sargassum horneri* isolate SS2017-1_R | MH590363.1 | <https://www.ncbi.nlm.nih.gov/nuccore/MH590363.1> |
| *Sargassum horneri* isolate SS2017-2_R | MH590365.1 | <https://www.ncbi.nlm.nih.gov/nuccore/MH590365.1> |
| *Sargassum horneri* isolate SS2017-1_F | MH590364.1 | <https://www.ncbi.nlm.nih.gov/nuccore/MH590364.1> |
| *Sargassum horneri* isolate SS2017-2_F | MH590366.1 | <https://www.ncbi.nlm.nih.gov/nuccore/MH590366.1> |
| *Sargassum horneri* ecotype KR-1 | MH586537.1 | <https://www.ncbi.nlm.nih.gov/nuccore/MH586537.1> |
| *Sargassum horneri* ecotype KR-2 | MH586538.1 | <https://www.ncbi.nlm.nih.gov/nuccore/MH586538.1> |
| *Sargassum horneri* isolate YS2017-3 | MH590362.1 | <https://www.ncbi.nlm.nih.gov/nuccore/MH590362.1> |
| *Sargassum horneri* isolate YS2017-4 | MH590361.1 | <https://www.ncbi.nlm.nih.gov/nuccore/MH590361.1> |
| *Sargassum horneri* isolate YS2017-5 | MH590360.1 | <https://www.ncbi.nlm.nih.gov/nuccore/MH590360.1> |
| *Sargassum horneri* isolate YS2017-6 | MH590359.1 | <https://www.ncbi.nlm.nih.gov/nuccore/MH590359.1> |
| *Sargassum horneri* isolate YS2017-7 | MH590358.1 | <https://www.ncbi.nlm.nih.gov/nuccore/MH590358.1> |
| *Sargassum spinuligerum* | NC_034272.1  KT276514.1 | https://www.ncbi.nlm.nih.gov/nuccore/NC_034272.1  <https://www.ncbi.nlm.nih.gov/nuccore/KT276514.1> |
| *Sargassum ilicifolium* | KT272403.1 | <https://www.ncbi.nlm.nih.gov/nuccore/KT272403.1> |
| *Sargassum polycystum* | KT280278.1 | <https://www.ncbi.nlm.nih.gov/nuccore/KT280278.1> |
| *Sargassum fluitans* clone C256-039-NT_38 | NC_033385.1  KY084909.1 | https://www.ncbi.nlm.nih.gov/nuccore/NC_033385.1  <https://www.ncbi.nlm.nih.gov/nuccore/KY084909.1> |
| *Sargassum fluitans* isolate *Sargassum fluitans* III | KY084910.1 | <https://www.ncbi.nlm.nih.gov/nuccore/KY084910.1> |
| *Sargassum aquifolium* | NC_033408.1  KT266809.1 | https://www.ncbi.nlm.nih.gov/nuccore/NC_033408.1  <https://www.ncbi.nlm.nih.gov/nuccore/KT266809.1> |
| *Sargassum natans* clone C256-039-NT_32 | KY084908.1 | <https://www.ncbi.nlm.nih.gov/nuccore/KY084908.1> |
| *Sargassum natans* clone C263B-St._Croix_Hbr_145 | KY084911.1 | <https://www.ncbi.nlm.nih.gov/nuccore/KY084911.1> |
| *Sargassum natans* isolate *Sargassum natans* VIII | KY084912.1 | <https://www.ncbi.nlm.nih.gov/nuccore/KY084912.1> |
| *Fucus vesiculosus* var. *spiralis* voucher UC 2050586 | MG922856.1 | <https://www.ncbi.nlm.nih.gov/nuccore/MG922856.1> |
| *Saccharina japonica* mitochondrial DNA,isolate: SJAPO | AP011493.1 | <https://www.ncbi.nlm.nih.gov/nuccore/AP011493.1> |
| *Saccharina angustata* mitochondrial DNA, isolate: SANGU | AP011498.1 | <https://www.ncbi.nlm.nih.gov/nuccore/AP011498.1> |
| *Saccharina coriacea* mitochondrial DNA,isolate: SCORI | AP011499.1 | <https://www.ncbi.nlm.nih.gov/nuccore/AP011499.1> |
| *Saccharina longipedalis* mitochondrial DN，isolate: SLOPE | AP011497.1 | <https://www.ncbi.nlm.nih.gov/nuccore/AP011497.1> |
| *Saccharina ochotensis* | NC_013478.1 | <https://www.ncbi.nlm.nih.gov/nuccore/NC_013478.1> |
| *Saccharina ochotensis* mitochondrial DNA,isolate: SOCHO | AP011495.1 | <https://www.ncbi.nlm.nih.gov/nuccore/AP011495.1> |
| *Saccharina diabolica* | NC_013482.1 | <https://www.ncbi.nlm.nih.gov/nuccore/NC_013482.1> |
| *Saccharina diabolica* mitochondrial DNA,isolate: SDIAB | AP011496.1 | <https://www.ncbi.nlm.nih.gov/nuccore/AP011496.1> |
| *Saccharina religiosa* | NC_013477.1 | <https://www.ncbi.nlm.nih.gov/nuccore/NC_013477.1> |
| *Saccharina religiosa* mitochondrial DNA，isolate: SRELI | AP011494.1 | <https://www.ncbi.nlm.nih.gov/nuccore/AP011494.1> |
| *Saccharina sculpera* | NC_029206.1  KR350664.1 | https://www.ncbi.nlm.nih.gov/nuccore/NC_029206.1  <https://www.ncbi.nlm.nih.gov/nuccore/KR350664.1> |
| *Saccharina* sp. ye-B | NC_028192.1  KT356873.1 | https://www.ncbi.nlm.nih.gov/nuccore/NC_028192.1  <https://www.ncbi.nlm.nih.gov/nuccore/KT356873.1> |
| *Saccharina* sp. cultivar Haiyi No. 1 | NC_040854.1  MG712778.1 | https://www.ncbi.nlm.nih.gov/nuccore/NC_040854.1  <https://www.ncbi.nlm.nih.gov/nuccore/MG712778.1> |
| *Saccharina* *japonica* x *longissima* cultivar Dongfang No.3 | NC_040853.1  MG712777.1 | https://www.ncbi.nlm.nih.gov/nuccore/NC_040853.1  <https://www.ncbi.nlm.nih.gov/nuccore/MG712777.1> |
| *Saccharina* *japonica* x *latissima* cultivar Sanhai | MG712779.1 | <https://www.ncbi.nlm.nih.gov/nuccore/MG712779.1> |
| *Saccharina* *japonica* x *latissima* cultivar Shichang | MG712781.1 | <https://www.ncbi.nlm.nih.gov/nuccore/MG712781.1> |
| *Saccharina* *japonica* x *latissima* cultivar Pengza No2 | MF622087.1 | <https://www.ncbi.nlm.nih.gov/nuccore/MF622087.1> |
| *Cladosiphon* *okamuranus* | NC_040224.1  MG488292.1 | https://www.ncbi.nlm.nih.gov/nuccore/NC_040224.1  <https://www.ncbi.nlm.nih.gov/nuccore/MG488292.1> |
| *Petalonia fascia* | NC_025227.1  KJ957769.1 | https://www.ncbi.nlm.nih.gov/nuccore/NC_025227.1  <https://www.ncbi.nlm.nih.gov/nuccore/KJ957769.1> |
| *Endarachne binghamiae* | MG488291.1 | <https://www.ncbi.nlm.nih.gov/nuccore/MG488291.1> |
| *Pilayella littoralis* | AJ277126.1 | <https://www.ncbi.nlm.nih.gov/nuccore/AJ277126.1> |
| *Ochromonas danica* | NC_002571.1 | <https://www.ncbi.nlm.nih.gov/nuccore/NC_002571.1> |
| *Ochromonas danica* mitochondrial DNA | AF287134.1 | <https://www.ncbi.nlm.nih.gov/nuccore/AF287134.1> |
| *Chattonella marina* strain KA11-m-1 | NC_013837.1 | <https://www.ncbi.nlm.nih.gov/nuccore/NC_013837.1> |
| *Heterosigma akashiwo* isolate CCMP452 | GQ222228.1 | <https://www.ncbi.nlm.nih.gov/nuccore/GQ222228.1> |
| *Heterosigma akashiwo* isolate NIES293 | GQ222227.1 | <https://www.ncbi.nlm.nih.gov/nuccore/GQ222227.1> |
| *Heterosigma akashiwo* mitochondrial DNA，strain: Y | NC_016738.1  AB546637.2 | https://www.ncbi.nlm.nih.gov/nuccore/NC_016738.1  <https://www.ncbi.nlm.nih.gov/nuccore/AB546637.2> |
| *Heterosigma akashiwo* mitochondrial DNA，strain: Haek95051 | LC229078.1 | <https://www.ncbi.nlm.nih.gov/nuccore/LC229078.1> |
| *Heterosigma akashiwo* mitochondrial DNA，strain: EHUSP1 | LC229077.1 | https://www.ncbi.nlm.nih.gov/nuccore/LC229077.1 |
| *Heterosigma akashiwo* mitochondrial DNA，strain: CCMP1870 | LC229076.1 | <https://www.ncbi.nlm.nih.gov/nuccore/LC229076.1> |
| *Heterosigma akashiwo* CCAP934-3 mitochondrial DNA | LC384957.1 | <https://www.ncbi.nlm.nih.gov/nuccore/LC384957.1> |
| *Heterosigma akashiwo* mitochondrial DNA，strain: CCAP934-4 | LC228566.1 | <https://www.ncbi.nlm.nih.gov/nuccore/LC228566.1> |
| *Heterosigma akashiwo* CCAP934-7 mitochondrial DNA | LC384958.1 | <https://www.ncbi.nlm.nih.gov/nuccore/LC384958.1> |
| *Heterosigma akashiwo* mitochondrial DNA，strain: CCAP934-8 | LC228564.1 | <https://www.ncbi.nlm.nih.gov/nuccore/LC228564.1> |
| *Heterosigma akashiwo* mitochondrial DNA，strain: CCAP934-9 | LC228565.1 | <https://www.ncbi.nlm.nih.gov/nuccore/LC228565.1> |
| *Heterosigma akashiwo* mitochondrial DNA，strain: Haek9806-1 | LC228563.1 | <https://www.ncbi.nlm.nih.gov/nuccore/LC228563.1> |
| *Heterosigma akashiwo* mitochondrial DNA，strain: HaFk01 | LC228562.1 | <https://www.ncbi.nlm.nih.gov/nuccore/LC228562.1> |
| *Heterosigma akashiwo* mitochondrial DNA，strain: CCMP3374 | LC228561.1 | <https://www.ncbi.nlm.nih.gov/nuccore/LC228561.1> |
| *Heterosigma akashiwo* mitochondrial DNA，strain: CCMP2270 | LC228560.1 | <https://www.ncbi.nlm.nih.gov/nuccore/LC228560.1> |
| *Heterosigma akashiwo* Haek9806-2 mitochondrial DNA | LC384962.1 | <https://www.ncbi.nlm.nih.gov/nuccore/LC384962.1> |
| *Heterosigma akashiwo* RCC1502 mitochondrial DNA | LC384966.1 | <https://www.ncbi.nlm.nih.gov/nuccore/LC384966.1> |
| *Heterosigma akashiwo* CAW05 mitochondrial DNA | LC384965.1 | <https://www.ncbi.nlm.nih.gov/nuccore/LC384965.1> |
| *Heterosigma akashiwo* BrRJ5 mitochondrial DNA | LC384964.1 | <https://www.ncbi.nlm.nih.gov/nuccore/LC384964.1> |
| *Heterosigma akashiwo* BrRJ1 mitochondrial DNA | LC384963.1 | <https://www.ncbi.nlm.nih.gov/nuccore/LC384963.1> |
| *Heterosigma akashiwo* HaGS95 mitochondrial DNA | LC384961.1 | <https://www.ncbi.nlm.nih.gov/nuccore/LC384961.1> |
| *Heterosigma akashiwo* CCMP1595 mitochondrial DNA | LC384959.1 | <https://www.ncbi.nlm.nih.gov/nuccore/LC384959.1> |
| *Heterosigma akashiwo* CCMP1596 mitochondrial DNA | LC384960.1 | <https://www.ncbi.nlm.nih.gov/nuccore/LC384960.1> |
| *Nannochloropsis gaditana* | NC_020015.1  KC012945.1 | https://www.ncbi.nlm.nih.gov/nuccore/NC_020015.1  <https://www.ncbi.nlm.nih.gov/nuccore/KC012945.1> |
| *Eustigmatos* cf. *polyphem* strain CAUP H4302 | MK170182.1 | <https://www.ncbi.nlm.nih.gov/nuccore/MK170182.1> |
| *Nannochloropsis* *oceanica* strain BR2 | CP044615.1 | <https://www.ncbi.nlm.nih.gov/nuccore/CP044615.1> |
| *Nannochloropsis oceanica* strain KB1 | CP044583.1 | <https://www.ncbi.nlm.nih.gov/nuccore/CP044583.1> |
| *Ectocarpus siliculosus* | MK045263.1 | <https://www.ncbi.nlm.nih.gov/nuccore/MK045263.1> |
| *Saccharina* *japonica* x *Saccharina* *latissima* cultivar Rongfu2011 | MK170141.1 | <https://www.ncbi.nlm.nih.gov/nuccore/MK170141.1> |
| *Saccharina* *japonica* x *Saccharina* *latissima* cultivar Rongfu2010 | MK170140.1 | <https://www.ncbi.nlm.nih.gov/nuccore/MK170140.1> |
| *Saccharina* *japonica* x *Saccharina* *latissima* cultivar Rongfu2009 | MK170139.1 | <https://www.ncbi.nlm.nih.gov/nuccore/MK170139.1> |
| *Sargassum* *horneri* isolate YS2017-1 | MG681099.1 | <https://www.ncbi.nlm.nih.gov/nuccore/MG681099.1> |
| *Sargassum* *horneri* isolate YS2017-2 | MG681097.1 | <https://www.ncbi.nlm.nih.gov/nuccore/MG681097.1> |
| *Sargassum* *horneri* isolate YS2016-1 | MG681098.1 | <https://www.ncbi.nlm.nih.gov/nuccore/MG681098.1> |
| *Sargassum* *horneri* isolate YS2016-2 | MG681096.1 | <https://www.ncbi.nlm.nih.gov/nuccore/MG681096.1> |
| *Aureococcus* *anophagefferens* | MK922345.1 | <https://www.ncbi.nlm.nih.gov/nuccore/MK922345.1> |
| *Kappaphycus* *striatus* | NC_024265.1  KF833365.1 | https://www.ncbi.nlm.nih.gov/nuccore/NC_024265.1  <https://www.ncbi.nlm.nih.gov/nuccore/KF833365.1> |
| *Grateloupia taiwanensis* | KM999231.1 | <https://www.ncbi.nlm.nih.gov/nuccore/KM999231.1> |
| *Pyropia endiviifolia* | KU356193.1 | <https://www.ncbi.nlm.nih.gov/nuccore/KU356193.1> |
| *Gracilaria edulis* | NC_037889.1  MG592725.1 | https://www.ncbi.nlm.nih.gov/nuccore/NC_037889.1  <https://www.ncbi.nlm.nih.gov/nuccore/MG592725.1> |
| *Gracilaria chilensis* | MF401962.1 | <https://www.ncbi.nlm.nih.gov/nuccore/MF401962.1> |
| *Porphyra haitanensis* voucher PH-38 | JQ736808.1 | <https://www.ncbi.nlm.nih.gov/nuccore/JQ736808.1> |
| *Gracilaria textorii* | NC_037892.1  MG592729.1 | https://www.ncbi.nlm.nih.gov/nuccore/NC_037892.1  <https://www.ncbi.nlm.nih.gov/nuccore/MG592729.1> |
| *Pyropia haitanensis* | NC_017751.1 | <https://www.ncbi.nlm.nih.gov/nuccore/NC_017751.1> |
| *Corallina chilensis* voucher UC2050474 | MK598844.1 | <https://www.ncbi.nlm.nih.gov/nuccore/MK598844.1> |
| *Cyanidioschyzon merolae* | NC_000887.3 | <https://www.ncbi.nlm.nih.gov/nuccore/NC_000887.3> |
| *Cyanidioschyzon merolae* mitochondrial DNA | D89861.1 | https://www.ncbi.nlm.nih.gov/nuccore/D89861.1 |
| *Porphyra umbilicalis* | NC_018544.1  JQ388471.1 | https://www.ncbi.nlm.nih.gov/nuccore/NC_018544.1  <https://www.ncbi.nlm.nih.gov/nuccore/JQ388471.1> |
| *Choreocolax polysiphoniae* | NC_032002.1  KX687877.1 | https://www.ncbi.nlm.nih.gov/nuccore/NC_032002.1  <https://www.ncbi.nlm.nih.gov/nuccore/KX687877.1> |
| *Vertebrata lanosa* | NC_032003.1  KX687880.1 | https://www.ncbi.nlm.nih.gov/nuccore/NC_032003.1  <https://www.ncbi.nlm.nih.gov/nuccore/KX687880.1> |
| *Gracilariopsis andersonii* | KX687878.1 | <https://www.ncbi.nlm.nih.gov/nuccore/KX687878.1> |
| *Gracilariophila oryzoide* | KX687879.1 | <https://www.ncbi.nlm.nih.gov/nuccore/KX687879.1> |
| *Gracilariopsis andersonii* | NC_014772.1  HQ586060.1 | https://www.ncbi.nlm.nih.gov/nuccore/NC_014772.1  <https://www.ncbi.nlm.nih.gov/nuccore/HQ586060.1> |
| *Gracilariophila oryzoides* | NC_014771.1  HQ586059.1 | https://www.ncbi.nlm.nih.gov/nuccore/NC_014771.1  <https://www.ncbi.nlm.nih.gov/nuccore/HQ586059.1> |
| *Plocamiocolax pulvinata* | HQ586061.1  NC_014773.1 | https://www.ncbi.nlm.nih.gov/nuccore/HQ586061.1  <https://www.ncbi.nlm.nih.gov/nuccore/NC_014773.1> |
| *Chondrus crispus* | NC_001677.2 | <https://www.ncbi.nlm.nih.gov/nuccore/NC_001677.2> |
| *Gelidium kathyanniae* voucher UC2050581 | NC_040157.1  MG922863.1 | https://www.ncbi.nlm.nih.gov/nuccore/NC_040157.1  <https://www.ncbi.nlm.nih.gov/nuccore/MG922863.1> |
| *Gelidium gabrielsonii* voucher UC2050580 | NC_040155.1 | <https://www.ncbi.nlm.nih.gov/nuccore/NC_040155.1> |
| *Gelidium gabrielsonii* voucher UC2050580 | MG922859.1 | <https://www.ncbi.nlm.nih.gov/nuccore/MG922859.1> |
| *Compsopogon caeruleus* | NC_035351.1  KY083068.1 | https://www.ncbi.nlm.nih.gov/nuccore/NC_035351.1  <https://www.ncbi.nlm.nih.gov/nuccore/KY083068.1> |
| *Thorea hispida* | NC_035158  KY083066.1 | https://www.ncbi.nlm.nih.gov/nuccore/NC_035158  <https://www.ncbi.nlm.nih.gov/nuccore/KY083066.1> |
| *Dasya binghamiae* voucher UC2050572 | NC_031160.1  KX247283.1 | https://www.ncbi.nlm.nih.gov/nuccore/NC_031160.1  <https://www.ncbi.nlm.nih.gov/nuccore/KX247283.1> |
| *Pterocladia mexicana* voucher UC694722 | NC_031842.1  KX427235.1 | https://www.ncbi.nlm.nih.gov/nuccore/NC_031842.1  <https://www.ncbi.nlm.nih.gov/nuccore/KX427235.1> |
| *Pterocladia musciformis* voucher UC1884021 | NC_031843.1  KX427236.1 | https://www.ncbi.nlm.nih.gov/nuccore/NC_031843.1  <https://www.ncbi.nlm.nih.gov/nuccore/KX427236.1> |
| *Pterocladia robusta* voucher UC1884024 | NC_031844.1  KX427237.1 | https://www.ncbi.nlm.nih.gov/nuccore/NC_031844.1  <https://www.ncbi.nlm.nih.gov/nuccore/KX427237.1> |
| *Pterocladia media* voucher UC1884019 | NC_031841.1  KX427234.1 | https://www.ncbi.nlm.nih.gov/nuccore/NC_031841.1  <https://www.ncbi.nlm.nih.gov/nuccore/KX427234.1> |
| *Gelidium sinicola* voucher UC276620 | NC_031840.1  KX427233.1 | https://www.ncbi.nlm.nih.gov/nuccore/NC_031840.1  <https://www.ncbi.nlm.nih.gov/nuccore/KX427233.1> |
| *Gelidium isabelae* voucher UC1884226 | NC_031838.1  KX427231.1 | https://www.ncbi.nlm.nih.gov/nuccore/NC_031838.1  <https://www.ncbi.nlm.nih.gov/nuccore/KX427231.1> |
| *Gelidium galapagense* voucher UC1884224 | NC_031837.1  KX427230.1 | https://www.ncbi.nlm.nih.gov/nuccore/NC_031837.1  <https://www.ncbi.nlm.nih.gov/nuccore/KX427230.1> |
| *Gelidium sclerophyllum* voucher UC1884229 | NC_031839.1  KX427232.1 | https://www.ncbi.nlm.nih.gov/nuccore/NC_031839.1  <https://www.ncbi.nlm.nih.gov/nuccore/KX427232.1> |
| *Gelidium* *crinale* f. *luxurians* voucher UC1878479 | KX427229.1 | <https://www.ncbi.nlm.nih.gov/nuccore/KX427229.1> |
| *Gelidium arborescens* voucher UC93582 | NC_031836.1  KX427228.1 | https://www.ncbi.nlm.nih.gov/nuccore/NC_031836.1  <https://www.ncbi.nlm.nih.gov/nuccore/KX427228.1> |
| *Lithothamnion* sp. | MH281621.1 | <https://www.ncbi.nlm.nih.gov/nuccore/MH281621.1> |
| *Neogoniolithon* *spectabile* | NC_039980.1  MH281624.1 | https://www.ncbi.nlm.nih.gov/nuccore/NC_039980.1  <https://www.ncbi.nlm.nih.gov/nuccore/MH281624.1> |
| *Synarthrophyton* *chejuense* | NC_039979.1  MH281623.1 | https://www.ncbi.nlm.nih.gov/nuccore/NC_039979.1  <https://www.ncbi.nlm.nih.gov/nuccore/MH281623.1> |
| *Rhodogorgon* sp. | MH281625.1 | <https://www.ncbi.nlm.nih.gov/nuccore/MH281625.1> |
| *Pyropia* *perforata* | KF515971.1 | https://www.ncbi.nlm.nih.gov/nuccore/KF515971.1  https://www.ncbi.nlm.nih.gov/nuccore/ |
| *Pyropia* *nitida* voucher UC1966781 | NC_027616.1  KP890080.1 | https://www.ncbi.nlm.nih.gov/nuccore/NC_027616.1  <https://www.ncbi.nlm.nih.gov/nuccore/KP890080.1> |
| *Ahnfeltia* *plicata* | NC_026054.1  KF649303.1 | https://www.ncbi.nlm.nih.gov/nuccore/NC_026054.1  <https://www.ncbi.nlm.nih.gov/nuccore/KF649303.1> |
| *Schimmelmannia* *schousboei* | KJ398162.1 | <https://www.ncbi.nlm.nih.gov/nuccore/KJ398162.1> |
| *Asparagopsis taxiformis* strain CCAP 1341 / 1 | NC_026843.1  KJ398158.1 | https://www.ncbi.nlm.nih.gov/nuccore/NC_026843.1  <https://www.ncbi.nlm.nih.gov/nuccore/KJ398158.1> |
| *Gelidium elegans* | NC_026053.1  KF290995.1 | https://www.ncbi.nlm.nih.gov/nuccore/NC_026053.1  <https://www.ncbi.nlm.nih.gov/nuccore/KF290995.1> |
| *Hildenbrandia rubra* | NC_026055.1  KF649304.1 | https://www.ncbi.nlm.nih.gov/nuccore/NC_026055.1  <https://www.ncbi.nlm.nih.gov/nuccore/KF649304.1> |
| *Plocamium cartilagineum* | KJ398160.1 | <https://www.ncbi.nlm.nih.gov/nuccore/KJ398160.1> |
| *Palmaria palmata* | NC_026056.1  KF649305.1 | https://www.ncbi.nlm.nih.gov/nuccore/NC_026056.1  <https://www.ncbi.nlm.nih.gov/nuccore/KF649305.1> |
| *Galdieria sulphuraria* strain 074W | NC_024666.1 | <https://www.ncbi.nlm.nih.gov/nuccore/NC_024666.1> |
| *Corallina ferreyrae* voucher UC1404138 | NC_041635.1  MK408747.1 | https://www.ncbi.nlm.nih.gov/nuccore/NC_041635.1  <https://www.ncbi.nlm.nih.gov/nuccore/MK408747.1> |
| *Gracilariopsis lemaneiformis* | JQ071938.1 | <https://www.ncbi.nlm.nih.gov/nuccore/JQ071938.1> |
| *Grateloupia filicina* | NC_037842.1  MG598532.1 | https://www.ncbi.nlm.nih.gov/nuccore/NC_037842.1  <https://www.ncbi.nlm.nih.gov/nuccore/MG598532.1> |
| *Porphyra purpurea* | AF114794.1  NC_002007.1 | https://www.ncbi.nlm.nih.gov/nuccore/AF114794.1  <https://www.ncbi.nlm.nih.gov/nuccore/NC_002007.1> |
| *Gracilaria* *tenuistipitatavar*. liui | MG592728.1 | <https://www.ncbi.nlm.nih.gov/nuccore/MG592728.1> |
| *Gracilaria tenuistipitata* | NC_037891.1  MG592727.1 | https://www.ncbi.nlm.nih.gov/nuccore/NC_037891.1  <https://www.ncbi.nlm.nih.gov/nuccore/MG592727.1> |
| *Pyropia tenera* | NC_021475.1  KC750917.1 | https://www.ncbi.nlm.nih.gov/nuccore/NC_021475.1  <https://www.ncbi.nlm.nih.gov/nuccore/KC750917.1> |
| *Batrachospermum macrosporum* strain J.0232 | NC_039404.1  MG787094.1 | https://www.ncbi.nlm.nih.gov/nuccore/NC_039404.1  <https://www.ncbi.nlm.nih.gov/nuccore/MG787094.1> |
| *Sheathia* *arcuata* | NC_035349.1  KY083064.1 | https://www.ncbi.nlm.nih.gov/nuccore/NC_035349.1  <https://www.ncbi.nlm.nih.gov/nuccore/KY083064.1> |
| *Sirodotia* *delicatula* strain MOP3 | NC_039407.1  MG787099.1 | https://www.ncbi.nlm.nih.gov/nuccore/NC_039407.1  <https://www.ncbi.nlm.nih.gov/nuccore/MG787099.1> |
| *Kumanoa* *mahlacensis* strain J.0229 | NC_039406.1  MG787096.1 | https://www.ncbi.nlm.nih.gov/nuccore/NC_039406.1  <https://www.ncbi.nlm.nih.gov/nuccore/MG787096.1> |
| *Kumanoa* *ambigua* strain MOP2 | NC_039405.1  MG787095.1 | https://www.ncbi.nlm.nih.gov/nuccore/NC_039405.1  <https://www.ncbi.nlm.nih.gov/nuccore/MG787095.1> |
| *Paralemanea* sp. strain HEC3068 | MG787097.1 | <https://www.ncbi.nlm.nih.gov/nuccore/MG787097.1> |
| *Grateloupia angusta* | NC_023094.1  KC875853.1 | https://www.ncbi.nlm.nih.gov/nuccore/NC_023094.1  <https://www.ncbi.nlm.nih.gov/nuccore/KC875853.1> |
| *Wildemania schizophylla* voucher Hollenberg 1914 (UC 306400) | NC_024579.1  KJ689442.1 | https://www.ncbi.nlm.nih.gov/nuccore/NC_024579.1  <https://www.ncbi.nlm.nih.gov/nuccore/KJ689442.1> |
| *Corallina officinalis* | NC_033904.1  KU641510.1 | https://www.ncbi.nlm.nih.gov/nuccore/NC_033904.1  <https://www.ncbi.nlm.nih.gov/nuccore/KU641510.1> |
| *Batrachospermaceae* sp. DIW-2017a voucher BHO:A-1418 | NC_036231.1  MF488959.1 | https://www.ncbi.nlm.nih.gov/nuccore/NC_036231.1  <https://www.ncbi.nlm.nih.gov/nuccore/MF488959.1> |
| *Gelidium vagum* | NC_023077.1  KC875854.1 | https://www.ncbi.nlm.nih.gov/nuccore/NC_023077.1  <https://www.ncbi.nlm.nih.gov/nuccore/KC875854.1> |
| *Gracilaria changii* | KY009863.1 | <https://www.ncbi.nlm.nih.gov/nuccore/KY009863.1> |
| *Gracilariopsis chorda* | NC_023251.1  KC875851.1 | https://www.ncbi.nlm.nih.gov/nuccore/NC_023251.1  <https://www.ncbi.nlm.nih.gov/nuccore/KC875851.1> |
| *Rhodymenia pseudopalmata* | NC_023252.1  KC875852.1 | https://www.ncbi.nlm.nih.gov/nuccore/NC_023252.1  <https://www.ncbi.nlm.nih.gov/nuccore/KC875852.1> |
| *Sporolithon durum* | NC_023454.1  KF186230.1 | https://www.ncbi.nlm.nih.gov/nuccore/NC_023454.1  <https://www.ncbi.nlm.nih.gov/nuccore/KF186230.1> |
| *Calliarthron tuberculosum* | NC_027061.1  KR005619.1 | https://www.ncbi.nlm.nih.gov/nuccore/NC_027061.1  <https://www.ncbi.nlm.nih.gov/nuccore/KR005619.1> |
| *Pyropia yezoensis* | NC_017837.1  KF561997.1 | https://www.ncbi.nlm.nih.gov/nuccore/NC_017837.1  <https://www.ncbi.nlm.nih.gov/nuccore/KF561997.1> |
| *Pyropia yezoensis* voucher RZ-58 | JQ736809.1 | <https://www.ncbi.nlm.nih.gov/nuccore/JQ736809.1> |
| *Gracilaria spinulosa* | NC_037890.1  MG592726.1 | https://www.ncbi.nlm.nih.gov/nuccore/NC_037890.1  <https://www.ncbi.nlm.nih.gov/nuccore/MG592726.1> |
| *Kappaphycus alvarezii* | NC_031814.1  KU885455.1 | https://www.ncbi.nlm.nih.gov/nuccore/NC_031814.1  <https://www.ncbi.nlm.nih.gov/nuccore/KU885455.1> |
| *Eucheuma denticulatum* | NC_036432.1  MF680515.1 | https://www.ncbi.nlm.nih.gov/nuccore/NC_036432.1  <https://www.ncbi.nlm.nih.gov/nuccore/MF680515.1> |
| *Betaphycus gelatinus* | NC_036431.1  MF680514.1 | https://www.ncbi.nlm.nih.gov/nuccore/NC_036431.1  <https://www.ncbi.nlm.nih.gov/nuccore/MF680514.1> |
| *Pyropia fucicola* | NC_024288.1  KJ708762.1 | https://www.ncbi.nlm.nih.gov/nuccore/NC_024288.1  <https://www.ncbi.nlm.nih.gov/nuccore/KJ708762.1> |
| *Pyropia perforata* voucher UC 95735 | KJ708768.1 | <https://www.ncbi.nlm.nih.gov/nuccore/KJ708768.1> |
| *Pyropia perforata* voucher PB-A 684 | KJ708766.1 | <https://www.ncbi.nlm.nih.gov/nuccore/KJ708766.1> |
| *Pyropia perforata* voucher LD-Ag 13031 | KJ708767.1 | <https://www.ncbi.nlm.nih.gov/nuccore/KJ708767.1> |
| *Pyropia perforata* voucher LD-Ag 13032 | KJ708769.1 | <https://www.ncbi.nlm.nih.gov/nuccore/KJ708769.1> |
| *Pyropia perforata* voucher LD-Ag 13038 | KJ708764.1 | <https://www.ncbi.nlm.nih.gov/nuccore/KJ708764.1> |
| *Pyropia perforata* voucher UC 807662 | KF515975.1 | <https://www.ncbi.nlm.nih.gov/nuccore/KF515975.1> |
| *Pyropia perforate*voucher VK-11-00061 | KF515974.1 | <https://www.ncbi.nlm.nih.gov/nuccore/KF515974.1> |
| *Pyropia perforata* voucher UC 2019902 | KJ708761.1 | <https://www.ncbi.nlm.nih.gov/nuccore/KJ708761.1> |
| *Pyropia perforata* voucher UC 2019901 | KJ708772.1 | <https://www.ncbi.nlm.nih.gov/nuccore/KJ708772.1> |
| *Pyropia perforata* voucher UC 2019900 | KJ708771.1 | <https://www.ncbi.nlm.nih.gov/nuccore/KJ708771.1> |
| *Pyropia perforata* voucher UC 1450590 | KJ708770.1 | <https://www.ncbi.nlm.nih.gov/nuccore/KJ708770.1> |
| *Pyropia kanakaensis* voucher UC 1863890 | NC_024289.1  KJ708765.1 | https://www.ncbi.nlm.nih.gov/nuccore/NC_024289.1  <https://www.ncbi.nlm.nih.gov/nuccore/KJ708765.1> |
| *Pyropia kanakaensis* voucher WTU 255136 | KJ708763.1 | <https://www.ncbi.nlm.nih.gov/nuccore/KJ708763.1> |
| *Bangia atropurpurea* | NC_030222.1 | <https://www.ncbi.nlm.nih.gov/nuccore/NC_030222.1> |
| *Bangia fuscopurpurea* isolate OUPT01 | NC_026905.1  KP710961.1 | https://www.ncbi.nlm.nih.gov/nuccore/NC_026905.1  <https://www.ncbi.nlm.nih.gov/nuccore/KP710961.1> |
| *Cyanidiaceae* sp. MX-AZ01 | KJ569774.1 | <https://www.ncbi.nlm.nih.gov/nuccore/KJ569774.1> |
| *Sheathia* *arcuata* strain J.0228 | MG787098.1 | <https://www.ncbi.nlm.nih.gov/nuccore/MG787098.1> |
| *Balbiania* *investiens* | NC_042172.1  MH026109.1 | https://www.ncbi.nlm.nih.gov/nuccore/NC_042172.1  <https://www.ncbi.nlm.nih.gov/nuccore/MH026109.1> |
| *Gracilariopsis longissima* | NC_039150.1  MH396023.1 | https://www.ncbi.nlm.nih.gov/nuccore/NC_039150.1  <https://www.ncbi.nlm.nih.gov/nuccore/MH396023.1> |
| *Gracilariopsis mclachlanii* | NC_039151.1  MH396024.1 | https://www.ncbi.nlm.nih.gov/nuccore/NC_039151.1  <https://www.ncbi.nlm.nih.gov/nuccore/MH396024.1> |
| *Gracilariopsis heteroclada* | NC_038101.1  MF372958.1 | https://www.ncbi.nlm.nih.gov/nuccore/NC_038101.1  <https://www.ncbi.nlm.nih.gov/nuccore/MF372958.1> |
| *Gracilaria vermiculophylla* | MH396022.1 | <https://www.ncbi.nlm.nih.gov/nuccore/MH396022.1> |
| *Gracilaria vermiculophylla* voucher 201104055 | KJ526627.1 | <https://www.ncbi.nlm.nih.gov/nuccore/KJ526627.1> |
| *Gracilaria vermiculophylla* voucher 201103102 | NC_027064.1  KJ526626.1 | https://www.ncbi.nlm.nih.gov/nuccore/NC_027064.1  <https://www.ncbi.nlm.nih.gov/nuccore/KJ526626.1> |
| *Gracilaria chilensis* voucher CNU050183 | NC_026831.1  KP728466.1 | https://www.ncbi.nlm.nih.gov/nuccore/NC_026831.1  <https://www.ncbi.nlm.nih.gov/nuccore/KP728466.1> |
| *Gracilaria salicornia* | NC_023784.1  KF824534.1 | https://www.ncbi.nlm.nih.gov/nuccore/NC_023784.1  <https://www.ncbi.nlm.nih.gov/nuccore/KF824534.1> |
| *Gracilaria salicornia* isolate GS1 | KT373903.1 | <https://www.ncbi.nlm.nih.gov/nuccore/KT373903.1> |
| *Gracilaria caudata* voucher SPF57390 | NC_039146.1  MH396017.1 | https://www.ncbi.nlm.nih.gov/nuccore/NC_039146.1  <https://www.ncbi.nlm.nih.gov/nuccore/MH396017.1> |
| *Gracilaria changii* isolate GC2 | NC_034681.1  KX980031.1 | https://www.ncbi.nlm.nih.gov/nuccore/NC_034681.1  <https://www.ncbi.nlm.nih.gov/nuccore/KX980031.1> |
| *Gracilaria gracilis* voucher SPF:55734 | NC_039148.1  MH396019.1 | https://www.ncbi.nlm.nih.gov/nuccore/NC_039148.1  <https://www.ncbi.nlm.nih.gov/nuccore/MH396019.1> |
| *Gracilaria ferox* voucher SPF:26055 | NC_039147.1  MH396018.1 | https://www.ncbi.nlm.nih.gov/nuccore/NC_039147.1  <https://www.ncbi.nlm.nih.gov/nuccore/MH396018.1> |
| *Gracilaria tenuistipitata* voucher SPF:56193 | MH396021.1 | <https://www.ncbi.nlm.nih.gov/nuccore/MH396021.1> |
| *Gracilaria chouae* | NC_038211.1  MG733298.1 | https://www.ncbi.nlm.nih.gov/nuccore/NC_038211.1  <https://www.ncbi.nlm.nih.gov/nuccore/MG733298.1> |
| *Gracilaria chouae* | MF351970.1 | <https://www.ncbi.nlm.nih.gov/nuccore/MF351970.1> |
| *Melanthalia intermedia* | NC_039152.1  MH396025.1 | https://www.ncbi.nlm.nih.gov/nuccore/NC_039152.1  <https://www.ncbi.nlm.nih.gov/nuccore/MH396025.1> |
| *Hydropuntia rangiferina* voucher SPF:56055 | NC_039149.1  MH396020.1 | https://www.ncbi.nlm.nih.gov/nuccore/NC_039149.1  <https://www.ncbi.nlm.nih.gov/nuccore/MH396020.1> |
| *Mastocarpus papillatus* voucher UC2050562 | NC_031166.1  KX525587.1 | https://www.ncbi.nlm.nih.gov/nuccore/NC_031166.1  <https://www.ncbi.nlm.nih.gov/nuccore/KX525587.1> |
| *Gelidium coulteri* voucher UC2050582 | NC_040985.1  MG922857.1 | https://www.ncbi.nlm.nih.gov/nuccore/NC_040985.1  <https://www.ncbi.nlm.nih.gov/nuccore/MG922857.1> |
| *Renouxia* sp. | MH281622.1 | <https://www.ncbi.nlm.nih.gov/nuccore/MH281622.1> |
| *Porolithon onkodes* voucher TRH A26-1494 | NC_038145.1  KY212107.1 | https://www.ncbi.nlm.nih.gov/nuccore/NC_038145.1  <https://www.ncbi.nlm.nih.gov/nuccore/KY212107.1> |
| *Cyanidioschyzon merolae* 10D-T | LC519602.1 | <https://www.ncbi.nlm.nih.gov/nuccore/LC519602.1> |
| *Palmaria decipiens* voucher LMS000004 | MN967053.1 | <https://www.ncbi.nlm.nih.gov/nuccore/MN967053.1> |
| *Gracilaria tenuistipitata* | KY054924.1 | <https://www.ncbi.nlm.nih.gov/nuccore/KY054924.1> |
| *Palmaria palmata* | AP019296.1 | <https://www.ncbi.nlm.nih.gov/nuccore/AP019296.1> |
| *Gloiopeltis furcata* | NC_044414.1 | <https://www.ncbi.nlm.nih.gov/nuccore/NC_044414.1> |
| *Pyropia yezoensis* strain RZ-58 | MK695879.1 | <https://www.ncbi.nlm.nih.gov/nuccore/MK695879.1> |

# Table S 3. Algal organelle genome data sequenced and published by our laboratory (MOGBL)

| Species | Mitochondrial genome accession number | Publication | Plastid genome accession number | Publication |
| --- | --- | --- | --- | --- |
| *Saccharina japonica × Saccharina latissima* ‘Rongfu’ | JF937591 | [1] | None | None |
| *Saccharina japonica × Saccharina latissima* ‘Rongfu2009’ | MK170139 | [2] | MK058524 | Unpublished |
| *Saccharina japonica × Saccharina latissima* ‘Rongfu2010’ | MK170140 | [2] | MK058525 | Unpublished |
| *Saccharina japonica × Saccharina latissima* ‘Rongfu2011’ | MK170141 | [2] | MK058526 | Unpublished |
| *Saccharina longissima* | JN099684 | [3] | None | None |
| *Saccharina japonica × latissima* ‘Ailunwan’ | KU556731 | [4] | None | None |
| *Saccharina japonica × latissima* ‘Pingbancai’ | KX073817 | [5] | None | None |
| *Saccharina japonica × latissima* ‘Hainong No.1’ | MF622087 | [6] | None | None |
| *Saccharina japonica* ‘Zaohoucheng’ | KX073816 | [7] | None | None |
| *Saccharina japonica* ‘Fujian' | KX073815 | [8] | None | None |
| *Saccharina japonica × latissima* ‘Xinbenniu’ | MG712780 | [9] | None | None |
| *Saccharina japonica × longissima* ‘Dongfang No. 3’ | MG712777 | Unpublished | None | None |
| *Saccharina japonica* ‘Dongfang No. 6’ | MG712776 | [10] | None | None |
| *Saccharina japonica × latissima* ‘Sanhai’ | MG712779 | Unpublished | None | None |
| *Saccharina sp.* ‘Haiyi No. 1’ | MG712778 | [11] | None | None |
| *Eucheuma denticulatum* | MF680515 | [12] | MN240357 | Unpublished |
| *Kappaphycus striatum* | None | None | MN240358 | Unpublished |
| *Betaphycus gelatinae* | MF680514 | [12] | MN240356 | Unpublished |
| *Gracilaria chouae* | MF351970  MG733298 | [13] | None | None |
| *Kappaphycus alvarezii* | KU885455 | [12] | KU892652 | [14] |
| *Gracilaria edulis* | MG592725 | [15] | MN053318 | [16] |
| *Gracilaria textorii* | MG592729 | [17] | MN053320 | [18] |
| *Gracilaria spinulosa* | MG592726 | [19] | MN053319 | [20] |
| *Gracilaria tenuistipitata var. liui* | MG592728 | [21] | None | None |
| *Gracilaria tenuistipitata* | MG592727 | [22] | None | None |
| *Gracilaria chilensis* | MF401962 | [23] | MF401963 | [24] |
| *Gracilariopsis heteroclada* | MF372958 | Unpublished | MF372957 | [25] |
| *Grateloupia filicina* | MG598532 | [26] | MG598531 | [27] |
| *Caulerpa lentillifera* | MN201586 | [28] | MN201587 | Unpublished |
| *Codium fragile* | MT050449 | Unpublished | MN733705 | [29] |
| *Endarachne binghamiae* | MF374731 | [30] | None | None |
| *Sargassum fusiforme* | MN883537 | [31] | MN794016 | [32] |
| *Costaria costata* | NC_023506 | [33] | NC_028502 | [34] |
| *Undaria pinnatifida* | KF319031 | [35] | NC_028503 | [36] |
| *Sargassum horneris* | None | None | MN265366 | [37] |

References:

1. Zhang,J., Li,N., Zhang,Z., Liu,T. (2011) Structure analysis of the complete mitochondrial genome in cultivation variety 'rongfu'. Journal of Ocean University of China, 10(04):351-356.

2. Yu,Y., Zhang,J., Jia,S., Wang,G., Liu,T. & Wang,X. (2019) The complete mitogenome of three continuous generations of ‘Rongfu’: a crucial Saccharina cultivation variety. Mitochondrial DNA Part B, 4:1, 744-745.

3. Zhang,J., Wang,X., Liu,C., Jin,Y. and Liu,T. (2013) The complete mitochondrial genomes of two brown algae (laminariales, phaeophyceae) and phylogenetic analysis within laminaria. J Appl Phycol., 25(4), 1247-1253.

4. Zhang,J., Tao,L., Zhang,L. & Liang,J. (2016) Complete mitogenome of saccharina cultivation variety 'ailunwan'( saccharina japonica × latissima ). Mitochondrial DNA Part B, 1(1), 406-407.

5. Zhang,J., Gao,B., Liu,T., Zhang,L. & Liu,N. (2016) The complete mitogenome of 'pingbancai', an important economic saccharina cultivation variety. Mitochondrial DNA Part B, 1:1, 470-471.

6. Liu,N., Zhang,J., Liu,C., Li,Y., Liu,T. & Chi,S. (2017) Complete mitochondrial genome of Saccharina cultivar ‘Hainong No.1’ (Saccharina japonica × latissima). Mitochondrial DNA Part B, 2:2, 672-673.

7. Zhang,J., Li,N., LiuN. & Liu T. (2017) The complete mitogenome of ‘Zaohoucheng’, an important Saccharina japonica cultivar in China. Mitochondrial DNA Part B, 2:2, 656-657.

8. Zhang,J., Liu,N. & Liu,T. (2018) The complete mitogenome of ‘Fujian’: an important Saccharina japonica cultivar of South China. Mitochondrial DNA Part B, 3:2, 588-589.

9. Zhang,J., Liu,T. & Liu,N. (2019) The complete mitogenome of Saccharina cultivar ‘Xinbenniu’ (Saccharina japonica ×latissima) and phylogenetic analysis. Mitochondrial DNA Part B, 4:1, 1193-1194.

10. Zhang,J., Liu,N. & Liu,T. (2019) The complete mitogenome of Saccharina cultivar ‘Dongfang No.6’ and its phylogenetic analysis. Mitochondrial DNA Part B, 4:1, 1161-1162.

11. Liu,P. & Zhang,J. (2020) The complete mitogenome of Saccharina cultivar ‘Haiyi No.1’ (Saccharina japonica × latissima) and the phylogenetic analysis. Mitochondrial DNA Part B, 5:1, 449-450.

12. Li,Y., Liu,N., Wang,X., Tang,X., Zhang,L., Meinita,M.D.N., et al. (2018) Comparative genomics and systematics of betaphycus, eucheuma, and kappaphycus (solieriaceae: rhodophyta) based on mitochondrial genome. Journal of Applied Phycology, 30, 3435–3443.

13. Tang,X., Jia,X., Zhang,J. & Liu,T. (2019) The complete mitogenome of gracilaria chouae and its phylogenetic analysis. Mitochondrial DNA Part B, 4:2, 2786-2787.

14. Liu,N., Zhang,L., Tang,X.,Wang,X., Meinita,M., Wang,G., Chen,W., Liu,T. (2019). Correction to: Complete plastid genome of Kappaphycus alvarezii: insights of large-scale rearrangements among Florideophyceae plastid genomes. Journal of Applied Phycology, 31, 1-2.

15. Mary,G.C.S., Maria,D.N.M., Tang,X., Chen,W., Yin, H., Liu,C., Jin,Y., Chi,S., Li,Y. & Liu,T. (2020) Complete sequence of mitochondrial DNA of Gracilaria edulis (Rhodophyta). Mitochondrial DNA Part B, 5:2, 1128-1129.

16. Liu,T., Tang,X., Jia,X., Wu,X., Huang,M., Zeng,J. & Chen,W.(2019) The complete plastid genome and phylogenetic analysis of Gracilaria edulis. Mitochondrial DNA Part B, 4:2, 2598-2599.

17. Yuan,X. (2018) The complete mitochondrial genome of gracilaria textorii (gracilariales, florideophyceae). Mitochondrial DNA Part B, 3:1, 438-439.

18. Chen,W., Liu,T., Tang,X., Jia,X. & Wu,X. (2019). The complete plastid genome and phylogenetic analysis of gracilaria textorii. Mitochondrial DNA Part B, 4:2, 2608-2609.

19. Wang,S., Zhang,J., Qian,H., Chen,W., Liu,T. & Tang,X. (2019) Complete mitochondrial genome and phylogenetic analysis of Gracilaria spinulosa. Mitochondrial DNA Part B, 4:1, 2046-2047.

20. Liu,T., Tang,X., Jia,X., Wu,X. & Chen,W. (2019) The complete plastid genome and phylogenetic analysis of gracilaria edulis. Mitochondrial DNA Part B, 4:2, 2606-2607.

21. Tang,X., Li,Y., Chen,W., Liu,T. & Zhang,J. (2018) Complete sequence of mitochondrial DNA of Gracilaria tenuistipitata var. liui (Rhodophyta). Mitochondrial DNA Part B, 3:2, 1203-1204

22. Liu,N., Li,Y., Liu,C., Liu,T. & Chen, W. (2018) Complete sequence of mitochondrial dna of gracilaria tenuistipitata (rhodophyta). Mitochondrial DNA Part B, 3:2, 814-815.

23. Liu,N., Wang,G., Li,Y., Zhang,L., Maria,D.N.M., Chen,W., Liu, T. & Chi,S. (2017) The complete mitochondrial genome of the economic red alga, Gracilaria chilensis. Mitochondrial DNA Part B, 2:2, 716-717.

24. Wang,G., Liu,N., Li,Y., Zhang,L., Maria,D.N.M., Chen,W., Liu,T. & Chi,S. (2020) The complete plastid genome and phylogenetic analysis of Gracilaria chilensis. Mitochondrial DNA Part B, 5:2, 1282-1283.

25. Tang,X., Jia,X., Zhang, J. & Liu,T. (2019) The complete mitogenome of gracilaria chouae and its phylogenetic analysis. Mitochondrial DNA Part B, 4:2, 2786-2787.

26. Li,Y., Meinita,M.D.N., Liu,T., Chi,S. & Yin, H. (2018) Complete sequences of the mitochondrial DNA of the Grateloupia filicina (Rhodophyta). Mitochondrial DNA Part B, 3:1, 76-77.

27. Zhang,J., Tang,X., Chen,W., Liu,T. & Li, Y. (2018) The complete plastid genome of Grateloupia filicina (Rhodophyta) and phylogenetic analysis, Mitochondrial DNA Part B, 3:2, 1172-1173.

28. Jia,X., Liu,T., Wang,X., Tang,X. & Jin,Y. (2019) The complete mitogenome of Caulerpa lentillifera and its phylogenetic analysis. Mitochondrial DNA Part B, 4:2, 3169-3170.

29. Jia,X., Liu,T., Li,R. & Jin,Y. (2020) The complete plastid genome and phylogenetic analysis of Codium fragile. Mitochondrial DNA Part B, 5:1, 402-403.

30. Li,Y., Liu,N., Yin,H., Liu,C., Zhang,L., Jin,Y., Wang,H., Chi,S. & Liu,T. (2018) Complete sequences of the mitochondrial DNA of the Petalonia binghamiae. Mitochondrial DNA Part B, 3:1, 95-96.

31. Liu,T., Cui,Y., Jia,X., Chen,B., Ma,Z., Zou,H., Wang,S. & Wu,M. (2020) The complete mitochondrial genome of brown algae: Sargassum fusiforme (Harvey) Setchell. Mitochondrial DNA Part B, 5:1, 830-831.

32. Liu,T., Cui,Y., Jia,X., Chen,B., Ma,Z., Zou,H., Wang,S. & Wu,M. (2020) The complete chloroplast genome of Sargassum fusiforme. Mitochondrial DNA Part B, 5:1, 576-577.

33. Qu,J., Liu,C., Wang,X., Zhang,Z., Chi,S. & Liu, T. (2015) Complete mitochondrial genome of Costaria costata shows conservative evolution in Laminariales. Mitochondrial DNA, 26(6), 919–920.

34. Zhang,L., Wang,X., Liu,T., Wang,H., Wang,G., Chi,S., & Liu,C. (2015) Complete Plastid Genome of the Brown Alga Costaria costata (Laminariales, Phaeophyceae). PloS one, 10(10), e0140144.

35. Li,T., Qu, J., Feng,Y., Liu,C., Chi,S., & Liu,T. (2015) Complete mitochondrial genome of Undaria pinnatifida (Alariaceae, Laminariales, Phaeophyceae). Mitochondrial DNA, 26(6), 953–954.

36. Zhang,L., Wang,X., Liu,T., Wang,G., Chi,S., Liu, C. & Wang, H. (2015) Complete Plastid Genome Sequence of the Brown Alga Undaria pinnatifida. PloS one, 10(10), e0139366.

37. Cui,Y., Liu,T., Wang,X., Qu,J. & Jia,X. (2019) The complete chloroplast genome of Sargassum horneri and its phylogenetic analysis. Mitochondrial DNA Part B, 4:2, 3312-3313.
